# Heterozygosity–fitness correlations in a wild mammal population: accounting for parental and environmental effects

**DOI:** 10.1002/ece3.1112

**Published:** 2014-05-27

**Authors:** Geetha Annavi, Christopher Newman, Christina D Buesching, David W Macdonald, Terry Burke, Hannah L Dugdale

**Affiliations:** 1 Wildlife Conservation Research Unit, Department of Zoology, Recanati-Kaplan Centre, University of OxfordTubney House, Abingdon Road, Tubney, Abingdon, Oxfordshire, OX13 5QL, U.K; 2 NERC Biomolecular Analysis Facility, Department of Animal and Plant Sciences, University of SheffieldSheffield, S10 2TN, U.K; 3 Faculty of Science, Department of Biology, University of Putra MalaysiaUPM 43400, Serdang, Selangor, Malaysia; 4 Theoretical Biology, Centre for Ecological and Evolutionary Studies, University of GroningenPO Box 11103, 9700 CC, Groningen, The Netherlands; 5 Behavioural Ecology and Self-Organization, Centre for Ecological and Evolutionary Studies, University of GroningenPO Box 11103, 9700 CC, Groningen, The Netherlands

**Keywords:** Capture–mark–recapture survival analysis, European badger, heterozygosity–fitness correlations, inbreeding depression, *Meles meles*, paternal effects

## Abstract

HFCs (heterozygosity–fitness correlations) measure the direct relationship between an individual's genetic diversity and fitness. The effects of parental heterozygosity and the environment on HFCs are currently under-researched. We investigated these in a high-density U.K. population of European badgers (*Meles meles*), using a multimodel capture–mark–recapture framework and 35 microsatellite loci. We detected interannual variation in first-year, but not adult, survival probability. Adult females had higher annual survival probabilities than adult males. Cubs with more heterozygous fathers had higher first-year survival, but only in wetter summers; there was no relationship with individual or maternal heterozygosity. Moist soil conditions enhance badger food supply (earthworms), improving survival. In dryer years, higher indiscriminate mortality rates appear to mask differential heterozygosity-related survival effects. This paternal interaction was significant in the most supported model; however, the model-averaged estimate had a relative importance of 0.50 and overlapped zero slightly. First-year survival probabilities were not correlated with the inbreeding coefficient (*f*); however, small sample sizes limited the power to detect inbreeding depression. Correlations between individual heterozygosity and inbreeding were weak, in line with published meta-analyses showing that HFCs tend to be weak. We found support for general rather than local heterozygosity effects on first-year survival probability, and *g2* indicated that our markers had power to detect inbreeding. We emphasize the importance of assessing how environmental stressors can influence the magnitude and direction of HFCs and of considering how parental genetic diversity can affect fitness-related traits, which could play an important role in the evolution of mate choice.

## Introduction

Genetic diversity within populations is fundamental to the operation of natural selection. Understanding how genetic diversity is associated with fitness is thus essential for comprehending and predicting evolutionary processes (Sterns and Hoekstra 2005; Ellegren and Sheldon 2008). Genetic diversity manifests in individuals as heterozygosity, which has been found to correlate with fitness-related traits, such as survival probability (Charpentier et al. 2008), reproductive success (Slate et al. 2000), and disease resistance (Acevedo-Whitehouse et al. 2005). Three hypotheses have been advanced to explain heterozygosity–fitness correlations (HFCs) (David 1998):

- The general effect hypothesis (Hansson and Westerberg 2002) proposes that HFCs arise due to inbreeding or outbreeding depression. For example, inbreeding reduces heterozygosity on a genome-wide scale, which increases the probability that deleterious mutations are expressed (Keller and Waller 2002). This can lead to inbreeding depression, where the offspring of related parents exhibit lower fitness than do offspring of unrelated parents.
- The direct effect hypothesis (David 1998) proposes that HFCs arise due to functional overdominance at scored loci that are under direct selection. Functional overdominance occurs when a heterozygote has intrinsically higher fitness than that of either homozygote. This hypothesis is generally rejected when using microsatellites, because microsatellites are usually assumed to be neutral markers, located in noncoding regions of the genome (Jarne and Lagoda 1996), so effects are generally indirect (local) rather than direct. Nevertheless, some microsatellites have a functional role in structural and metabolic DNA processes, such as the regulation of gene activity, that is, DNA replication and recombination (Li et al. 2002).
- The local effect hypothesis (David 1998) proposes that associative overdominance explains HFCs, where some loci are in linkage disequilibrium with functional loci. Local effects can be weak, however, and many studies may have overestimated these by using inappropriate statistical tests (Szulkin et al. 2010).

Differential fitness can arise through parental as well as individual effects (Kirkpatrick and Lande 1989), evidenced as correlations between offspring fitness and parental heterozygosity (Richardson et al. 2004; Brouwer et al. 2007; Fossøy et al. 2007; Olano-Marin et al. 2011). HFCs based on parental heterozygosity and offspring fitness could manifest through direct or indirect effects. Cross-fostering has demonstrated that maternal HFCs can be mediated as a genetic effect, rather than an effect of maternal care in the cross-fostered environment, potentially linked to loci that affect egg size, hormones, immunity, or antibodies (Brouwer et al. 2007). The mechanisms behind paternal HFCs are less clear and have been hypothesized to arise through inbreeding effects on paternal care (Olano-Marin et al. 2011). Alternatively, females might invest differentially in offspring according to mate quality, although Sardell et al. (2014) reported no correlation with paternal heterozygosity.

Heterozygosity–fitness correlations might only be detected under specific environmental conditions. Inbreeding depression tends to increase linearly with the magnitude of stress induced by environmental conditions (Fox and Reed 2011). As a consequence, HFCs might manifest more strongly across populations under stressful conditions (e.g., Lesbarreres et al. 2005; Da Silva et al. 2006; Brouwer et al. 2007). Conversely, HFCs might only be detected during favorable conditions; if unfavorable conditions exert stronger selection than heterozygosity, then HFCs will be masked (Harrison et al. 2011). The direction of the interaction may vary with the traits studied.

Heterozygosity–fitness correlations have become increasingly popular tools for quantifying inbreeding depression in populations in which pedigrees have not been derived (Grueber et al. 2008; Chapman et al. 2009). Nevertheless, empirical evidence currently indicates that correlations between molecular heterozygosity (e.g., standardized multilocus heterozygosity: Coltman et al. 1999) and the coefficient of inbreeding (*f*) tend to be weak (Coltman and Slate 2003), even when estimated using relatively large numbers (16–23) of microsatellite loci. When investigating HFCs, it is therefore important to choose a measure of heterozygosity that reflects inbreeding reliably (Coltman and Slate 2003), and quantify the power to detect inbreeding or outbreeding. Detection of HFCs is greater: (1) in populations with a higher variance in *f,* as incestuous matings yield identity disequilibrium of loci across the genome (Slate et al. 2004; however, see Chapman et al. 2009); (2) when more markers are used to estimate heterozygosity (Balloux et al. 2004); and (3) under specific environmental conditions.

We investigated HFCs in a study system that fulfills these three critical criteria. This high-density population of European badgers (*Meles meles*) has a genetically derived pedigree that includes inbreeding events (5% of matings are incestuous: Dugdale 2007), enabling variance in *f* to be quantified. We have also genotyped individuals in this population using 35 microsatellite loci (Table S1), which is more than most other HFC studies (Chapman et al. 2009; Miller and Coltman 2014). Furthermore, we have a substantial database detailing the life histories of badgers in this population, which has revealed that temperature and rainfall in both spring and summer impact on fitness components (Macdonald and Newman 2002; Macdonald et al. 2010; Nouvellet et al. 2013).

To test for a relationship between genetic diversity and fitness, we examined whether individual heterozygosity and *f* predicted first-year survival probability (i.e., survival from first trapping (minimum age 15 weeks) to 1 year of age). We examined the combined effects of individual, maternal, and paternal heterozygosity on first-year survival probability. May rainfall extremes interact with juvenile parasitic infection, affecting juvenile mortality rate (Macdonald et al. 2010; Nouvellet et al. 2013); therefore, we also included climatic effects on first-year survival probability, while controlling for parasitic infection levels statistically.

This enabled us to test whether the general, direct, or local effect hypotheses provided the greatest explanatory power for HFCs in this study population. Should heterozygosity at any single locus correlate more strongly with fitness-related traits than multilocus heterozygosity, this would be consistent with a direct or local effect. Alternatively, if multilocus heterozygosity reflects genome-wide heterozygosity – thus predicting *f* – this would be consistent with a general effect.

## Materials and Methods

### Study site, and species and data collection

This study was conducted at Wytham Woods, 6 km northwest of Oxford in southern England (51°46′24″N, 1°20′04″W), which comprises 415 ha of mixed deciduous/coniferous secondary and ancient woodland, surrounded by agricultural land (Savill et al. 2010). Over the study period (1987–2010), mean annual temperature and precipitation (means are presented with 95% confidence intervals [CI], unless stated otherwise) were 10.5 [10.1, 10.9]°C and 665 [622, 708] mm, respectively (climatic data were obtained from Oxford Radcliffe Metrological Station, University of Oxford). The badger population resident at this site was not limited to the woodland (although all setts [communal burrows] were within the woodland), foraging over a total area of at least 6 km^2^, including surrounding farmland. Nevertheless, this population was geographically discrete, limiting, but not eliminating, the potential for migration into or out of the study area (Macdonald et al. 2008). Social group territory boundaries within this population have been mapped using bait marking approximately every 2 years (Kilshaw et al. 2009), defining a mean of 19 [17, 21] social groups per study year between 1987 and 2005 (Dugdale et al. 2010). While permanent dispersal between groups was low, temporary movements occurred frequently, mainly to neighboring groups (Macdonald et al. 2008).

These groups contain close kin (Dugdale et al. 2008) with a mean of 5.6 [5.2, 6.0] females and 5.8 [5.4, 6.2] males of breeding age (Dugdale et al. 2010), of which 1.9 [1.8, 2.0] were assigned parentage each year, for both sexes (Dugdale et al. 2007). Natal philopatry and high levels of relatedness between group members potentially increase the likelihood of matings between first-order relatives; however, the high rate of extra-group matings in high-density badger populations (*ca*. 50%: Carpenter et al. 2005; Dugdale et al. 2007) could reduce the frequency of inbreeding.

Badgers were captured 3–4 times per year, over 2 weeks in late May, August, and October–November, with 1 week of trapping in January in some years. Badger cubs are typically born in mid-February in a highly altricial state and remain below ground for their first 8 weeks from birth (Roper 2010). Because cubs are highly dependent on maternal care during this period, trapping was suspended on welfare grounds until they were fully weaned at around 15 weeks of age (Macdonald et al. 2009). All trapping and handling procedures were approved by the University of Oxford ethics committee and carried out under licenses (Natural England Licence 20104655 and Home Office PPL30/2835) in accord with the 1986 UK Animals (Scientific Procedures) Act and the 1992 Protection of Badgers Act.

Badgers were trapped using steel-mesh cages placed at active setts, sedated with ketamine hydrochloride (0.2 mL/kg body weight, Thornton et al. 2005), and given a unique tattoo number, in their inguinal region, for permanent individual identification. Individuals were sexed and classified as cub (animals in their first year) or adult based on their size and trapping history. Of 1410 individuals trapped from 1987 to 2010, 975 (69%) were of known age (first trapped as cubs). Intact follicles from approximately 100 plucked hairs, along with jugular blood samples (*ca*. 3 mL), were collected for genetic analyses.

### Microsatellite genotyping

We genotyped 1170 (83%) badgers, trapped between 1987 and 2010, of which 838/975 (86%) were first caught as cubs (136 cubs were not sampled, and one cub had only one sample that did not amplify). We used a minimum of 20 hair follicles or 25 *μ*L of whole blood from each individual for DNA extraction, using a slightly modified Chelex protocol (Walsh et al. 1991). We genotyped individuals using 35 fluorescently labeled autosomal microsatellite markers, grouped into seven multiplexes (4–9 markers per set; Table S1) using Multiplex Manager 1.0 (Holleley and Geerts 2009). Primer pairs were analyzed in AutoDimer 1.0 for potential cross-reactivity within and between primers (Vallone and Butler 2004). We used a 2-*μ*L Qiagen Multiplex PCR reaction (Annavi et al. 2011) and then sequenced and analyzed samples using GENEMAPPER 3.5.

Genotyping was 97% complete, with each individual genotyped for a mean of 34.0 ([33.8, 34.1]; range = 18–35) loci. No DNA remained for 14 badgers after we ran the initial set of 22 microsatellites, so these could not be genotyped for further loci; however, the 18–22 loci they were typed for were included in our analyses. A GENEPOP 4.0.10 (Raymond and Rousset 1995) analysis of 30 adults from three years, selected randomly, showed that none of the markers violated the expectations of Hardy–Weinberg equilibrium (*m* = 35, *α* = 0.05, adjusted *P* = 0.050–0.001) and no pair of loci was linked consistently, after false discovery rate control (Benjamini and Hochberg 1995). Mean allelic dropouts (*ε*_1_) and false alleles (*ε*_2_) were estimated at 0.005 using PEDANT 1.0 (Johnson and Haydon 2007), by regenotyping 5% of individuals, chosen at random. CERVUS 3.0.3 (Kalinowski et al. 2007) and MICRO-CHECKER 2.2.3 (Van Oosterhout et al. 2004) were used to estimate allelic diversity, observed heterozygosity, and null alleles for each marker (Table S1). Mean observed and expected heterozygosity were 0.45 [0.39, 0.51] and 0.49 [0.43, 0.55], respectively. The mean number of alleles was 4.46 [3.79, 5.13].

### Parentage assignment

**Figure 1:**
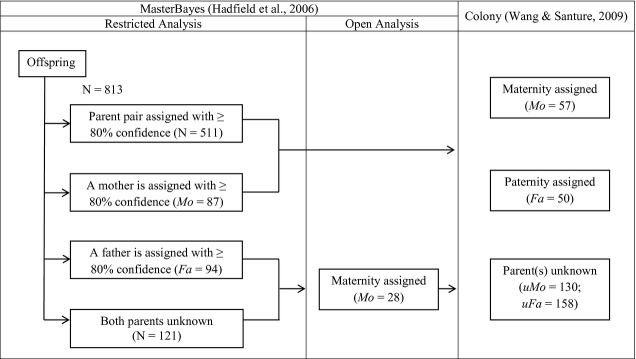
Flowchart of the parentage assignment rules used in MasterBayes 2.47 and Colony 2.0. The MasterBayes restricted analysis only included females aged ≥2 years and present in the cubs’ natal group as candidate mothers, whereas the open analysis included all females in the population aged ≥2 years. *N* = total number of cubs; *Mo* = number of cubs with an assigned mother; *Fa* = number of cubs with an assigned father; *uMo* = number of cubs with an unassigned mother; *uFa* = number of cubs with an unassigned father. Parentage was assigned with ≥80% confidence.

We conducted parentage analyses for 813 genotyped cubs (Fig. 1; trapped 1988–2010); we excluded 25 cubs trapped in the first year of the capture–mark–recapture study (1987) due to low confidence in these assignments. Bayesian parentage analysis was applied to each cub cohort, in a restricted analysis, using MasterBayes 2.47 (Hadfield et al. 2006) in R 2.12.2 (R Development Core Team 2008). All females aged ≥2 years, present in a cubs’ natal group, were included as candidate mothers; all males of breeding age (>1 year old), across the entire population, were included as candidate fathers (Dugdale et al. 2007). Where cubs were not assigned mothers (*N* = 215), we retested parentage in an open analysis, considering all extant females aged ≥2 years as potential candidate mothers. We included the geographical locations (GPS coordinates of each group's main sett) of all offspring and candidate fathers in our models to estimate the probability with which paternity assignment decreases with Euclidean distance. Unsampled males were assigned the mean Euclidean distance derived from all sampled individuals.

These parentage analyses were based on starting pedigrees generated by running 10,000 iterations, using default tuning parameters, and extracting the mode of the posterior distribution of the parents. All analyses used a specified number of unsampled candidate mothers and fathers (Table S2), estimated from capture–mark–recapture (Dugdale et al. 2007), two genotyping error rates (*ε*_1_ and *ε*_2_) of 0.005, and allele frequencies extracted from all genotypes.

In the final analyses, the maximum number of genotype mismatches tolerated was set to three. Tuning parameters were specified in the final analyses (ß = 5 restricted analysis, *ß* = 1 open analysis) to ensure that the Metropolis–Hastings acceptance rates were between 0.2 and 0.5 (Hadfield 2014). Markov chains were run separately for each year, for 1.5 million iterations, with a thinning rate of 500 and burn-in of 500,000. Successive samples from the posterior distribution had low autocorrelation (*r* < 0.02). Sib-ships were then reconstructed in COLONY 2.0 by partitioning each cub cohort (including cubs that were and were not assigned parent(s) in MasterBayes) into full- and half-sibship groups, using a maximum likelihood method (Wang and Santure 2009). Parentage was accepted with ≥ 0.8 probability in both MasterBayes and Colony. Maternity was assigned to 683 cubs (84% of genotyped cubs) and paternity to 655 (81%) cubs (Table S3); a maximum of three mismatches occurred between an assigned parent and cub (Table S4). Both parents were assigned to 561 (69%) cubs, and 67% of these trios had no mismatches (Tables S3 and S4).

### Estimating genetic diversity and inbreeding

We estimated three microsatellite-derived measures of multilocus heterozygosity (standardized heterozygosity [SH], Coltman et al. 1999; homozygosity by locus [HL], Aparicio et al. 2006; internal relatedness [IR], Amos et al. 2001) for 989 badgers in the pedigree using GENHET in R 2.12.2 (Coulon 2010). We excluded the mean *d*^2^ estimator (Coulson et al. 1998) because of difficulties with its interpretation (Hansson 2010). SH values were highly correlated with HL and IR (Spearman's rank correlation, *r* (HL and IR) = −0.96, *P* < 0.001), and all three heterozygosity estimators resulted in similar conclusions. For simplicity, we therefore present only analyses based on SH (results for HL and IR are presented in Tables S5–S8).

Pedigree Viewer 6.3 (Kinghorn 1994) was used to calculate *f* for 561 of the 813 genotyped cubs that were assigned both parents with ≥0.8 probability. We followed the approach of Szulkin et al. (2007) by restricting our dataset to 420 (52%) cubs that had at least one grandparent assigned, and to 88 (11%) with all four grandparents assigned (Table S9). This dataset restriction approach is important because an assumption when calculating *f* is that individuals with unknown parents are unrelated, and in wild pedigrees there will inevitably be missing parentage links, which could bias results. When parentage information is missing, certain inbreeding events cannot be detected (Marshall et al. 2002); thus, by restricting datasets, these biases can be minimized, although sample size is reduced.

### Data analyses

A recapture history file was compiled consisting of 24 annual trapping records (1987–2010) for 975 individuals of known age (i.e., first trapped as cubs). If an individual was trapped at least once in a particular year (years commenced with the birth of cubs in February), it was denoted as “1,” otherwise “0” if it was not caught in that year.

### Age, sex, cohort, and population size

To investigate the effects of SH and *f* on first-year survival probability, we first built a “starting model” (or “global model”) to test for age, sex, cohort, and population size effects. Cubs typically exhibit lower mean interannual survival rates than adults (Macdonald et al. 2009). Adults and cubs also exhibit year-dependent survival rates (Macdonald and Newman 2002). Our starting model was therefore based on year-dependent (t) and age-dependent (two age classes, a2- [cub <1 year old]/[adult ≥ 1 year old]) survival (Φ) and recapture probabilities (*P*; starting model = Φ (a2- t/t) *P* (a2- t/t)). Sex, cohort size, and population size were then included to test their effects on cub and adult survival probabilities. We then applied a multistep approach within a Cormack–Jolly–Seber framework, to derive a “reduced model” (B1, Table 1), using MARK 6.1 (White and Burnham 1999), which we used to investigate the effects of SH and *f* on first-year survival probability.

**Table 1:** Model selection statistics for age, sex, cohort, and population size effects on annual survival (Φ) and recapture (*P*) probabilities (*N* = 975) in a multistep procedure to obtain a reduced model. No. = model number; *k* = number of parameters; Δ = QAICc (Akaike information criterion, corrected for sample size and adjusted through quasi-likelihood) from the top model (i.e., model with lowest QAICc); *ω* = relative QAICc weight (exp[−0.5 * ΔQAICc], divided by the sum of this quantity for all considered models); a2 = two age classes (cub/adult); – = standard MARK notation between age class and the dependence of the levels of these classes; t = year; (.) = constant; * = interaction effect; C = cohort size. Models with *ω* ≥ 0.01 are presented, except for A3–4 and C2, which are presented for comparative purposes

No.	Model	*k*	QDeviance	Δ	*ω*
Age-specific models					
A1	Φ (a2-t/.) *P*(t)	47	1655.8	0.00	0.88
A2	Φ (a2-t/t) *P*(t)	67	1618.2	3.98	0.12
A3	Φ (a2-./.) *P*(t)	25	1744.7	43.81	0.00
A4	Φ (.) *P*(t)	24	1785.2	82.26	0.00
Sex-specific models					
B1	Φ (a2-t/sex) *P*(t)	48	2086.7	0.00	0.98
B2	Φ (a2-t/.) *P*(t)	47	2097.4	8.66	0.01
B3	Φ (a2-t/sex) *P*(sex*t)	71	2048.3	9.57	0.01
Cohort-size effect models					
C1	Φ (a2-t/sex) *P*(t)	48	2099.5	0.00	1.00
C2	Φ (a2-t/sex*C) *P*(t)	90	2065.8	54.44	0.00
Population size effect models					
D1	Φ (a2-t/sex + population size) *P*(t)	27	5500.5	0.00	0.57
D2	Φ (a2-t/sex) *P*(t)	26	5503.1	0.53	0.43

### Effects of SH and *f* on first-year survival probability

We used the reduced model (B1, Table 1; Φ [a2- t/sex] *P* [t]) to investigate the effects of an individual's own heterozygosity (SH_Ind_; *N* = 838), as well as the heterozygosity of their assigned mother (SH_Mat;_ *N* = 683) and father (SH_Pat_; *N* = 655), on their first-year survival probability. Models included climatic effects (standardized mean summer [May–October] and winter [November–February] temperatures, and standardized total summer rainfall [May–October]). All predictors were standardized to a mean of 0 and a standard deviation (SD) of 2 (Gelman 2008) to interpret main effects in the presence of interactions and quadratic effects when model averaging (Schielzeth 2010; Grueber et al. 2011). We included all first-order interactions between each SH measure and the climatic variables, to test for climate correlated heterozygosity effects on first-year survival probability. Quadratic SH effects were included to test for nonlinearity (Neff 2004).

To control for the effect of endoparasitic infection on first-year survival, a subset of models was run, retaining log_e_(*x* + 1) transformed coccidial (gut parasite) load (even if not significant), using a restricted dataset. The restricted dataset consisted of coccidial loads derived from fecal counts of *Eimeria melis* oocysts, from 143 genotyped cubs (*N* = 47 [1993]; 23 [1994]; 34 [1995]; 28 [1996]; and 11 [1997]) caught between May and November (standardized across months; Newman et al. 2001).

Any paternal heterozygosity effect detected could result from immigrant males producing heterozygous offspring, which then breed. Such fathers might have not only high heterozygosity, but also rare alleles that could influence survival. We therefore included a measure of rare alleles (the number of rare alleles [frequency of <5%] that an offspring's father had, divided by the number of alleles that the father was typed for) when modeling the effect of paternal heterozygosity on first-year survival probability.

Similar models, incorporating climatic variables, were used to test for effects of *f* on first-year survival probability. Badgers with *f* ≥ 0.125 were designated as inbred (“1”) and those with *f* < 0.125 as outbred (“0”). To test how the effect differed depending on our greater ability to estimate *f*, which simultaneously reduced statistical power (Marshall et al. 2002; Szulkin et al. 2007), we conducted three separate analyses, with different datasets: (1) *f*_561_ included 561 cubs that had both parents assigned with ≥0.8 probability; (2) *f*_420_ was restricted to 420 of these cubs that had at least one grandparent assigned; and (3) *f*_88_ was restricted to 88 individuals that had all four grandparents assigned.

We analyzed models with the logit link function in MARK 6.1 (White and Burnham 1999). Recapture probabilities were fixed at a predetermined value from the reduced model (B1, Table 1).

### Goodness of fit

We assessed the goodness of fit of our models using a bootstrap method (Pradel 1996), implemented in MARK 6.1 (White and Burnham 1999). We estimated the variance inflation factor (ĉ), by dividing the model deviance by the bootstrapped deviance. The “starting model” was slightly overdispersed (ĉ = 1.03; *N* = 100 replicates); we therefore adjusted the Akaike information criterion (AICc, corrected for sample size; Akaike 1973) value, through quasi-likelihood:


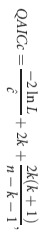


where *ĉ* = 1.03, *L* = likelihood*, k* = number of parameters, and *n* = effective sample size (Burnham and Anderson 2002; Cooch and White 2011).

### Model selection and model averaging

Information-theoretic (IT) approaches were employed to select sets of plausible models and to estimate the relative importance of each fixed effect (Burnham et al. 2011). The top model is the model with the lowest *QAICc* value (Burnham et al. 2011). If the difference in *QAICc* (Δ*QAICc*) between the top model and the model with the next lowest *QAICc* value is ≥7, the top model is considered to be the only plausible model (Burnham et al. 2011). If ΔQAICc is <7 between the top model and another model, both models are considered plausible, given these data. A model's relative Akaike weight (*ω*) is the model's relative likelihood, given these data (exp [−0.5 * ΔQAICc]), divided by the sum of the likelihoods for all models considered (whether plausible or not). The evidence ratio between two models is calculated as the ratio of *ω* for each of those two models.

We estimated two types of model-averaged parameters, using the “zero method” (averaged over all plausible models, when ΔQAICc <7; a parameter estimate (and error) of zero is substituted into those models where the parameter is absent) and the “natural average method” (averaged over plausible models in which the given parameter is present and weighted by the summed weights of these models, Burnham and Anderson 2002). Heterozygosity only has a small effect on fitness-related traits generally (reviewed by Chapman et al. 2009; Miller and Coltman 2014); therefore, we used the natural average method. We also provide the zero method estimates for comparison; however, the zero method can reduce parameter estimates (and errors) toward zero, particularly when the predictors have weak effects (Lukacs et al. 2010).

Parameter estimates of main effects were averaged over the plausible models (including models both with and without the parameter estimate as an interaction, and/or quadratic effects). The standard errors of the parameter estimates in the MARK output are conditional on a given model. Unconditional standard errors for model-averaged parameter estimates were therefore calculated using equation 4 in Burnham and Anderson (2004). The relative importance of each fixed effect was calculated as the total *ω* of all plausible models that included the fixed effect of interest.

### General effect hypothesis

We tested the extent to which heterozygosity could reflect genome-wide heterozygosity, and ultimately the level of *f* (using three datasets: *f*_561_, *f*_420_, and *f*_88_). The correlation observed between an individual's SH_Ind_ and *f* was computed using a Spearman's rank correlation; the expected correlation (*r*) between SH_Ind_ and *f* was then calculated as:


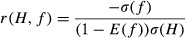


[equation 4, Slate et al. 2004;] where E(*f*) and *σ*(*f*) represent the mean and SD of *f*, and *σ*(H) represents the SD of SH_Ind_, calculated using Equation 1 in Slate et al. (2004).

Following Balloux et al. (2004), we subdivided the 35 loci, at random, into two sets (consisting of 17 and 18 loci) and tested whether the mean heterozygosity of the first set of loci was correlated with the second set, using the stats4 2.13.2 package in R 2.13.2. This procedure was repeated 100 times with different combinations of two sets of loci to calculate the heterozygosity–heterozygosity correlation (HHC). We then calculated the mean and SD of the Spearman's rank correlation coefficient. To detect identity disequilibrium (correlations in heterozygosity among loci) due to variance in inbreeding, we also calculated the parameter, *g*_*2*_ (and its standard error), using 1000 iterations in the software RMES (David et al. 2007), because this gives a more powerful statistic than HHC (Szulkin et al. 2010).

### Local effect hypothesis

To test whether HFCs were associated with single-locus local effects, we ran two types of linear models. Each model was run on the SH_Ind_, SH_Mat_, and SH_Pat_ measures separately, to test for their effects on first-year survival probability, following Szulkin et al. (2010). We used MARK 6.1 (likelihood methods produced large standard errors; therefore, for this analysis, we used MCMC with default parameters: tuning = 4000, burn-in = 1000, stored samples = 10,000) and constructed models that included: (1) all 35 single-locus SLH_Ind_, SLH_Mat_, or SLH_Pat_ measures (homozygous = 0; heterozygous = 1), and their interactions with standardized total summer rainfall [May–October] (SLH_Ind_*SR, SLH_Mat_*SR, or SLH_Pat_*SR); and (2) the multilocus SH_Ind_, SH_Mat_, or SH_Pat_ measures, and SH_Ind_*SR, SH_Mat_*SR, or SH_Pat_*SR, respectively. First-order climatic variables (standardized mean winter temperature [November–February] and standardized total summer rainfall [May–October]) were included in both models. As MARK does not allow for missing individual covariates, we replaced the missing genotypes for individuals with <35 loci genotyped with the mean value for each missing locus. This approach retains information from other loci without biasing the regression coefficients of loci with missing data (Nakagawa and Freckleton 2008; Szulkin et al. 2010). Locus *Mel-114* was excluded from these analyses, because only six cubs, three mothers, and two fathers were heterozygous at this marker. Loci *Mel-135* and *Mel-138* were also excluded due to collinearity: These had variance inflation factors of 6.97 and 7.12, respectively (Zuur et al. 2010). QAICc was used to establish whether the model including all of the single-locus effects had greater explanatory power than the multilocus model.

Unless otherwise stated, all statistical analyses were carried out in R 2.13.2 (R Development Core Team 2008).

## Results

### Age, sex, cohort, and population size effects on first-year survival probability

We found two plausible age-specific models of annual survival (Φ) and recapture probabilities (Table 1: A1, A2). Recapture probabilities were year-dependent, and cubs exhibited a lower annual survival probability (mean Φ = 0.68 [0.61, 0.75]) than adults (0.82 [0.80, 0.83]), with year dependence in the cub age class (Table 1, Figure S1). Year-independent adult survival was 7.3 times more likely than year-dependent adult survival (evidence ratio A1/A2 = 0.88/0.12, Table 1). Including sex, only one model was supported by these data, where the mean survival probability of adult females (0.84 [0.82, 0.86]) exceeded that of adult males (0.79 [0.76, 0.81]), but there was no apparent sex effect on first-year survival probability (B1, Table 1). The model incorporating a cohort-size effect on survival dynamics was not supported (C2 probability = 0%, Table 1). Although the model that included population size was listed as the top model (D1, Table 1), its CI overlapped zero (*β* = −0.002 [−0.004, 0.0004]), and the data provided only 1.3 times more support for including (D1) than excluding population size (D2, Table 1). Model B1 (Table 1) was therefore selected as our reduced model to investigate the effects of SH and *f* on first-year survival probability.

### Effects of SH and *f* on first-year survival probability

First-year survival probability correlated positively and most strongly with winter temperature (E3, Table 2), whereas the 95% CIs of the effect of summer rainfall and summer temperature overlapped zero (E1 and E2, Table 2). Although SH_Ind_, SH_Mat_, SH_Pat_, and their quadratic and interaction effects on badger first-year survival probability were components of some plausible models (Table S10), the 95% CI of these estimates overlapped zero (Table 2). These results were similar when controlling for both winter temperature (the strongest predictor, E3, Table 2) and coccidial load (Table 3).

**Table 2:** Model-averaged estimates of an individual's own (SH_Ind_), maternal (SH_Mat_), and paternal (SH_Pat_) standardized multilocus heterozygosity effects on their first-year survival probability (Φ) using natural average and zero methods (Burnham and Anderson 2002). No. = sequential numbering of each model-averaged estimate; *β* = effect size; CI = confidence interval; relative importance = sum of Akaike weights of models that contain the effect of interest; SR = total summer rainfall (May–October); Tsm = mean summer temperature (May–October); Twt = mean winter temperature (November–February); SH_Ind_^2^, SH_Mat_^2^, and SH_Pat_^2^ = quadratic effects; * = interaction effect. All predictors were standardized to a mean of 0 and a standard deviation of 2. Effect sizes where the 95% CI does not overlap zero are in bold

		Natural average method		Zero method		
				
No.	Fixed effect	*β*	95% CI	*β*	95% CI	Relative importance
SH_Ind_ models						
E1	SR	0.29	−0.15, 0.72	0.16	−0.26, 0.57	0.54
E2	Tsm	−0.06	−0.46, 0.34	−0.02	−0.18, 0.14	0.32
E3	Twt	**0.66**	**0.23, 1.09**	**0.66**	**0.22, 1.09**	**0.97**
E4	SH_Ind_	0.23	−0.16, 0.62	0.17	0.22, 0.55	0.69
E5	SH_Ind_^2^	−0.30	−0.84, 0.24	−0.08	−0.39, 0.22	0.27
E6	SH_Ind_*SR	0.52	−0.31, 1.34	0.10	−0.27, 0.46	0.18
E7	SH_Ind_*Tsm	−0.12	−0.95, 0.70	−0.01	−0.10, 0.08	0.08
E8	SH_Ind_*Twt	0.17	−0.65, 0.99	0.03	−0.18, 0.24	0.18
SH_Mat_ models						
F1	SR	0.51	−0.01, 1.02	0.43	−0.15, 1.01	0.82
F2	Tsm	−0.17	−0.62, 0.27	−0.06	−0.32, 0.20	0.36
F3	Twt	**0.56**	**0.05, 1.06**	0.47	−0.12, 1.06	0.82
F4	SH_Mat_	0.16	−0.32, 0.65	0.11	−0.29, 0.52	0.65
F5	SH_Mat_^2^	0.37	−0.41, 1.16	0.09	−0.27, 0.46	0.47
F6	SH_Mat_*SR	0.88	−0.12, 1.88	0.33	−0.62, 1.29	0.37
F7	SH_Mat_*Tsm	−0.16	−1.05, 0.74	−0.01	−0.09, 0.07	0.06
F8	SH_Mat_*Twt	0.24	−0.79, 1.28	0.04	−0.20, 0.28	0.16
SH_Pat_ models						
G1	SR	0.53	−0.02, 1.07	0.44	−0.17, 1.06	0.83
G2	Tsm	−0.34	−0.83, 0.15	−0.19	−0.67, 0.29	0.54
G3	Twt	**0.72**	**0.17, 1.28**	**0.70**	**0.11, 1.28**	**0.94**
G4	SH_Pat_	0.33	−0.15, 0.82	0.28	−0.23, 0.78	0.81
G5	SH_Pat_^2^	0.11	−0.63, 0.84	0.02	−0.18, 0.23	0.22
G6	SH_Pat_*SR	0.99	−0.04, 2.01	0.50	−0.69, 1.70	0.50
G7	SH_Pat_*Tsm	0.06	−0.98, 1.10	0.01	−0.13, 0.14	0.12
G8	SH_Pat_*Twt	0.43	−0.63, 1.49	0.12	−0.38, 0.61	0.27

**Table 3:** Model-averaged estimates of an individual's own (SH_Ind_), maternal (SH_Mat_), and paternal (SH_Pat_) standardized multilocus heterozygosity after controlling for coccidial infection (*Em* = *Eimeria melis*) on their first-year survival probability (Φ) using natural average and zero methods (Burnham and Anderson 2002). No. = sequential numbering of each model-averaged estimate; *β* = effect size; CI = confidence interval; relative importance = sum of Akaike weights of models that contain the effect of interest; SR = total summer rainfall (May–October); Twt = mean winter temperature (November–February); SH_Ind_^2^, SH_Mat_^2^, and SH_Pat_^2^ = quadratic effects; * = interaction effect. All predictors were standardized to a mean of 0 and a standard deviation of 2

		Natural average method		Zero method		
				
No.	Fixed effect	*β*	95% CI	*β*	95% CI	Relative importance
SH_Ind_ models						
H1	SH_Ind_	1.18	−0.04, 2.40	0.96	−0.10, 2.02	0.71
H2	SH_Ind_^2^	−2.27	−5.19, 0.66	−1.37	−4.46, 1.72	0.60
H3	SH_Ind_*SR	4.99	−0.03, 10.01	3.68	−0.07, 7.43	0.53
H4	SH_Ind_*Twt	−1.54	−3.45, 0.37	−0.64	−2.46, 1.17	0.42
SH_Mat_ models						
I1	SH_Mat_	1.34	−2.05, 4.73	0.96	−1.91, 3.83	0.72
I2	SH_Mat_^2^	1.34	−1.52, 4.19	0.46	−1.25, 2.18	0.35
I3	SH_Mat_*SR	4.57	−0.95, 10.09	1.18	−2.74, 5.09	0.26
I4	SH_Mat_*Twt	0.77	−0.93, 2.47	0.27	−0.78, 1.32	0.35
SH_Pat_ models						
J1	SH_Pat_	−2.23	−8.46, 3.98	−2.11	−8.49, 4.26	0.94
J2	SH_Pat_^2^	5.66	−3.97, 15.30	3.89	−5.52, 13.29	0.69
J3	SH_Pat_*SR	−4.34	−16.77, 8.10	0.07	−0.94, 1.08	0.31
J4	SH_Pat_*Twt	0.32	−3.93, 4.58	−1.34	−7.10, 4.42	0.22

**Figure 2:**
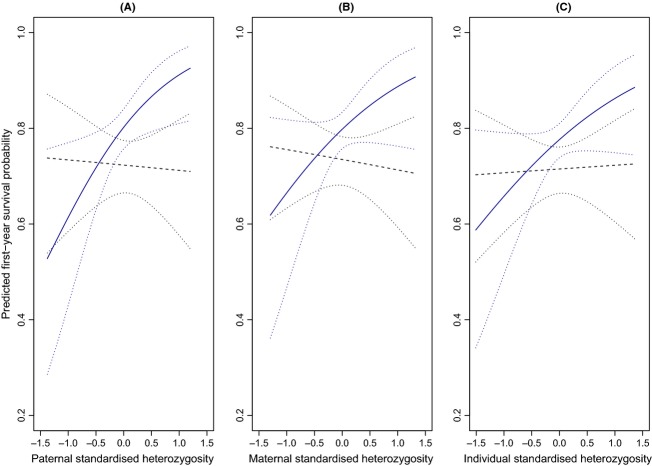
The relationship between predicted first-year survival probability and: (A) paternal standardized multilocus heterozygosity (SH_P__at_); (B) maternal heterozygosity (SH_M__at_); and (C) an individual's own heterozygosity (SH_I__nd_). Standardized total summer rainfall (SR) was categorized for ease of visualization; SR > 0 and SR ≤ 0 were years with above and equal to or below mean rainfall, respectively. Probabilities are plotted under mean conditions of high (solid line; SR = −0.4) and low (dashed line; SR = 0.6) total summer rainfall (May–October). The dotted lines represent the 95% confidence intervals. First-year survival probabilities are based on (A) Φ (SH_P__at_, Twt, SR, SH_P__at_*SR), (B) Φ (SH_I__nd_, Twt, SR, SH_I__nd_*SR), and (C) Φ (SH_M__at_, Twt, SR, SH_M__at_*SR) models where Twt = standardized mean winter [November–February] temperatures and * = interaction term.

The most supported SH_Pat_ model included a positive interaction between SH_Pat_ and total summer rainfall (SH_Pat_*SR = 1.01 [0.03, 1.99]; Table S10). This model had 2.4 times the support of the highest-ranked model without SH_Pat_*SR (evidence ratio SH_Pat_ model 1:3 = 0.12/0.05, Table S10). In years with high summer rainfall, offspring sired by males with higher levels of heterozygosity exhibited higher survival probabilities than offspring sired by males with lower levels of heterozygosity (Fig. 2A). This was a marginal effect, as the 95% CI of the model-averaged estimate of SH_Pat_*SR overlapped zero (0.99 [−0.04, 2.01]; model averaging models with ΔQAICc <2 = 0.99 [−0.01, 1.99]). SH_Pat_*SR, however, had a relative importance of 0.50 and occurred in the most supported model. Fitting natal social group as a categorical variable in all SH_Pat_ models (to account for heterozygosity differences among social groups and environmental heterogeneity within territories) produced similar results (Tables S11 and S12).

Considering SH_Mat_, there was a similar positive interaction with summer rainfall in the second most supported model (SH_Mat_*SR = 0.81[−0.12, 1.75]); however, the top model, which was 1.6 times better supported, did not include this interaction (or SH_Mat_; Table S10). The model-averaged estimates of the interactions between summer rainfall and both SH_Ind_ and SH_Mat_ did not differ from zero (Table 2; Fig. 2).

*f* was not associated with first-year survival probability, when accounting for winter temperature, using datasets assigning: both parents (*N* = 561 cubs; *β* = −0.48 [−1.55, 0.60]); at least one grandparent (*N* = 420; *β* = −0.40 [−1.47, 0.66]); or all four grandparents (*N* = 88; *β* = −1.08 [−4.20, 2.03]). Nevertheless, *f* occurred in some plausible models (ΔQAICc <7), but these models had around half the support (0.51 (*f*_561_); 0.47 (*f*_420_); 0.43 (*f*_88_)) of models without *f* (Table 4).

**Table 4:** Plausible models, and their model selection statistics, of the effect of inbreeding on first-year survival probability (Φ). Three datasets were used, including individuals for which at least both parents (*f*_*561*_), one grandparent (*f*_*420*_), or all four grandparents (*f*_*88*_) were assigned. No. = model number; *k* = number of parameters; Δ = difference in QAICc from the top model (i.e., model with lowest QAICc); *ω* = relative QAICc weight (exp[−0.5 * ΔQAICc], divided by the sum of this quantity for all considered models). T_wt_ = mean winter temperature (November–February)

No.	Model	*k*	QDeviance	Δ	*ω*
*f*_561_ models					
K1	Φ(T_wt_)	5	3421.6	0.00	0.66
K2	Φ(T_wt_, *f*_*561*_)	6	3422.9	1.34	0.34
*f*_420_ models					
L1	Φ(T_wt_)	5	2298.7	0.00	0.68
L2	Φ(T_wt_, *f*_*420*_)	6	2300.3	1.51	0.32
*f*_88_ models					
M1	Φ(T_wt_)	5	375.0	0.00	0.70
M2	Φ(T_wt_, *f*_*88*_)	6	376.6	1.67	0.30

### General effect hypothesis

Inbred badgers had a lower mean SH_Ind_ than outbred badgers, based on datasets *f*_561_ and *f*_420_, but not based on *f*_88_, probably due to the small number of inbred badgers in this dataset (*N* = 3; Table 5). The predicted correlation coefficient between SH_Ind_ and *f* (*r*(SH_Ind_*, f*)) was −0.25 (*f*_561_; vs. −0.29 for *f*_420_ and −0.18 for *f*_88_); however, the observed correlations were relatively weak (*f*_561_: *r*(SH_Ind_, *f*) = −0.16, *P* < 0.001; *f*_420_: *r*(SH_Ind_, *f*) = −0.20, *P* < 0.001; *f*_88_: *r*(SH_Ind_, *f*) = −0.02, *P* = 0.878). This is consistent with the detection of a significant, but weak, heterozygosity–heterozygosity correlation (HHC) between the two random subsets of the loci (mean HHC = 0.15, SD = 0.03, range = 0.09–0.20, *P* < 0.001). Variance in inbreeding was detected: The identity disequilibrium parameter *g*_*2*_ differed from zero (*g*_*2*_ = 0.01, SD = 0.003, *P* < 0.001).

**Table 5:** Summary statistics for datasets *f*_561_,*f*_420_, and *f*_88_ used to calculate the inbreeding coefficient (*f*). CI = confidence interval; SH_Ind_ = individual's own standardized heterozygosity; *N* = number of individuals. ¥ = Datasets where SH_Ind_ of inbred individuals is significantly different to SH_Ind_ of outbred individuals are in bold

			Inbred (*f* ≥ 0.125)		Outbred (*f* < 0.125)		
					
Dataset	Mean *f* [95% CI]	SH_Ind_ [95% CI)	*N*	Mean SH_Ind_ [95% CI]	*N*	Mean SH_Ind_ [95% CI]	Mann–Whitney test: SH_Ind_ of inbred vs. outbred cubs
*f*_561_	0.010 [0.006,0.014]	0.99 [0.97,1.01]	25	0.77 [0.68,0.85]	536	1.01 [0.99,1.02]	^**¥**^**U = 2590,** ***P*** **<** **0.001**
*f*_420_	0.014 [0.009,0.019]	1.00 [0.98,1.02]	25	0.77 [0.69,0.85]	395	1.01 [0.99,1.03]	^**¥**^**U = 8026,** ***P*** **<** **0.001**
*f*_88_	0.010 [0.002,0.018]	1.04 [1.00,1.08]	3	0.93 [0.62,1.79]	85	1.05 [1.00,1.09]	U = 157.5, *P* < 0.490

### Local effect hypothesis

The multilocus SH_Ind_, SH_Mat_, and SH_Pat_ models had complete support (i.e., 100%) compared to single-locus SLH_Ind_, SLH_Mat_, and SLH_Pat_ models, respectively (Table 6; Table S13). The interactive effect between summer rainfall and multilocus SH_Pat_ was significant (Table S13).

**Table 6:** Model selection statistics for multilocus (individual's own [SH_Ind_], maternal [SH_Mat_], and paternal [SH_Pat_]) and single-locus (individual's own [SLH_Ind_], maternal [SLH_Mat_], and paternal [SLH_Pat_]) standardized heterozygosity effects on first-year survival probability. QAICc = Akaike information criterion, corrected for sample size and adjusted through quasi-likelihood; Δ = difference in QAICc from the top model (i.e., model with lowest QAICc); *ω* = relative QAICc weight (exp[−0.5 * ΔQAICc], divided by the sum of this quantity for all considered models)

Model	−2log Likelihood	*k*	*N*	QAICc	Δ	*ω*
Individuals’ own						
SH_Ind_	5223.14	7	2777	5085.05	0.00	1.00
SLH_Ind_	5152.56	69	2777	5144.05	59.00	0.00
Maternal						
SH_Mat_	4358.86	7	2356	4245.95	0.00	1.00
SLH_Mat_	4290.46	69	2356	4307.72	61.77	0.00
Paternal						
SH_Pat_	4008.84	7	2189	3906.13	0.00	1.00
SLH_Pat_	3892.40	69	2189	3921.58	15.46	0.00

## Discussion

Badger cubs with heterozygous fathers (but not mothers) exhibited higher first-year survival probability than cubs with less heterozygous fathers in the top model, but only in years with higher summer rainfall. This effect had marginal support when model averaging as the confidence interval of the estimate overlapped zero slightly. The effects of genetic diversity on fitness-related traits have been reported to be more detectable under advantageous conditions (Harrison et al. 2011). In the British Isles, badgers feed predominantly on earthworms (*Lumbricus terrestris*) that are only available at the soil surface under specific conditions, that is, when the soil is moist (Macdonald et al. 2010). Low rainfall can thus reduce earthworm availability, impacting on first-year survival probability (Macdonald and Newman 2002; Macdonald et al. 2010; Nouvellet et al. 2013). Because the contribution of paternal heterozygote advantage to first-year survival probability was not apparent under stressful (drier) climatic conditions, conditions that elevate indiscriminate mortality (Macdonald et al. 2010; Nouvellet et al. 2013) might mask this differential effect.

How the paternal heterozygosity contributes to offspring fitness (i.e., first-year survival probability) is, however, unclear, because paternal care is negligible in badgers (Dugdale et al. 2010). A potential mechanism would be mothers invest in their offspring differentially (Burley 1986) according to the heterozygosity of the offspring's father, where this translates into survival differences in good years. This would be dependent upon badgers being able to detect the heterozygosity of individual conspecifics, or traits linked with this. This could be possible, given that badgers have a highly developed olfactory communication system and produce a subcaudal scent, which encodes individual-specific information (Buesching et al. 2002; Sin et al. 2012). Alternatively, females may make cryptic choices according to heterozygosity (Løvlie et al. 2013), for example, through selective embryo implantation or absorption (Yamaguchi et al. 2006).

Cryptic population structure can also produce spurious HFCs (Slate and Pemberton 2006). It is conceivable that fathers with higher heterozygosity might live in higher-quality territories (Woodroffe and Macdonald 2000); thus, their offspring would have a better chance of surviving over their first year, although this is countermanded somewhat by around half of offspring being sired by extra-group males. Badgers are more related within groups (*R* = 0.198 ± 0.039) compared with the neighboring groups (*R* = 0.088 ± 0.027) in the Wytham population (Dugdale et al. 2008), but fitting natal social group to our SH_Pat_ models to account for the greatest spatial clustering of relatives did not alter our conclusions (Tables S11 and S12).

We found no evidence for interactions between climate variables and SH_Ind_ or SH_Mat_ on first-year survival probability. Deleterious recessive alleles, causing inbreeding depression on survival, could have been purged before we were first able to trap postemergence cubs that survived to at least 15 weeks. Preemergence mortality has been inferred in this study population (36%, extrapolated from ultrasound, Macdonald and Newman 2002; mean fetal (1.9 [1.8, 2.0]) versus postemergence (1.4 [1.3, 1.5]) litter size, Dugdale et al. 2007). This missing fraction, which may be linked to inbreeding, limited our power to detect any correlation between first-year survival probability and SH_Ind_. Nevertheless, these limitations simultaneously afford us a level of minimal confidence in these data – where paternal heterozygosity effects were observed despite reduced statistical power.

### General, local, and direct effects

The marginal paternal survival–heterozygosity correlation in years with high summer rainfall was not due to rare paternal alleles, but was mainly due to genome-wide effects, that is, inbreeding depression (Table 6); the multilocus SH_Pat_ model was better supported than the single-locus SLH_Pat_ model. Multilocus SH_Ind_ and SH_Mat_ were also better supported than the single-locus SLH_Ind_ and SLH_Mat_ models, respectively.

Theory predicts that HFCs should be weak, or undetectable, in populations where variance in inbreeding is low (Balloux et al. 2004). Variance of *f* was 0.002, HHC was positive, and although the *g*_*2*_ value was small (0.01, SD = 0.003), it differed from zero, consistent with the occurrence of identity disequilibrium in the population (Balloux et al. 2004; David et al. 2007). A meta-analysis of identity disequilibrium in HFC studies by Miller and Coltman (2014) estimated a mean *g*_*2*_ of 0.007 (SD = 0.022, *N* = 129) or 0.025 (SD = 0.031, *N* = 26) using studies with *g*_*2*_ estimates that differed from zero. In this meta-analysis, the *g*_*2*_ value increased with effect size; thus, our estimate of *g*_*2*_ lies in the lower half of published values, and correspondingly, we report a weak HFC. Miller and Coltman (2014) also reported that *g*_*2*_ did not differ with the number of loci used (average = 19 excluding an outlier); however, they recommend that 5611 markers are required to assess HFCs. Although we used 35 markers, more than average, low power could have affected our conclusions; in particular, weak correlations in the multilocus model would hinder detection of local effects (Szulkin et al. 2010).

Although our markers provided information on inbreeding, the observed correlation between *f* and SH_Ind_ ranged from −0.02 to −0.20, consistent with HFCs typically being weak (e.g., Chapman et al. 2009; weighted mean effect size (*r*) = 0.09 [0.07, 0.11]). Deviation between realized (i.e., the actual proportion of the genome that is “identical by descent”, IBD) and pedigree-estimated *f*, due to chance events during Mendelian segregation, may weaken the correlation between SH_Ind_ and pedigree-based *f*, compared to realized *f* (Forstmeier et al. 2012). Furthermore, first-year survival probability was not associated with the coefficient of inbreeding (*f*); however, *f* was a component of some plausible models, and small sample sizes potentially limited our power to detect this effect. Inbreeding levels can be underestimated when pedigree information is incomplete (i.e., inbred individuals will be assigned incorrectly as outbred, if their ancestors are unassigned). Consequently, this will underestimate the severity of, or fail to detect, inbreeding depression (Keller et al. 2002; Walling et al. 2011) or even generate inbreeding depression erroneously if there is systematic bias in the inbred individuals that are assigned as outbred with respect to fitness (e.g., if longer-lived individuals are less likely to have their grandparents assigned, they are therefore more likely to be assigned as outbred).

### Age- and sex- specific survival probabilities

Annual survival probabilities vary in ways that affect badger population demographics (Macdonald et al. 2009). We found that cubs survived less well than did adults (corroborating Macdonald and Newman 2002; Macdonald et al. 2009). First-year survival probability was not affected by sex, population size, or cohort size, but varied considerably between years.

Adult males exhibited significantly lower annual survival probabilities than did adult females, which might be explained by the mitochondrial theory of aging (Loeb et al. 2005). Male badgers have a faster rate of reproductive senescence than females (Dugdale et al. 2011). The mitochondria of male rats produce twice as much hydrogen peroxide as female rats; hydrogen peroxide induces oxidative stress, damaging, and aging cells, which reduces male, relative to female, longevity (Vina et al. 2003).

## Conclusion

It is important to consider the potential mechanisms that drive environmental or measure specific HFCs. Studies that only investigate HFCs over a narrow range of environmental conditions could miss important effects that are manifested only under advantageous conditions (when there is enough variation in fitness; Harrison et al. 2011, this study) or adverse conditions (due to increased magnitude of inbreeding depression, Lesbarreres et al. 2005; Da Silva et al. 2006; Brouwer et al. 2007; Fox and Reed 2011). Additionally, only investigating HFCs using direct individual measures might lead to HFCs being missed completely, when they are due to parental genetic effects (Brouwer et al. 2007; this study).

## Data Accessibility

Data are available from the Dryad Digital Repository: http://doi.org/10.5061/dryad.2kj82.

## Supporting Information

**Table S1** Characterisation of the 35 European badger (*Meles meles*) microsatellite loci used. *N* = the total number of individuals that were genotyped, bp = base pairs, *H*_O_ = observed heterozygosity, *H*_E_ = expected heterozygosity. **Table S2** The number of unsampled candidate mothers (N_cm_) and fathers (Ncf) specified in the Restricted and Open parentage analyses using MasterBayes 2.47 (Hadfield et al. 2006). **Table S3** Summary statistics of the genetic pedigree, generated using the pedStatSummary() function in Pedantics 1.02 (Morrissey et al. 2007). **Table S4** Number of mismatches (N) observed between parents assigned in MasterBayes 2.47 (Hadfield et al. 2006) and Colony 2.0 (Wang and Santure, 2009), and their offspring. Trio = mother–father–offspring. **Table S5** Model averaged estimates of an individual's own (HL_Ind_), maternal (HL_Mat_) and paternal (HL_Pat_) homozygosity by locus effects on their first-year survival probability (Φ) using natural average and zero methods (Burnham and Anderson 2002). **Table S6** Plausible models, and their model selection statistics, of the effect of an individual's own (HL_Ind_), maternal (HL_Mat_) and paternal (HL_Pat_) homozygosity by locus on their first-year survival probability. **Table S7** Model averaged estimates of an individual's own (IR_Ind_), maternal (IR_Mat_) and paternal (IR_Pat_) internal relatedness effects on their first-year survival probability (Φ) using natural average and zero methods (Burnham and Anderson 2002). **Table S8** Plausible models, and their model selection statistics, of the effect of an individual's own (IR_Ind_), maternal (IR_Mat_) and paternal (IR_Pat_) internal relatedness on their first-year survival probability. **Table S9** Number of offspring in the datasets depending on number of known parents and grandparents, grouped by their coefficient of inbreeding (*f*). **Table S10** Plausible models, and their model selection statistics, of the effect of an individual's own (SH_Ind_), maternal (SH_Mat_) and paternal (SH_Pat_) standardised heterozygosity on their first-year survival probability. **Table S11** Plausible models, and their model selection statistics, of the effect of paternal (SH_Pat_) standardised heterozygosity on their first-year survival probability, controlling for repeated measures within social groups. **Table S12** Model averaged estimates of paternal standardised heterozygosity (SH_Pat_) effects on their first-year survival probability (Φ), controlling for repeated measures within social groups, using natural average and zero methods (Burnham and Anderson 2002). **Table S13** The estimated effect sizes (with 95% credible intervals, from MCMC) of A) individual (SH_Ind_), maternal (SH_Mat_) and paternal (SH_Pat_) multilocus heterozygosity, and B) individual (SLH_Ind_), maternal (SLH_Mat_) and paternal (SLH_Pat_) single-locus heterozygosity on the probability of offspring surviving to age one and their interaction (*) with SR (total summer rainfall [May–October]). Estimates whose 95% confidence interval do not overlap zero are shown in bold. **Table S14** The interaction effect of paternal heterozygosity (SH_Pat_) with rainfall on first-year survival probability (Φ) with and without a measure of an individual's father's rare alleles as a covariate. **Figure S1** Estimated first-year survival probabilities (± SE) for cub cohorts from 1988 to 2009 (*N* = 975). Survival estimates were derived by model averaging.
