# Supplementary material for: Heterozygosity–fitness correlations in a wild mammal population: accounting for parental and environmental effects
Source: Ecol Evol. 2014 May 27;4(12):2594–609. doi: 10.1002/ece3.1112 (PMC4203301; doi:10.1002/ece3.1112)
Supplement: Supplementary file 1 [file ece30004-2594-sd1.pdf]

Heterozygosity–fitness correlations in a wild mammal population: accounting for parental and environmental effects

Geetha Annavi[*§†], Christopher Newman[*], Christina D. Buesching[*], David W. Macdonald[*], Terry Burke[§], and Hannah L. Dugdale[§‡¶]

[*]*Wildlife Conservation Research Unit, Department of Zoology, Recanati-Kaplan Centre, University of Oxford, Tubney House, Abingdon Road, Tubney, Abingdon, Oxfordshire OX13 5QL, UK*

[§]*NERC Biomolecular Analysis Facility, Department of Animal and Plant Sciences, University of Sheffield, Sheffield, S10 2TN, UK*

[†]*Faculty of Science, Department of Biology, University of Putra Malaysia, UPM 43400, Serdang, Selangor, Malaysia*

[‡]*Theoretical Biology, Centre for Ecological and Evolutionary Studies, University of Groningen, PO Box 11103, 9700 CC Groningen, The Netherlands*

[¶]*Behavioural Ecology and Self-Organization, Centre for Ecological and Evolutionary Studies, University of Groningen, PO Box 11103, 9700 CC Groningen, The Netherlands*

Corresponding author: David Macdonald, Wildlife Conservation Research Unit, Department of Zoology, Recanati-Kaplan Centre, University of Oxford, Tubney House, Abingdon Road, Tubney, Abingdon, Oxfordshire OX13 5QL, UK; Fax: +44 (0)1865 611 101; Email: david.macdonald@zoo.ox.ac.uk

This supplementary information contains 14 supplementary tables and one figure

Table S1 Characterisation of the 35 European badger (*Meles meles*) microsatellite loci used. N = the total number of individuals that were genotyped, bp = base pairs, H_O = observed heterozygosity, H_E = expected heterozygosity.

Locus	EMBL	N	No. of alleles	Expected allele size (bp)	Observed allele size range (bp)	*H_O	*H_E	¶Estimated null allele frequency	Multiplex Set	^Reference source
Mel-1	AH009955	1151	3	262–274	280–286	0.09	0.09	0.00	3	1
Mel-4	AF300711	1134	3	141–147	143–147	0.38	0.43	0.05	6	1
Mel-10	AJ309849	1163	2	154	160–162	0.22	0.26	0.09	1	2
Mel-12	AJ309053	1121	4	153	153–159	0.56	0.64	0.07	3	2
Mel-14	AJ309055	1144	4	188	186–196	0.67	0.72	0.04	3	2
Mel-15	AJ309056	1126	8	270	244–276	0.70	0.74	0.02	3	2
Mel-101	AJ293349	1113	4	114	122–136	0.34	0.35	0.00	1	3
Mel-102	AJ293353	1166	4	187	193–199	0.59	0.61	0.01	2	3
Mel-103	AJ293356	1165	5	249	251–263	0.65	0.66	0.01	1	3
Mel-104	AJ293352	1163	7	306	314–328	0.68	0.72	0.03	1	3
Mel-105	AJ293350	1113	9	129	133–151	0.65	0.69	0.03	1	3
Mel-106	AJ293355	1166	4	211	221–227	0.31	0.40	0.14	1	3
Mel-107	AJ293359	1158	5	280	284–292	0.51	0.59	0.07	1	3
Mel-108	AJ293354	1163	2	313	322–326	0.24	0.26	0.03	1	3
Mel-109	AJ293357	1161	5	122	106–128	0.56	0.60	0.03	1	3
Mel-110	AJ293360	1119	4	326	324–330	0.56	0.62	0.04	3	3
Mel-111	AJ230692	1162	2	126	132–138	0.19	0.18	-0.01	2	3
Mel-112	AJ230700	1139	4	414	418–430	0.66	0.72	0.04	2	3
Mel-113	AJ230713	1166	5	120	118–130	0.33	0.38	0.08	2	3
Mel-114	AJ230695	1142	2	222	232–234	0.01	0.01	0.00	2	3
Mel-115	AJ230703	1154	11	342	330–350	0.54	0.59	0.05	2	3
Mel-116	AJ293351	1156	8	107	113–135	0.63	0.69	0.05	2	3
Mel-117	AJ293358	1156	4	184	176–194	0.57	0.59	0.01	2	3
Mel-126	AJ293370	1144	4	158	164–170	0.23	0.25	0.04	4	3

Mel-127	AJ293368	1141	4	184	288–300	0.48	0.48	0.00	5	3
Mel-129	AJ293366	1148	5	213	205–219	0.61	0.62	0.00	5	3
Mel-131	AJ293367	1143	3	116	116–124	0.52	0.54	0.02	7	3
Mel-135	AJ293375	1132	4	131	237–247	0.37	0.41	0.05	6	3
Mel-137	AJ293372	1154	4	119	113–121	0.49	0.50	0.01	4	3
Mel-138	AJ293373	1104	5	495	489–503	0.37	0.41	0.05	7	3
Mel-140	AJ293374	1125	4	229	223–231	0.58	0.62	0.04	4	4
Mel-153	AJ230722	1105	2	309	309–313	0.40	0.43	0.04	6	5
Mel-161	AJ293385	1070	3	361	355–359	0.51	0.61	0.09	5	5
Mel-186	FR745448	923	5	439	431–439	0.39	0.43	0.06	4	5
Mel-191	FR745453	1126	4	273	259–275	0.25	0.27	0.05	7	5

* The observed heterozygosity and expected heterozygosity were estimated using CERVUS 3.0.3 (Kalinowski et al. 2007)
¶ Null allele frequency for each locus was calculated using MICRO-CHECKER 2.2.3 (Van Oosterhout et al. 2004).
^ Reference sources: 1 = Bijlsma et al. (2000); 2 = Domingo-Roura et al. (2003); 3 = Carpenter et al. (2003); 4 = Huck et al. (2008); 5 = Annavi et al. (2011).

Table S2 The number of unsampled candidate mothers (N_{cm}) and fathers (N_{cf}) specified in the Restricted and Open parentage analyses using MasterBayes 2.47 (Hadfield et al. 2006).

Cub cohort	Restricted analysis		Open analysis	
	N_{cm}	N_{cf}	N_{cm}	N_{cf}
1988	1	2	6	2
1989	1	4	10	4
1990	1	13	9	13
1991	1	15	10	15
1992	1	15	8	15
1993	1	8	10	8
1994	1	8	12	8
1995	1	12	12	12
1996	1	15	12	15
1997	1	16	12	16
1998	1	12	21	12
1999	1	21	23	21
2000	1	31	25	31
2001	1	28	28	28
2002	1	25	25	25
2003	1	32	25	32
2004	1	22	17	22
2005	1	16	10	16
2006	1	8	14	8
2007	1	15	28	15
2008	1	26	36	26
2009	1	33	33	33
2010	1	24	15	24

Table S3 Summary statistics of the genetic pedigree, generated using the pedStatSummary() function in Pedantics 1.02 (Morrissey et al. 2007).

**Parameter**	**Value**
Number of records	989
Maternities	683
Paternities	655
Full-sibs	327
Maternal sibs	1265
Maternal half sibs	938
Paternal sibs	1652
Paternal half sibs	1325
Maternal grandmothers	277
Maternal grandfathers	255
Paternal grandmothers	370
Paternal grandfathers	317
Maximum pedigree depth	7
Founders	212
Mean maternal sibsip size	3.0
Mean paternal sibsip size	3.3
Non-zero F	42
F > 0.125	15
Mean pairwise relatedness	0.007
Pairwise relatedness>=0.125	0.023
Pairwise relatedness>=0.25	0.012
Pairwise relatedness>=0.5	0.004

Table S4 Number of mismatches (N) observed between parents assigned in MasterBayes 2.47 (Hadfield et al. 2006) and Colony 2.0 (Wang and Santure, 2009), and their offspring. Trio = mother–father–offspring.

**Mismatches (N)**	**Assigned mother-cub**	**Assigned father-cub**	**Trio**
0	578	573	375
1	92	65	123
2	13	15	46
3	0	2	17
**Total**	**683**	**655**	**561**

Table S5 Model averaged estimates of an individual's own (HL_{Ind}), maternal (HL_{Mat}) and paternal (HL_{Pat}) homozygosity by locus effects on their first-year survival probability (Φ) using natural average and zero methods (Burnham and Anderson 2002). Effect sizes where the 95% confidence interval (CI) does not overlap zero are in bold. No. = sequential numbering of each model averaged estimate; Relative importance = sum of Akaike weights of models that contain the effect of interest; SR = total summer rainfall (May–October); Tsm = mean summer temperature (May–October); Twt = mean winter temperature (November–February); HL_{Ind}^2, HL_{Mat}^2 and HL_{Pat}^2 = quadratic effects; * = interaction effect. All predictors were standardised to a mean of 0 and a standard deviation of 2.

No.	Fixed effect	Natural average method		Zero method		
		Estimate	95% CI	Estimate	95% CI	Relative importance
HL_{Ind} models						
1	SR	0.28	-0.14, 0.71	0.16	-0.25, 0.57	0.54
2	Tsm	-0.06	-0.45, 0.33	-0.02	-0.19, 0.14	0.34
3	Twt	**0.65**	**0.23, 1.08**	**0.65**	**0.22, 1.08**	**0.97**
4	HL_{Ind}	-0.15	-0.53, 0.24	-0.09	-0.42, 0.23	0.63
5	HL_{Ind}^2	-0.26	-0.77, 0.24	-0.06	-0.31, 0.18	0.23
6	HL_{Ind}*SR	-0.56	-1.36, 0.24	-0.11	-0.50, 0.29	0.18
7	HL_{Ind}*Tsm	0.41	-0.35, 1.17	0.04	-0.13, 0.20	0.09
8	HL_{Ind}*Twt	-0.17	-0.97, 0.64	-0.03	-0.23, 0.17	0.18
HL_{Mat} models						
9	SR	0.51	-0.01, 1,03	0.40	-0.20, 1.01	0.78
10	Tsm	-0.17	-0.61, 0.27	-0.07	-0.34, 0.21	0.39
11	Twt	**0.57**	**0.05, 1.08**	0.49	-0.11, 1.08	0.84
12	HL_{Mat}	-0.16	-0.66, 0.35	-0.11	-0.54, 0.32	0.70
13	HL_{Mat}^2	0.59	-0.20, 1.37	0.22	-0.45, 0.90	0.37
14	HL_{Mat}*SR	-0.75	-1.80, 0.30	-0.22	-0.97, 0.52	0.29
15	HL_{Mat}*Tsm	0.38	-0.54, 1.30	0.04	-0.14, 0.21	0.09
16	HL_{Mat}*Twt	-0.38	-1.45, 0.69	-0.08	-0.44, 0.29	0.20

HL_{Pat} models						
17	SR	0.50	-0.04, 1.05	0.39	-0.23, 1.01	0.80
18	Tsm	-0.36	-0.84, 0.13	-0.19	-0.68, 0.30	0.55
19	Twt	**0.70**	**0.15, 1.25**	**0.66**	**0.06, 1.25**	**0.94**
20	HL_{Pat}	-0.18	-0.67, 0.30	-0.13	-0.56, 0.29	0.70
21	HL_{Pat}^2	0.06	-0.65, 0.77	0.01	-0.14, 0.16	0.18
22	HL_{Pat}*SR	-0.95	-1.93, 0.04	-0.41	-1.50, 0.67	0.43
23	HL_{Pat}*Tsm	-0.10	-1.12, 0.91	-0.01	-0.14, 0.12	0.10
24	HL_{Pat}*Twt	-0.04	-1.11, 1.02	-0.01	-0.22, 0.20	0.18

Table S6 Plausible models, and their model selection statistics, of the effect of an individual's own (HL_{Ind}), maternal (HL_{Mat}) and paternal (HL_{Pat}) homozygosity by locus on their first-year survival probability. No. = model number; Tsm = mean summer temperature (May–October); Twt = mean winter temperature (November–February); SR = total summer rainfall (May–October); HL_{Ind}^2, HL_{Mat}^2 and HL_{Pat}^2 = quadratic effect; * = interaction; k = number of parameters; QAICc = Akaike's information criterion, corrected for sample size and adjusted through quasi-likelihood; ΔQAICc = difference in QAICc from the top model (i.e., model with lowest QAICc); ω = relative QAICc weight (exp[-0.5 * ΔQAICc], divided by the sum of this quantity for all considered models, whether plausible or not); Φ = first-year survival probability. All plausible models (ΔQAICc < 7) are presented. Effect sizes (standard error) where the 95% confidence interval does not overlap zero are in bold. All predictors were standardised to a mean of 0 and a standard deviation of 2.

**No.**	**Model**	**Tsm (SE)**	**Twt (SE)**	**SR (SE)**	**SH (SE)**	**SH_{Ind}^2 (SE)**	**SH_{Ind}* Tsm (SE)**	**SH_{Ind}* Twt (SE)**	**SH_{Ind}* SR (SE)**	**k**	**Q Deviance**	**QAICc**	**Δ QAICc**	***ω***
**<u>HL_{Ind} Models</u>**														
**1**	Φ (Twt)		**0.69 (0.21)**							4	5075.4	5083.4	0.00	0.13
**2**	Φ (Twt,SR)		**0.61 (0.22)**	0.28 (0.21)						5	5073.7	5083.7	0.32	0.11
**3**	Φ (HL_{Ind},Twt)		**0.70 (0.21)**		-0.14 (0.19)					5	5074.8	5084.9	1.48	0.06
**4**	Φ (HL_{Ind},Twt,SR,HL_{Ind}*SR)		**0.61 (0.22)**	0.29 (0.22)	-0.18 (0.20)				-0.59 (0.41)	7	5071.1	5085.1	1.75	0.06
**5**	Φ (Tsm,Twt)		**0.61 (0.22)**	0.28 (0.21)	-0.14 (0.20)					5	5075.2	5085.2	1.80	0.05
**6**	Φ (HL_{Ind},Twt,SR)	-0.09 (0.19)	**0.70 (0.21)**							6	5073.2	5085.2	1.80	0.05
**7**	Φ (Tsm,Twt,SR)	-0.03 (0.20)	**0.61 (0.22)**	0.27 (0.22)						6	5073.7	5085.7	2.30	0.04
**8**	Φ (HL_{Ind},HL_{Ind}^2,Twt)		**0.71 (0.21)**		-0.11 (0.19)	-0.28 (0.26)				6	5073.8	5085.8	2.40	0.04
**9**	Φ (HL_{Ind},HL_{Ind}^2,Twt,SR)		**0.62**	0.28	-0.11	-0.28				7	5072.1	5086.1	2.71	0.03

			**(0.22)**	(0.21)	(0.19)	(0.26)								
**10**	$\Phi\ (HL_{Ind},HL_{Ind}^2,Twt,SR,HL_{Ind}*SR)$		**0.62**<br>**(0.22)**	0.29<br>(0.22)	-0.14<br>(0.19)	-0.25<br>(0.26)			-0.54<br>(0.39)	8	5070.2	5086.3	2.88	0.03
**11**	$\Phi\ (HL_{Ind},Twt,HL_{Ind}*Twt)$		**0.70**<br>**(0.21)**		-0.17<br>(0.20)			-0.23<br>(0.40)		6	5074.5	5086.6	3.17	0.03
**12**	$\Phi\ (HL_{Ind},Tsm,Twt)$	-0.09<br>(0.19)	**0.70**<br>**(0.21)**		-0.14<br>(0.19)					6	5074.6	5086.7	3.28	0.03
**13**	$\Phi\ (HL_{Ind},Twt,SR,HL_{Ind}*Twt)$		**0.62**<br>**(0.22)**	0.28<br>(0.21)	-0.17<br>(0.20)			-0.23<br>(0.40)		7	5072.9	5086.9	3.51	0.02
**14**	$\Phi\ (HL_{Ind},Tsm,Twt,SR,HL_{Ind}*SR)$	-0.03<br>(0.20)	**0.61**<br>**(0.22)**	0.29<br>(0.22)	-0.18<br>(0.20)				-0.59<br>(0.41)	8	5071.1	5087.1	3.74	0.02
**15**	$\Phi\ (HL_{Ind},Twt,SR,HL_{Ind}*Twt,HL_{Ind}*SR)$		**0.61**<br>**(0.22)**	0.29<br>(0.22)	-0.18<br>(0.20)			-0.05<br>(0.43)	-0.57<br>(0.43)	8	5071.1	5087.1	3.75	0.02
**16**	$\Phi\ (HL_{Ind},Tsm,Twt,SR)$	-0.03<br>(0.20)	**0.61**<br>**(0.22)**	0.27<br>(0.22)	-0.14<br>(0.19)					7	5073.1	5087.2	3.79	0.02
**17**	$\Phi\ (HL_{Ind},Tsm,Twt,HL_{Ind}*Tsm)$	-0.11<br>(0.19)	**0.71**<br>**(0.21)**		-0.14<br>(0.19)		0.44<br>(0.38)			7	5073.3	5087.4	3.98	0.02
**18**	$\Phi\ (HL_{Ind},HL_{Ind}^2,Twt,HL_{Ind}*Twt)$		**0.71**<br>**(0.21)**		-0.13<br>(0.19)	-0.26<br>(0.26)		-0.18<br>(0.39)		7	5073.6	5087.6	4.21	0.02
**19**	$\Phi\ (HL_{Ind},HL_{Ind}^2,Tsm,Twt)$	-0.08<br>(0.19)	**0.71**<br>**(0.21)**		-0.11<br>(0.19)	-0.27<br>(0.26)				7	5073.6	5087.6	4.25	0.02
**20**	$\Phi\ (HL_{Ind},Tsm,Twt,SR,HL_{Ind}*Tsm)$	-0.05<br>(0.20)	**0.62**<br>**(0.22)**	0.27<br>(0.22)	-0.14<br>(0.20)		0.45<br>(0.39)			8	5071.8	5087.9	4.50	0.01
**21**	$\Phi\ (HL_{Ind},HL_{Ind}^2,Twt,SR,HL_{Ind}*Twt)$		**0.63**<br>**(0.22)**	0.28<br>(0.21)	-0.13<br>(0.19)	-0.27<br>(0.26)		-0.17<br>(0.38)		8	5071.9	5087.9	4.52	0.01
**22**	$\Phi\ (HL_{Ind},HL_{Ind}^2,Tsm,Twt,SR)$	-0.02<br>(0.20)	**0.63**<br>**(0.22)**	0.28<br>(0.22)	-0.11<br>(0.19)	-0.28<br>(0.26)				8	5072.0	5088.1	4.70	0.01
**23**	$\Phi\ (HL_{Ind},HL_{Ind}^2,Tsm,Twt,SR,HL_{Ind}*SR)$	-0.02<br>(0.20)	**0.63**<br>**(0.22)**	0.29<br>(0.22)	-0.14<br>(0.19)	-0.25<br>(0.26)			-0.54<br>(0.39)	9	5070.2	5088.3	4.88	0.01
**24**	$\Phi\ (HL_{Ind},HL_{Ind}^2,Twt,SR,HL_{Ind}*Twt,HL_{Ind}*SR)$		**0.62**<br>**(0.22)**	0.29<br>(0.22)	-0.14<br>(0.20)	-0.25<br>(0.26)		-0.02<br>(0.41)	-0.53<br>(0.41)	9	5070.2	5088.3	4.89	0.01
**25**	$\Phi\ (HL_{Ind},Tsm,Twt,SR,HL_{Ind}*Tsm,HL_{Ind}*SR)$	-0.05<br>(0.20)	**0.61**<br>**(0.22)**	0.28<br>(0.22)	-0.17<br>(0.20)		0.37<br>(0.39)		-0.53<br>(0.41)	9	5070.2	5088.3	4.90	0.01
**26**	$\Phi\ (HL_{Ind},Tsm,Twt,HL_{Ind}*Twt)$	-0.09<br>(0.19)	**0.71**<br>**(0.21)**		-0.17<br>(0.20)			-0.23<br>(0.40)		7	5074.3	5088.4	4.97	0.01
**27**	$\Phi\ (HL_{Ind},HL_{Ind}^2,Tsm,Twt,HL_{Ind}*Tsm)$	-0.10<br>(0.19)	**0.72**<br>**(0.21)**		-0.12<br>(0.19)	-0.23<br>(0.26)	0.38<br>(0.37)			8	5072.6	5088.6	5.24	0.01

**28**	Φ (HL_{Ind},Tsm,Twt,SR,HL_{Ind}*Twt)	-0.04 (0.20)	**0.62 (0.22)**	0.27 (0.22)	-0.17 (0.20)			-0.23 (0.40)		8	5072.8	5088.9	5.48	0.01
**29**	Φ (HL_{Ind},Tsm,Twt,HL_{Ind}*Tsm,HL_{Ind}*Twt)	-0.11 (0.19)	**0.71 (0.21)**		-0.17 (0.20)		0.45 (0.38)	-0.26 (0.40)		8	5072.9	5089.0	5.61	0.01
**30**	Φ (HL_{Ind},HL_{Ind}^2,Tsm,Twt,SR,HL_{Ind}*Tsm)	-0.04 (0.20)	**0.63 (0.22)**	0.28 (0.22)	-0.11 (0.19)	-0.24 (0.26)	0.39 (0.38)			9	5071.0	5089.1	5.71	0.01
**31**	Φ (HL_{Ind},Tsm,Twt,SR,HL_{Ind}*Twt,HL_{Ind}*SR)	-0.08 (0.19)	**0.72 (0.21)**		-0.13 (0.19)	-0.26 (0.26)		-0.18 (0.39)		9	5071.1	5089.1	5.74	0.01
**32**	Φ (HL_{Ind},HL_{Ind}^2,Tsm,Twt,HL_{Ind}*Twt)	-0.03 (0.20)	**0.62 (0.22)**	0.29 (0.22)	-0.18 (0.20)			-0.05 (0.43)	-0.57 (0.43)	8	5073.4	5089.4	6.04	0.01
**33**	Φ (HL_{Ind},Tsm,Twt,SR,HL_{Ind}*Tsm,HL_{Ind}*Twt)	-0.04 (0.20)	**0.62 (0.22)**	0.28 (0.22)	-0.14 (0.19)	-0.23 (0.26)	0.32 (0.38)		-0.50 (0.40)	9	5071.5	5089.6	6.18	0.01
**34**	Φ (HL_{Ind},HL_{Ind}^2,Tsm,Twt,SR,HL_{Ind}*Tsm,HL_{Ind}*SR)	-0.05 (0.20)	**0.63 (0.22)**	0.27 (0.22)	-0.17 (0.20)		0.45 (0.39)	-0.24 (0.40)		10	5069.5	5089.6	6.21	0.01
**35**	Φ (SR)			**0.46 (0.21)**						4	5081.7	5089.7	6.36	0.01
**36**	Φ (HL_{Ind},HL_{Ind}^2,Tsm,Twt,SR,HL_{Ind}*Twt)	-0.03 (0.20)	**0.63 (0.22)**	0.28 (0.22)	-0.13 (0.19)	-0.26 (0.26)		-0.17 (0.38)		9	5071.8	5089.9	6.52	0.01
**37**	Φ (HL_{Ind},Tsm,Twt,SR, HL_{Ind}*Tsm,HL_{Ind}*Twt,HL_{Ind}*SR)	-0.05 (0.20)	**0.62 (0.22)**	0.28 (0.22)	-0.18 (0.20)		0.37 (0.39)	-0.08 (0.43)	-0.51 (0.43)	10	5070.2	5090.3	6.88	0.00
**38**	Φ (HL_{Ind},HL_{Ind}^2,Tsm,Twt,SR,HL_{Ind}*Twt,HL_{Ind}*SR)	-0.02 (0.20)	**0.63 (0.22)**	0.29 (0.22)	-0.14 (0.20)	-0.25 (0.26)		-0.02 (0.41)	-0.53 (0.41)	10	5070.2	5090.3	6.89	0.00
**39**	Φ (HL_{Ind},HL_{Ind}^2,Tsm,Twt,HL_{Ind}*Tsm,HL_{Ind}*Twt)	-0.10 (0.19)	**0.72 (0.21)**		-0.14 (0.19)	-0.22 (0.26)	0.40 (0.37)	-0.21 (0.39)		9	5072.3	5090.3	6.96	0.00
**HL_{Mat} Models**														
**1**	Φ (Twt,SR)		**0.52 (0.26)**	0.49 (0.25)						5	4234.7	4244.8	0.00	0.11
**2**	Φ (HL_{Mat},HL_{Mat}^2,Twt,SR, HL_{Mat} *SR)		**0.53 (0.26)**	0.50 (0.26)	-0.24 (0.27)	0.63 (0.41)			-0.81 (0.54)	8	4229.9	4245.9	1.16	0.06
**3**	Φ (HL_{Mat},HL_{Mat}^2,Twt,SR)		**0.54 (0.26)**	**0.50 (0.25)**	-0.12 (0.25)	0.56 (0.39)				7	4232.2	4246.2	1.46	0.05
**4**	Φ (Tsm,Twt,SR)	-0.15 (0.23)	**0.53 (0.26)**	0.45 (0.26)						6	4234.3	4246.3	1.56	0.05
**5**	Φ (Twt)		**0.67 (0.25)**							4	4238.6	4246.6	1.82	0.04

**6**	Φ (HL_{Mat},Twt,SR)		**0.52 (0.25)**	0.49 (0.25)	-0.07 (0.22)				6	4234.6	4246.7	1.90	0.04
**7**	Φ (HL_{Mat},Twt,SR, HL_{Mat} *SR)		**0.51 (0.25)**	0.48 (0.26)	-0.17 (0.23)			-0.66 (0.48)	7	4232.7	4246.7	1.97	0.04
**8**	Φ (SR)			**0.65 (0.25)**					4	4238.9	4247.0	2.21	0.04
**9**	Φ (HL_{Mat},HL_{Mat}^2,Tsm,Twt,SR, HL_{Mat} *SR)	-0.18 (0.23)	**0.54 (0.26)**	0.46 (0.26)	-0.24 (0.27)	0.62 (0.41)		-0.85 (0.55)	9	4229.2	4247.3	2.56	0.03
**10**	Φ (Tsm,Twt)	-0.23 (0.22)	**0.68 (0.25)**						5	4237.4	4247.4	2.66	0.03
**11**	Φ (HL_{Mat},HL_{Mat}^2,Twt,SR, HL_{Mat} *Twt)		**0.56 (0.26)**	0.49 (0.26)	-0.21 (0.28)	0.59 (0.40)	-0.47 (0.56)		8	4231.5	4247.5	2.76	0.03
**12**	Φ (HL_{Mat},HL_{Mat}^2,Twt,SR, HL_{Mat} *Twt, HL_{Mat} *SR)		**0.54 (0.26)**	0.49 (0.26)	-0.28 (0.28)	0.65 (0.42)	-0.27 (0.58)	-0.75 (0.56)	9	4229.6	4247.7	2.95	0.03
**13**	Φ (HL_{Mat},HL_{Mat}^2,Tsm,Twt,SR)	-0.14 (0.23)	**0.55 (0.26)**	0.47 (0.26)	-0.11 (0.25)	0.55 (0.39)			8	4231.8	4247.9	3.11	0.02
**14**	Φ (HL_{Mat},Tsm,Twt,SR, HL_{Mat} *SR)	-0.18 (0.23)	**0.53 (0.26)**	0.43 (0.26)	-0.16 (0.23)			-0.7 (0.48)	8	4232.0	4248.1	3.34	0.02
**15**	Φ (HL_{Mat},Twt,SR, HL_{Mat} *Twt)		**0.54 (0.26)**	0.48 (0.25)	-0.14 (0.24)		-0.36 (0.48)		7	4234.1	4248.1	3.35	0.02
**16**	Φ (HL_{Mat},HL_{Mat}^2,Twt)		**0.69 (0.25)**		-0.12 (0.25)	0.54 (0.39)			6	4236.2	4248.2	3.47	0.02
**17**	Φ (HL_{Mat},Tsm,Twt,SR)	-0.15 (0.23)	**0.53 (0.26)**		-0.07 (0.22)				7	4234.2	4248.2	3.48	0.02
**18**	Φ (HL_{Mat},HL_{Mat}^2,SR, HL_{Mat} *SR)			**0.65 (0.25)**	-0.22 (0.27)			-0.87 (0.56)	7	4234.2	4248.3	3.51	0.02
**19**	Φ (HL_{Mat},Twt)		**0.67 (0.25)**		-0.07 (0.22)				5	4238.4	4248.5	3.71	0.02
**20**	Φ (HL_{Mat},Twt,SR, HL_{Mat} *Twt, HL_{Mat} *SR)		**0.52 (0.26)**	0.48 (0.26)	-0.19 (0.24)		-0.19 (0.49)	-0.61 (0.49)	8	4232.5	4248.6	3.83	0.02
**21**	Φ (Tsm,SR)	-0.11 (0.23)		**0.63 (0.25)**					5	4238.7	4248.7	3.98	0.02
**22**	Φ (HL_{Mat},HL_{Mat}^2,SR)			**0.67 (0.25)**	-0.09 (0.25)	0.52 (0.38)			6	4236.7	4248.8	4.02	0.01
**23**	Φ (HL_{Mat},SR, HL_{Mat} *SR)			**0.64 (0.25)**	-0.15 (0.24)			-0.69 (0.49)	6	4236.9	4248.9	4.14	0.01
**24**	Φ (HL_{Mat},SR)			**0.65 (0.25)**	-0.06 (0.22)				5	4238.9	4248.9	4.15	0.01

**25**	Φ (HL$_{Mat}$,HL$_{Mat}^2$,Tsm,Twt,SR, HL$_{Mat}$ *Tsm)	-0.13 (0.23)	**0.56 (0.26)**	0.49 (0.26)	-0.08 (0.25)	0.53 (0.39)	0.46 (0.48)			9	4230.9	4248.9	4.19	0.01
**26**	Φ (HL$_{Mat}$,Tsm,Twt,SR, HL$_{Mat}$ *Tsm)	-0.14 (0.23)	**0.54 (0.26)**	0.48 (0.26)	-0.04 (0.23)		0.47 (0.44)			8	4233.0	4249.1	4.31	0.01
**27**	Φ (HL$_{Mat}$,HL$_{Mat}^2$,Tsm,Twt,SR, HL$_{Mat}$ *Twt)	-0.16 (0.23)	**0.58 (0.26)**	0.45 (0.26)	-0.21 (0.27)	0.59 (0.40)		-0.49 (0.56)		9	4231.0	4249.1	4.32	0.01
**28**	Φ (HL$_{Mat}$,HL$_{Mat}^2$,Tsm,Twt,SR, HL$_{Mat}$ *Twt, HL$_{Mat}$ *SR)	-0.18 (0.23)	**0.56 (0.26)**	0.45 (0.27)	-0.28 (0.28)	0.64 (0.42)		-0.28 (0.58)	-0.79 (0.57)	10	4229.0	4249.1	4.34	0.01
**29**	Φ (HL$_{Mat}$,HL$_{Mat}^2$,Tsm,Twt,SR, HL$_{Mat}$ *Tsm, HL$_{Mat}$ *SR)	-0.18 (0.23)	**0.55 (0.26)**	0.47 (0.27)	-0.21 (0.27)	0.60 (0.41)	0.23 (0.51)		-0.77 (0.58)	10	4229.0	4249.1	4.38	0.01
**30**	Φ (HL$_{Mat}$,HL$_{Mat}^2$,Tsm,Twt)	-0.22 (0.22)	**0.71 (0.25)**		-0.11 (0.25)	0.53 (0.38)				7	4235.1	4249.2	4.44	0.01
**31**	Φ (HL$_{Mat}$,HL$_{Mat}^2$,Twt, HL$_{Mat}$ *Twt)		**0.72 (0.25)**		-0.22 (0.28)	0.59 (0.41)		-0.56 (0.58)		7	4235.2	4249.2	4.49	0.01
**32**	Φ (HL$_{Mat}$,Tsm,Twt)	-0.23 (0.22)	**0.68 (0.25)**		-0.07 (0.22)					6	4237.3	4249.3	4.58	0.01
**33**	Φ (HL$_{Mat}$,Tsm,Twt,SR, HL$_{Mat}$ *Twt)	-0.16 (0.23)	**0.55 (0.26)**	0.44 (0.26)	-0.14 (0.24)			-0.38 (0.48)		8	4233.5	4249.6	4.85	0.01
**34**	Φ (HL$_{Mat}$,Tsm,Twt,SR, HL$_{Mat}$ *Tsm, HL$_{Mat}$ *SR)	-0.18 (0.23)	**0.53 (0.26)**	0.45 (0.26)	-0.13 (0.24)		0.29 (0.46)		-0.60 (0.51)	9	4231.6	4249.7	4.94	0.01
**35**	Φ (HL$_{Mat}$ Twt, HL$_{Mat}$ *Twt)		**0.69 (0.25)**		-0.15 (0.24)			-0.42 (0.49)		6	4237.7	4249.7	4.99	0.01
**36**	Φ (HL$_{Mat}$,HL$_{Mat}^2$,Tsm,SR, HL$_{Mat}$ *SR)	-0.14 (0.23)		**0.63 (0.26)**	-0.22 (0.27)	0.61 (0.41)			-0.90 (0.57)	8	4233.9	4249.9	5.16	0.01
**37**	Φ (HL$_{Mat}$,Tsm,Twt,SR, HL$_{Mat}$ *Twt, HL$_{Mat}$ *SR)	-0.19 (0.23)	**0.54 (0.26)**	0.43 (0.26)	-0.19 (0.24)			-0.21 (0.49)	-0.65 (0.50)	9	4231.9	4249.9	5.18	0.01
**38**	Φ (HL$_{Mat}$,HL$_{Mat}^2$,Tsm,Twt, HL$_{Mat}$ *Twt)	-0.24 (0.22)	**0.73 (0.25)**		-0.22 (0.28)	0.58 (0.40)		-0.59 (0.57)		8	4234.0	4250.1	5.35	0.01
**39**	Φ (HL$_{Mat}$,HL$_{Mat}^2$,Tsm,Twt,SR, HL$_{Mat}$ *Tsm, HL$_{Mat}$ *Twt)	-0.15 (0.23)	**0.58 (0.26)**	0.47 (0.26)	-0.17 (0.28)	0.56 (0.40)	0.45 (0.48)	-0.47 (0.55)		10	4230.1	4250.2	5.46	0.01
**40**	Φ (HL$_{Mat}$,Tsm,Twt,SR, HL$_{Mat}$ *Tsm, HL$_{Mat}$ *Twt)	-0.16 (0.23)	**0.56 (0.26)**	0.46 (0.26)	-0.11 (0.24)		0.47 (0.44)	-0.38 (0.48)		9	4232.4	4250.5	5.71	0.01
**41**	Φ (HL$_{Mat}$,Tsm,Twt, HL$_{Mat}$ *Tsm)	-0.23 (0.22)	**0.70 (0.25)**		-0.04 (0.22)		0.40 (0.43)			7	4236.4	4250.5	5.72	0.01
**42**	Φ (HL$_{Mat}$,Tsm,Twt, HL$_{Mat}$ *Twt)	-0.25 (0.22)	**0.70 (0.25)**		-0.15 (0.24)			-0.45 (0.49)		7	4236.5	4250.5	5.75	0.01
**43**	Φ (HL$_{Mat}$,Tsm,SR, HL$_{Mat}$ *SR)	-0.14 (0.23)		**0.61 (0.25)**	-0.15 (0.24)				-0.72 (0.49)	7	4236.5	4250.5	5.77	0.01

**44**	Φ (HL_{Mat},HL_{Mat}^2,Tsm,Twt, HL_{Mat} *Tsm)	-0.22 (0.22)	**0.72 (0.25)**		-0.09 (0.25)	0.50 (0.39)	0.37 (0.46)			8	4234.5	4250.6	5.81	0.01
**45**	Φ (HL_{Mat},HL_{Mat}^2,Tsm,SR)	-0.10 (0.23)		**0.65 (0.25)**	-0.09 (0.25)	0.52 (0.38)				7	4236.5	4250.6	5.84	0.01
**46**	Φ (HL_{Mat},Tsm,SR)	-0.11 (0.23)		**0.63 (0.25)**	-0.06 (0.22)					6	4238.6	4250.7	5.93	0.01
**47**	Φ (HL_{Mat},HL_{Mat}^2,Tsm,Twt,SR, HL_{Mat} *Tsm, HL_{Mat} *Twt, HL_{Mat} *SR)	-0.18 (0.23)	**0.56 (0.26)**	0.46 (0.27)	-0.25 (0.29)	0.62 (0.42)	0.25 (0.51)	-0.31 (0.57)	-0.69 (0.60)	11	4228.7	4250.9	6.11	0.01
**48**	Φ (HL_{Mat},Tsm,Twt,SR, HL_{Mat} *Tsm, HL_{Mat} *Twt, HL_{Mat} *SR)	-0.18 (0.23)	**0.55 (0.26)**	0.45 (0.26)	-0.16 (0.25)		0.32 (0.46)	-0.24 (0.50)	-0.53 (0.53)	10	4231.4	4251.5	6.72	0.00
**49**	Φ (HL_{Mat},HL_{Mat}^2,Tsm,Twt, HL_{Mat} *Tsm, HL_{Mat} *Twt)	-0.24 (0.22)	**0.74 (0.25)**		-0.20 (0.28)	0.55 (0.41)	0.37 (0.47)	-0.58 (0.57)		9	4233.4	4251.5	6.73	0.00
**50**	Φ (HL_{Mat},Tsm,Twt, HL_{Mat} *Tsm, HL_{Mat} *Twt)	-0.24 (0.22)	**0.71 (0.25)**		-0.12 (0.24)		0.40 (0.43)	-0.45 (0.49)		8	4235.6	4251.6	6.86	0.00
**51**	Φ (HL_{Mat},Tsm,SR, HL_{Mat} *Tsm)	-0.10 (0.23)		**0.66 (0.25)**	-0.03 (0.23)		0.44 (0.44)			7	4237.7	4251.7	6.95	0.00
**HL_{Pat} Models**														
**1**	Φ (HL_{Pat},Twt,SR, HL_{Pat} *SR)		**0.66 (0.27)**	**0.55 (0.27)**	-0.21 (0.24)				**-0.95 (0.48)**	7	3892.9	3907.0	0.00	0.10
**2**	Φ (Tsm,Twt,SR)	-0.35 (0.24)	**0.70 (0.27)**	0.43 (0.27)						6	3895.3	3907.3	0.31	0.09
**3**	Φ (Twt,SR)		**0.65 (0.27)**	**0.53 (0.26)**						5	3897.4	3907.4	0.41	0.08
**4**	Φ (HL_{Pat},Tsm,Twt,SR, HL_{Pat} *SR)	-0.31 (0.25)	**0.71 (0.28)**	0.45 (0.28)	-0.19 (0.24)				-0.90 (0.48)	8	3891.4	3907.4	0.44	0.08
**5**	Φ (Tsm,Twt)	-0.45 (0.23)	**0.87 (0.26)**							5	3897.8	3907.8	0.86	0.07
**6**	Φ (HL_{Pat},Twt,SR)		**0.66 (0.27)**	**0.54 (0.26)**	-0.18 (0.23)					6	3896.8	3908.9	1.86	0.04
**7**	Φ (HL_{Pat},Tsm,Twt,SR)	-0.34 (0.24)	**0.71 (0.27)**	0.44 (0.27)	-0.15 (0.23)					7	3894.8	3908.9	1.90	0.04
**8**	Φ (HL_{Pat},HL_{Pat}^2,Twt,SR, HL_{Pat} *SR)		**0.66 (0.27)**	**0.55 (0.27)**	-0.24 (0.26)	0.11 (0.37)			-0.99 (0.52)	8	3892.9	3908.9	1.93	0.04
**9**	Φ (HL_{Pat},Twt,SR, HL_{Pat} *Twt, HL_{Pat} *SR)		**0.66 (0.27)**	**0.55 (0.27)**	-0.20 (0.25)			0.06 (0.52)	-0.96 (0.51)	8	3892.9	3909.0	2.00	0.04
**10**	Φ (HL_{Pat},Tsm,Twt,SR, HL_{Pat} *Tsm, HL_{Pat} *SR)	-0.31 (0.25)	**0.72 (0.28)**	0.46 (0.28)	-0.18 (0.24)		-0.18 (0.50)		-0.93 (0.50)	9	3891.2	3909.3	2.33	0.03

**11**	Φ (HL_{Pat},HL_{Pat}^2,Tsm,Twt,SR, HL_{Pat} *SR)	-0.31 (0.25)	**0.71 (0.28)**	0.45 (0.28)	-0.21 (0.26)	0.09 (0.37)			-0.94 (0.52)	9	3891.3	3909.4	2.39	0.03
**12**	Φ (HL_{Pat},Tsm,Twt,SR, HL_{Pat} *Twt, HL_{Pat} *SR)	-0.31 (0.25)	**0.71 (0.28)**	0.45 (0.28)	-0.17 (0.25)			0.09 (0.52)	-0.93 (0.51)	9	3891.3	3909.4	2.42	0.03
**13**	Φ (HL_{Pat},Tsm,Twt)	-0.44 (0.23)	**0.88 (0.26)**		-0.13 (0.23)					6	3897.5	3909.6	2.57	0.03
**14**	Φ (Twt)		**0.83 (0.26)**							4	3901.7	3909.7	2.71	0.03
**15**	Φ (HL_{Pat},Twt,SR, HL_{Pat} *Twt)		**0.66 (0.27)**	**0.54 (0.26)**	-0.21 (0.25)			-0.23 (0.50)		7	3896.6	3910.7	3.66	0.02
**16**	Φ (HL_{Pat},Tsm,Twt,SR, HL_{Pat} *Twt)	-0.34 (0.24)	**0.71 (0.27)**	0.44 (0.27)	-0.19 (0.25)			-0.22 (0.50)		8	3894.7	3910.7	3.73	0.02
**17**	Φ (HL_{Pat},HL_{Pat}^2,Twt,SR)		**0.66 (0.27)**	**0.54 (0.26)**	-0.17 (0.24)	-0.02 (0.34)				7	3896.8	3910.9	3.87	0.01
**18**	Φ (HL_{Pat},HL_{Pat}^2,Tsm,Twt,SR)	-0.34 (0.24)	**0.71 (0.27)**	0.44 (0.27)	-0.14 (0.25)	-0.03 (0.34)				8	3894.8	3910.9	3.91	0.01
**19**	Φ (HL_{Pat},Tsm,Twt,SR, HL_{Pat} *Tsm)	-0.34 (0.24)	**0.71 (0.27)**	0.44 (0.27)	-0.15 (0.23)		0.00 (0.50)			8	3894.8	3910.9	3.92	0.01
**20**	Φ (HL_{Pat},HL_{Pat}^2,Twt,SR, HL_{Pat} *Twt, HL_{Pat} *SR)		**0.66 (0.27)**	**0.55 (0.27)**	-0.23 (0.27)	0.10 (0.37)		0.04 (0.54)	-1.00 (0.53)	9	3892.8	3910.9	3.94	0.01
**21**	Φ (HL_{Pat},HL_{Pat}^2,Tsm,Twt,SR, HL_{Pat} *Tsm, HL_{Pat} *SR)	-0.31 (0.25)	**0.71 (0.28)**	0.46 (0.28)	-0.21 (0.26)	0.12 (0.38)	-0.21 (0.52)		-1.00 (0.54)	10	3891.1	3911.2	4.23	0.01
**22**	Φ (HL_{Pat},Tsm,Twt,SR, HL_{Pat} *Tsm, HL_{Pat} *Twt, HL_{Pat} *SR)	-0.34 (0.24)	**0.71 (0.27)**	0.44 (0.27)	-0.16 (0.25)		-0.21 (0.52)	0.15 (0.54)	-0.99 (0.53)	10	3891.2	3911.3	4.27	0.01
**23**	Φ (HL_{Pat},Twt)		**0.84 (0.26)**		-0.15 (0.23)					5	3901.2	3911.3	4.28	0.01
**24**	Φ (HL_{Pat},SR, HL_{Pat} *SR)			**0.75 (0.26)**	-0.18 (0.24)				**-0.98 (0.49)**	6	3899.3	3911.3	4.35	0.01
**25**	Φ (HL_{Pat},HL_{Pat}^2,Tsm,Twt,SR, HL_{Pat} *Twt, HL_{Pat} *SR)	-0.31 (0.25)	**0.71 (0.28)**	0.45 (0.28)	-0.20 (0.27)	0.09 (0.37)		0.07 (0.54)	-0.96 (0.54)	10	3891.3	3911.4	4.39	0.01
**26**	Φ (HL_{Pat},Tsm,Twt, HL_{Pat} *Twt)	-0.44 (0.23)	**0.88 (0.26)**		-0.16 (0.24)			-0.21 (0.50)		7	3897.3	3911.4	4.40	0.01
**27**	Φ (HL_{Pat},Tsm,Twt, HL_{Pat} *Tsm)	-0.44 (0.23)	**0.88 (0.26)**		-0.13 (0.23)		0.04 (0.49)			7	3897.5	3911.6	4.57	0.01
**28**	Φ (HL_{Pat},HL_{Pat}^2,Tsm,Twt)	-0.45 (0.23)	**0.88 (0.26)**		-0.12 (0.24)	-0.03 (0.34)				7	3897.5	3911.6	4.58	0.01
**29**	Φ (SR)			**0.73 (0.25)**						4	3903.7	3911.7	4.69	0.01

**30**	Φ (Tsm,SR)	-0.27 (0.24)		**0.69 (0.25)**						5	3902.4	3912.4	5.46	0.01
**31**	Φ (HL_{Pat},Tsm,SR, HL_{Pat} *SR)	-0.22 (0.25)		**0.71 (0.26)**	-0.16 (0.24)				-0.95 (0.49)	7	3898.5	3912.5	5.53	0.01
**32**	Φ (HL_{Pat},HL_{Pat}^2,Twt,SR, HL_{Pat} *Twt)		**0.66 (0.27)**	**0.54 (0.26)**	-0.22 (0.26)	0.01 (0.34)		-0.23 (0.51)		8	3896.6	3912.7	5.68	0.01
**33**	Φ (HL_{Pat},Tsm,Twt,SR, HL_{Pat} *Tsm, HL_{Pat} *Twt)	-0.34 (0.24)	**0.71 (0.27)**	0.44 (0.27)	-0.19 (0.25)		0.04 (0.51)	-0.22 (0.51)		9	3894.6	3912.7	5.74	0.01
**34**	Φ (HL_{Pat},HL_{Pat}^2,Tsm,Twt,SR, HL_{Pat} *Twt)	-0.34 (0.24)	**0.71 (0.27)**	0.44 (0.27)	-0.18 (0.26)	-0.01 (0.34)		-0.21 (0.51)		9	3894.7	3912.7	5.75	0.01
**35**	Φ (HL_{Pat},HL_{Pat}^2,Tsm,Twt,SR, HL_{Pat} *Tsm)	-0.34 (0.24)	**0.71 (0.27)**	0.44 (0.27)	-0.14 (0.25)	-0.04 (0.34)	0.01 (0.50)			9	3894.8	3912.9	5.92	0.01
**36**	Φ (HL_{Pat},Twt, HL_{Pat} *Twt)		**0.84 (0.26)**		-0.19 (0.24)			-0.23 (0.51)		6	3901.0	3913.1	6.09	0.00
**37**	Φ (HL_{Pat},HL_{Pat}^2,Tsm,Twt,SR, HL_{Pat} *Tsm, HL_{Pat} *Twt, HL_{Pat} *SR)	-0.32 (0.25)	**0.72 (0.28)**	0.46 (0.28)	-0.19 (0.28)	0.11 (0.38)	-0.24 (0.53)	0.13 (0.56)	-1.04 (0.57)	11	3891.1	3913.2	6.20	0.00
**38**	Φ (HL_{Pat},HL_{Pat}^2,SR, HL_{Pat} *SR)			**0.75 (0.26)**	-0.22 (0.26)	0.13 (0.38)			-1.04 (0.53)	7	3899.2	3913.2	6.23	0.00
**39**	Φ (HL_{Pat},HL_{Pat}^2,Twt)		**0.84 (0.26)**		-0.15 (0.24)	0.01 (0.34)				6	3901.2	3913.3	6.29	0.00
**40**	Φ (HL_{Pat},SR)			**0.74 (0.25)**	-0.15 (0.24)					5	3903.3	3913.3	6.31	0.00
**41**	Φ (HL_{Pat},Tsm,Twt, HL_{Pat} *Tsm, HL_{Pat} *Twt)	-0.44 (0.23)	**0.88 (0.26)**		-0.17 (0.25)		0.09 (0.50)	-0.23 (0.52)		8	3897.3	3913.4	6.38	0.00
**42**	Φ (HL_{Pat},HL_{Pat}^2,Tsm,Twt, HL_{Pat} *Twt)	-0.44 (0.23)	**0.88 (0.26)**		-0.16 (0.26)	0.00 (0.34)		-0.21 (0.52)		8	3897.3	3913.4	6.42	0.00
**43**	Φ (HL_{Pat},HL_{Pat}^2,Tsm,Twt, HL_{Pat} *Tsm)	-0.44 (0.23)	**0.88 (0.26)**		-0.12 (0.24)					8	3897.5	3913.6	6.58	0.00

Table S7 Model averaged estimates of an individual's own (IR_{Ind}), maternal (IR_{Mat}) and paternal (IR_{Pat}) internal relatedness effects on their first-year survival probability (Φ) using natural average and zero methods (Burnham and Anderson 2002). Effect sizes where the 95% confidence interval (CI) does not overlap zero are in bold. No. = sequential numbering of each model averaged estimate; Relative importance = sum of Akaike weights of models that contain the effect of interest; SR = total summer rainfall (May–October); Tsm = mean summer temperature (May–October); Twt = mean winter temperature (November–February); IR_{Ind}^2, IR_{Mat}^2 and IR_{Pat}^2 = quadratic effects; * = interaction effect. All predictors were standardised to a mean of 0 and a standard deviation of 2.

No.	Fixed effect	Natural average method		Zero method		
		Estimate	95% CI	Estimate	95% CI	Relative importance
IR_{Ind} models						
1	SR	0.28	-0.15, 0.71	0.15	-0.25, 0.55	0.50
2	Tsm	-0.06	-0.46, 0.33	-0.02	-0.18, 0.14	0.31
3	Twt	**0.66**	**0.23, 1.09**	**0.65**	**0.22, 1.09**	**0.96**
4	IR_{Ind}	-0.10	-0.49, 0.29	-0.06	-0.34, 0.22	0.55
5	IR_{Ind}^2	-0.26	-0.80, 0.28	-0.05	-0.28, 0.17	0.20
6	IR_{Ind}*SR	-0.50	-1.33, 0.32	-0.07	-0.39, 0.25	0.14
7	IR_{Ind}*Tsm	0.32	-0.45, 1.08	0.02	-0.07, 0.11	0.06
8	IR_{Ind}*Twt	-0.21	-1.02, 0.60	-0.03	-0.21, 0.15	0.14
IR_{Mat} models						
9	SR	0.50	-0.02, 1.01	0.40	-0.19, 1.00	0.80
10	Tsm	-0.18	-0.63, 0.27	-0.07	-0.34, 0.21	0.76
11	Twt	**0.57**	**0.05, 1.08**	**0.48**	-0.12, 1.08	0.83
12	IR_{Mat}	-0.10	-0.59, 0.40	-0.07	-0.45, 0.31	0.67
13	IR_{Mat}^2	0.52	-0.25, 1.29	0.18	-0.40, 0.75	0.33
14	IR_{Mat}*SR	-0.81	-1.83, 0.22	-0.26	-1.08, 0.56	0.31
15	IR_{Mat}*Tsm	0.27	-0.66, 1.20	0.02	-0.09, 0.13	0.07
16	IR_{Mat}*Twt	-0.38	-1.46, 0.70	-0.07	-0.42, 0.28	0.19

IR_{Pat} models						
17	SR	0.49	-0.05, 1.03	0.34	-0.21, 0.89	0.68
18	Tsm	-0.38	-2.14, 1.39	-0.26	-0.78, 0.27	0.67
19	Twt	**0.75**	**0.20, 1.30**	**0.72**	**0.10, 1.33**	**0.93**
20	IR_{Pat}	-0.18	-0.66, 0.30	-0.10	-0.48, 0.27	0.57
21	IR_{Pat}^2	-0.21	-0.92, 0.51	-0.03	-0.22, 0.15	0.16
22	IR_{Pat}*SR	-0.61	-1.62, 0.41	-0.08	-0.42, 0.25	0.13
23	IR_{Pat}*Tsm	0.03	-0.99, 1.04	0.003	-0.12, 0.12	0.11
24	IR_{Pat}*Twt	-0.25	-1.27, 0.77	-0.03	-0.24, 0.17	0.13

Table S8 Plausible models, and their model selection statistics, of the effect of an individual's own (IR_{Ind}), maternal (IR_{Mat}) and paternal (IR_{Pat}) internal relatedness on their first-year survival probability. No. = model number; Tsm = mean summer temperature (May–October); Twt = mean winter temperature (November–February); SR = total summer rainfall (May–October); IR_{Ind}^2, IR_{Mat}^2 and IR_{Pat}^2 = quadratic effect; * = interaction; k = number of parameters; QAICc = Akaike's information criterion, corrected for sample size and adjusted through quasi-likelihood; ΔQAICc = difference in QAICc from the top model (i.e., model with lowest QAICc); ω = relative QAICc weight (exp[-0.5 * ΔQAICc], divided by the sum of this quantity for all considered models, whether plausible or not); Φ = first-year survival probability. All plausible models (ΔQAICc < 7) are presented. Effect sizes (standard error) where the 95% confidence interval does not overlap zero are in bold. All predictors were standardised to a mean of 0 and a standard deviation of 2.

**No.**	**Model**	**Tsm (SE)**	**Twt (SE)**	**SR (SE)**	**IR (SE)**	**IR_{Ind}^2 (SE)**	**IR_{Ind}* Tsm (SE)**	**IR_{Ind}* Twt (SE)**	**IR_{Ind}* SR (SE)**	**k**	**Q Deviance**	**QAICc**	**Δ QAICc**	***ω***
**<u>IR_{Ind} Models</u>**														
**1**	Φ (Twt)		**0.69**<br>**(0.21)**							4	5075.4	5083.4	0.00	0.16
**2**	Φ (Twt,SR)		**0.61**<br>**(0.22)**	0.28<br>(0.22)						5	5073.7	5083.7	0.32	0.13
**3**	Φ (IR_{Ind},Twt)		**0.70**<br>**(0.21)**		-0.11<br>(0.20)					5	5075.1	5085.1	1.73	0.07
**4**	Φ (Tsm,Twt)	-0.09<br>(0.20)	**0.70**<br>**(0.21)**							5	5075.2	5085.2	1.80	0.06
**5**	Φ (IR_{Ind},Twt,SR)		**0.61**<br>**(0.22)**	0.28<br>(0.22)	-0.10<br>(0.20)					6	5073.4	5085.4	2.06	0.06
**6**	Φ (Tsm,Twt,SR)	-0.03<br>(0.20)	**0.61**<br>**(0.22)**	0.27<br>(0.22)						6	5073.7	5085.7	2.30	0.05
**7**	Φ (IR_{Ind},Twt,SR, IR_{Ind} *SR)		**0.61**<br>**(0.22)**	0.29<br>(0.22)	-0.14<br>(0.20)				-0.53<br>(0.42)	7	5071.8	5085.9	2.47	0.05
**8**	Φ (IR_{Ind}, IR_{Ind}^2,Twt)		**0.71**<br>**(0.21)**		-0.06<br>(0.20)	-0.27<br>(0.27)				6	5074.1	5086.2	2.79	0.04
**9**	Φ (IR_{Ind}, IR_{Ind}^2,Twt,SR)		**0.63**	0.28	-0.06	-0.27				7	5072.5	5086.5	3.11	0.03

			**(0.22)**	(0.22)	(0.20)	(0.27)								
**10**	Φ (IR_{Ind},Twt, IR_{Ind} *Twt)		**0.70 (0.21)**		-0.13 (0.20)			-0.26 (0.41)		6	5074.7	5086.7	3.33	0.03
**11**	Φ (IR_{Ind},Tsm,Twt)	-0.09 (0.20)	**0.70 (0.21)**		-0.11 (0.20)					6	5074.9	5086.9	3.52	0.03
**12**	Φ (IR_{Ind}, IR_{Ind}^2,Twt,SR, IR_{Ind} *SR)		**0.63 (0.22)**	0.29 (0.22)	-0.09 (0.20)	-0.25 (0.28)			-0.49 (0.41)	8	5071.0	5087.1	3.68	0.03
**13**	Φ (IR_{Ind},Tsm,Twt,SR)	-0.04 (0.20)	**0.61 (0.22)**	0.27 (0.22)	-0.11 (0.20)					7	5073.4	5087.4	4.04	0.02
**14**	Φ (IR_{Ind},Twt,SR, IR_{Ind} *Twt, IR_{Ind} *SR)		**0.61 (0.22)**	0.29 (0.22)	-0.14 (0.20)			-0.11 (0.43)	-0.49 (0.44)	8	5071.7	5087.8	4.41	0.02
**15**	Φ (IR_{Ind},Tsm,Twt,SR, IR_{Ind} *SR)	-0.04 (0.20)	**0.61 (0.22)**	0.28 (0.22)	-0.14 (0.20)				-0.53 (0.42)	8	5071.8	5087.8	4.45	0.02
**16**	Φ (IR_{Ind}, IR_{Ind}^2,Twt, IR_{Ind} *Twt)		**0.72 (0.21)**		-0.08 (0.20)	-0.26 (0.28)		-0.22 (0.39)		7	5073.8	5087.9	4.49	0.02
**17**	Φ (IR_{Ind}, IR_{Ind}^2,Tsm,Twt)	-0.09 (0.20)	**0.71 (0.21)**		-0.06 (0.20)	-0.27 (0.27)				7	5074.0	5088.0	4.61	0.02
**18**	Φ (IR_{Ind},Tsm,Twt, IR_{Ind} *Tsm)	-0.10 (0.20)	**0.71 (0.21)**		-0.10 (0.20)		0.33 (0.39)			7	5074.1	5088.2	4.80	0.01
**19**	Φ (IR_{Ind}, IR_{Ind}^2,Twt,SR, IR_{Ind} *Twt)		**0.63 (0.22)**	0.28 (0.22)	-0.08 (0.20)	-0.26 (0.27)		-0.21 (0.39)		8	5072.2	5088.2	4.83	0.01
**20**	Φ (IR_{Ind}, IR_{Ind}^2,Tsm,Twt,SR)	-0.03 (0.20)	**0.63 (0.22)**	0.27 (0.22)	-0.06 (0.20)	-0.27 (0.27)				8	5072.4	5088.5	5.10	0.01
**21**	Φ (IR_{Ind},Tsm,Twt, IR_{Ind} *Twt)	-0.09 (0.20)	**0.70 (0.21)**		-0.13 (0.20)			-0.27 (0.41)		7	5074.5	5088.5	5.12	0.01
**22**	Φ (IR_{Ind},Tsm,Twt,SR, IR_{Ind} *Tsm)	-0.04 (0.20)	**0.62 (0.22)**	0.27 (0.22)	-0.10 (0.20)		0.34 (0.39)			8	5072.6	5088.7	5.30	0.01
**23**	Φ (IR_{Ind},Tsm,Twt,SR, IR_{Ind} *Twt)	-0.04 (0.20)	**0.62 (0.22)**	0.27 (0.22)	-0.13 (0.20)			-0.26 (0.41)		8	5073.0	5089.0	5.65	0.01
**24**	Φ (IR_{Ind}, IR_{Ind}^2,Twt,SR, IR_{Ind} *Twt, IR_{Ind} *SR)		**0.63 (0.22)**	0.29 (0.22)	-0.10 (0.20)	-0.25 (0.28)		-0.08 (0.41)	-0.46 (0.42)	9	5071.0	5089.0	5.65	0.01
**25**	Φ (IR_{Ind}, IR_{Ind}^2,Tsm,Twt,SR, IR_{Ind} *SR)	-0.03 (0.20)	**0.63 (0.22)**	0.28 (0.22)	-0.09 (0.20)	-0.25 (0.28)			-0.49 (0.41)	9	5071.0	5089.1	5.66	0.01
**26**	Φ (IR_{Ind}, IR_{Ind}^2,Tsm,Twt, IR_{Ind} *Tsm)	-0.09 (0.20)	**0.72 (0.21)**		-0.06 (0.20)	-0.25 (0.28)	0.29 (0.38)			8	5073.4	5089.4	6.02	0.01
**27**	Φ (IR_{Ind},Tsm,Twt,SR, IR_{Ind} *Tsm, IR_{Ind} *SR)	-0.05 (0.20)	**0.62 (0.22)**	0.28 (0.22)	-0.13 (0.20)		0.26 (0.4)		-0.48 (0.43)	9	5071.4	5089.4	6.04	0.01

**28**	Φ (IR_{Ind}, IR_{Ind}^2,Tsm,Twt, IR_{Ind} *Twt)	-0.09<br>(0.20)	**0.72<br>(0.21)**		-0.08<br>(0.20)	-0.26<br>(0.27)		-0.22<br>(0.39)		8	5073.6	5089.7	6.30	0.01
**29**	Φ (SR)			**0.46<br>(0.21)**						4	5081.7	5089.7	6.36	0.01
**30**	Φ (IR_{Ind},Tsm,Twt, IR_{Ind} *Tsm, IR_{Ind} *Twt)	-0.11<br>(0.20)	**0.71<br>(0.21)**		-0.13<br>(0.20)		0.34<br>(0.39)	-0.28<br>(0.41)		8	5073.7	5089.7	6.36	0.01
**31**	Φ (IR_{Ind},Tsm,Twt,SR, IR_{Ind} *Twt, IR_{Ind} *SR)	-0.04<br>(0.20)	**0.62<br>(0.22)**	0.28<br>(0.22)	-0.14<br>(0.20)			-0.11<br>(0.43)	-0.49<br>(0.44)	9	5071.7	5089.8	6.39	0.01
**32**	Φ (IR_{Ind}, IR_{Ind}^2,Tsm,Twt,SR, IR_{Ind} *Tsm)	-0.04<br>(0.20)	**0.63<br>(0.22)**	0.28<br>(0.22)	-0.06<br>(0.20)	-0.25<br>(0.28)	0.30<br>(0.38)			9	5071.8	5089.9	6.51	0.01
**33**	Φ (IR_{Ind}, IR_{Ind}^2,Tsm,Twt,SR, IR_{Ind} *Twt)	-0.03<br>(0.20)	**0.63<br>(0.22)**	0.27<br>(0.22)	-0.08<br>(0.20)	-0.26<br>(0.27)		-0.21<br>(0.39)		9	5072.1	5090.2	6.81	0.01
**IR_{Mat} Models**														
**1**	Φ (Twt,SR)		**0.52<br>(0.26)**	0.49<br>(0.25)						5	4234.7	4244.8	0.00	0.12
**2**	Φ (IR_{Mat}, IR_{Mat}^2,Twt,SR, IR_{Mat} *SR)		**0.53<br>(0.26)**	0.47<br>(0.26)	-0.15<br>(0.26)	0.53<br>(0.39)			-0.85<br>(0.54)	8	4230.2	4246.3	1.51	0.06
**3**	Φ (Tsm,Twt,SR)	-0.15<br>(0.23)	**0.53<br>(0.26)**	0.45<br>(0.26)						6	4234.3	4246.3	1.56	0.05
**4**	Φ (IR_{Mat},Twt,SR, IR_{Mat} *SR)		**0.51<br>(0.25)**	0.47<br>(0.26)	-0.11<br>(0.23)				-0.74<br>(0.48)	7	4232.3	4246.4	1.61	0.05
**5**	Φ (Twt)		**0.67<br>(0.25)**							4	4238.6	4246.6	1.82	0.05
**6**	Φ (IR_{Mat},Twt,SR)		**0.52<br>(0.26)**	0.49<br>(0.25)	-0.03<br>(0.22)					6	4234.7	4246.8	2.00	0.04
**7**	Φ (IR_{Mat}, IR_{Mat}^2,Twt,SR)		**0.54<br>(0.26)**	0.49<br>(0.25)	-0.05<br>(0.25)	0.49<br>(0.38)				7	4232.8	4246.8	2.09	0.04
**8**	Φ (SR)			**0.65<br>(0.25)**						4	4238.9	4247.0	2.21	0.04
**9**	Φ (Tsm,Twt)	-0.23<br>(0.22)	**0.68<br>(0.25)**							5	4237.4	4247.4	2.66	0.03
**10**	Φ (IR_{Mat}, IR_{Mat}^2,Tsm,Twt,SR, IR_{Mat} *SR)	-0.19<br>(0.23)	**0.55<br>(0.26)**	0.42<br>(0.26)	-0.15<br>(0.26)	0.53<br>(0.39)			-0.90<br>(0.54)	9	4229.5	4247.6	2.84	0.03
**11**	Φ (IR_{Mat},Tsm,Twt,SR, IR_{Mat} *SR)	-0.20<br>(0.23)	**0.53<br>(0.26)**	0.42<br>(0.26)	-0.11<br>(0.23)				-0.79<br>(0.49)	8	4231.6	4247.6	2.88	0.03
**12**	Φ (IR_{Mat}, IR_{Mat}^2,Twt,SR, IR_{Mat} *Twt, IR_{Mat} *SR)		**0.55<br>(0.26)**	0.46<br>(0.26)	-0.20<br>(0.27)	0.57<br>(0.41)		-0.31<br>(0.58)	-0.78<br>(0.55)	9	4229.9	4248.0	3.24	0.02

**13**	Φ (IR_{Mat}, IR_{Mat}^2,Twt,SR, IR_{Mat} *Twt)		**0.57 (0.26)**	0.47 (0.26)	-0.15 (0.27)	0.55 (0.40)		-0.50 (0.56)		8	4232.0	4248.0	3.28	0.02
**14**	Φ (IR_{Mat},Twt,SR, IR_{Mat} *Twt)		**0.53 (0.26)**	0.48 (0.25)	-0.09 (0.24)			-0.36 (0.48)		7	4234.2	4248.2	3.46	0.02
**15**	Φ (IR_{Mat},Twt,SR, IR_{Mat} *Twt, IR_{Mat} *SR)		**0.52 (0.26)**	0.47 (0.26)	-0.14 (0.24)			-0.18 (0.50)	-0.70 (0.50)	8	4232.2	4248.3	3.50	0.02
**16**	Φ (IR_{Mat},Tsm,Twt,SR)	-0.15 (0.23)	**0.53 (0.26)**	0.45 (0.26)	-0.02 (0.22)					7	4234.3	4248.3	3.57	0.02
**17**	Φ (IR_{Mat},SR, IR_{Mat} *SR)			**0.63 (0.25)**	-0.09 (0.24)				-0.78 (0.50)	6	4236.4	4248.5	3.71	0.02
**18**	Φ (IR_{Mat}, IR_{Mat}^2,Tsm,Twt,SR)	-0.14 (0.23)	**0.55 (0.26)**	0.45 (0.26)	-0.05 (0.25)	0.48 (0.38)				8	4232.4	4248.5	3.73	0.02
**19**	Φ (IR_{Mat},Twt)		**0.67 (0.25)**		-0.03 (0.22)					5	4238.5	4248.6	3.81	0.02
**20**	Φ (IR_{Mat}, IR_{Mat}^2,Twt)		**0.70 (0.25)**		-0.06 (0.24)	0.50 (0.38)				6	4236.6	4248.6	3.87	0.02
**21**	Φ (IR_{Mat}, IR_{Mat}^2,SR, IR_{Mat} *SR)			**0.63 (0.25)**	-0.13 (0.26)	0.50 (0.4)			-0.91 (0.56)	7	4234.6	4248.6	3.89	0.02
**22**	Φ (Tsm,SR)	-0.11 (0.23)		**0.63 (0.25)**						5	4238.7	4248.7	3.98	0.02
**23**	Φ (IR_{Mat},SR)			**0.65 (0.25)**	0.00 (0.23)					5	4238.9	4249.0	4.22	0.01
**24**	Φ (IR_{Mat}, IR_{Mat}^2,Tsm,Twt,SR, IR_{Mat} *Twt, IR_{Mat} *SR)	-0.20 (0.23)	**0.56 (0.26)**	0.41 (0.26)	-0.20 (0.27)	0.56 (0.41)		-0.32 (0.58)	-0.83 (0.56)	10	4229.2	4249.3	4.54	0.01
**25**	Φ (IR_{Mat},Tsm,Twt)	-0.23 (0.22)	**0.68 (0.25)**		-0.02 (0.22)					6	4237.4	4249.4	4.67	0.01
**26**	Φ (IR_{Mat}, IR_{Mat}^2,SR)			**0.66 (0.25)**	-0.02 (0.25)	0.45 (0.38)				6	4237.4	4249.4	4.68	0.01
**27**	Φ (IR_{Mat},Tsm,Twt,SR, IR_{Mat} *Tsm, IR_{Mat} *SR)	-0.20 (0.23)	**0.53 (0.26)**	0.44 (0.26)	-0.09 (0.24)		0.18 (0.45)		-0.73 (0.51)	9	4231.4	4249.5	4.73	0.01
**28**	Φ (IR_{Mat}, IR_{Mat}^2,Twt, IR_{Mat} *Twt)		**0.72 (0.25)**		-0.18 (0.27)	0.57 (0.41)		-0.61 (0.58)		7	4235.5	4249.5	4.75	0.01
**29**	Φ (IR_{Mat},Tsm,Twt,SR, IR_{Mat} *Twt, IR_{Mat} *SR)	-0.20 (0.23)	**0.54 (0.26)**	0.42 (0.26)	-0.14 (0.24)			-0.19 (0.50)	-0.75 (0.50)	9	4231.4	4249.5	4.75	0.01
**30**	Φ (IR_{Mat},Tsm,Twt,SR, IR_{Mat} *Tsm)	-0.15 (0.23)	**0.54 (0.26)**	0.47 (0.26)	0.01 (0.23)		0.39 (0.44)			8	4233.5	4249.5	4.79	0.01
**31**	Φ (IR_{Mat}, IR_{Mat}^2,Tsm,Twt,SR, IR_{Mat} *Tsm, IR_{Mat} *SR)	-0.19 (0.23)	**0.55 (0.26)**	0.43 (0.27)	-0.14 (0.26)	0.52 (0.39)	0.12 (0.50)		-0.86 (0.57)	10	4229.5	4249.6	4.80	0.01

**32**	Φ (IR_{Mat}, IR_{Mat}^2,Tsm,Twt,SR, IR_{Mat} *Twt)	-0.16 (0.23)	**0.58 (0.26)**	0.43 (0.26)	-0.15 (0.27)	0.54 (0.40)		-0.54 (0.56)		9	4231.5	4249.6	4.80	0.01
**33**	Φ (IR_{Mat}, IR_{Mat}^2,Tsm,Twt)	-0.22 (0.22)	**0.71 (0.25)**		-0.05 (0.24)	0.48 (0.38)				7	4235.6	4249.6	4.86	0.01
**34**	Φ (IR_{Mat},Tsm,Twt,SR, IR_{Mat} *Twt)	-0.17 (0.23)	**0.55 (0.26)**	0.44 (0.26)	-0.09 (0.24)			-0.39 (0.48)		8	4233.6	4249.7	4.94	0.01
**35**	Φ (IR_{Mat},Twt, IR_{Mat} *Twt)		**0.69 (0.25)**		-0.11 (0.24)			-0.42 (0.49)		6	4237.8	4249.8	5.07	0.01
**36**	Φ (IR_{Mat}, IR_{Mat}^2,Tsm,Twt,SR, IR_{Mat} *Tsm)	-0.13 (0.23)	**0.56 (0.26)**	0.47 (0.26)	-0.02 (0.25)	0.46 (0.38)	0.36 (0.47)			9	4231.8	4249.9	5.15	0.01
**37**	Φ (IR_{Mat},Tsm,SR, IR_{Mat} *SR)	-0.16 (0.23)		**0.60 (0.25)**	-0.09 (0.24)				-0.83 (0.50)	7	4236.0	4250.0	5.25	0.01
**38**	Φ (IR_{Mat}, IR_{Mat}^2,Tsm,SR, IR_{Mat} *SR)	-0.15 (0.23)		**0.60 (0.25)**	-0.13 (0.26)	0.50 (0.4)			-0.95 (0.56)	8	4234.2	4250.2	5.48	0.01
**39**	Φ (IR_{Mat}, IR_{Mat}^2,Tsm,Twt, IR_{Mat} *Twt)	-0.24 (0.22)	**0.73 (0.25)**		-0.17 (0.27)	0.56 (0.40)		-0.64 (0.58)		8	4234.3	4250.3	5.59	0.01
**40**	Φ (IR_{Mat},Tsm,Twt, IR_{Mat} *Twt)	-0.25 (0.22)	**0.70 (0.25)**		-0.11 (0.24)			-0.46 (0.49)		7	4236.5	4250.5	5.79	0.01
**41**	Φ (IR_{Mat},Tsm,SR)	-0.11 (0.23)		**0.63 (0.25)**	0.00 (0.23)					6	4238.7	4250.7	5.99	0.01
**42**	Φ (IR_{Mat},Tsm,Twt, IR_{Mat} *Tsm)	-0.23 (0.22)	**0.70 (0.25)**		0.00 (0.22)		0.30 (0.42)			7	4236.9	4250.9	6.16	0.01
**43**	Φ (IR_{Mat},Tsm,Twt,SR, IR_{Mat} *Tsm, IR_{Mat} *Twt)	-0.16 (0.23)	**0.56 (0.26)**	0.46 (0.26)	-0.06 (0.24)		0.38 (0.44)	-0.38 (0.49)		9	4232.8	4250.9	6.17	0.01
**44**	Φ (IR_{Mat}, IR_{Mat}^2,Tsm,Twt,SR, IR_{Mat} *Tsm, IR_{Mat} *Twt)	-0.16 (0.23)	**0.58 (0.26)**	0.45 (0.26)	-0.12 (0.27)	0.52 (0.40)	0.36 (0.48)	-0.52 (0.56)		10	4230.9	4251.0	6.27	0.01
**45**	Φ (IR_{Mat}, IR_{Mat}^2,Tsm,Twt,SR, IR_{Mat}*Tsm, IR_{Mat}*Twt, IR_{Mat}*SR)	-0.19 (0.23)	**0.56 (0.26)**	0.42 (0.27)	-0.19 (0.28)	0.55 (0.41)	0.15 (0.50)	-0.33 (0.58)	-0.78 (0.59)	11	4229.1	4251.2	6.47	0.00
**46**	Φ (IR_{Mat}, IR_{Mat}^2,Tsm,SR)	-0.10 (0.23)		**0.64 (0.25)**	-0.02 (0.25)	0.44 (0.38)				7	4237.2	4251.2	6.49	0.00
**47**	Φ (IR_{Mat}, IR_{Mat}^2,Tsm,Twt, IR_{Mat} *Tsm)	-0.22 (0.22)	**0.72 (0.25)**		-0.03 (0.25)	0.46 (0.38)	0.27 (0.46)			8	4235.2	4251.3	6.52	0.00
**48**	Φ (IR_{Mat},Tsm,Twt,SR, IR_{Mat} *Tsm, IR_{Mat} *Twt, IR_{Mat} *SR)	-0.20 (0.23)	**0.54 (0.26)**	0.43 (0.26)	-0.12 (0.25)		0.20 (0.46)	-0.21 (0.50)	-0.67 (0.53)	10	4231.2	4251.3	6.57	0.00
**IR_{Pat} Models**														
**1**	Φ (Tsm,Twt,SR)	-0.35 (0.24)	**0.70 (0.27)**	0.43 (0.27)						6	3895.3	3907.3	0.00	0.13

**2**	Φ (Twt,SR)		**0.65**<br>**(0.27)**	**0.53**<br>**(0.26)**						5	3897.4	3907.4	0.10	0.12
**3**	Φ (Tsm,Twt)	-0.45<br>(0.23)	**0.87**<br>**(0.26)**							5	3897.8	3907.8	0.55	0.10
**4**	Φ (IR_{Pat},Tsm,Twt,SR)	-0.35<br>(0.24)	**0.71**<br>**(0.27)**	0.44<br>(0.27)	-0.19<br>(0.24)					7	3894.6	3908.7	1.40	0.06
**5**	Φ (IR_{Pat},Twt,SR)		**0.66**<br>**(0.27)**	**0.54**<br>**(0.26)**	-0.19<br>(0.24)					6	3896.7	3908.8	1.46	0.06
**6**	Φ (IR_{Pat},Tsm,Twt,SR, IR_{Pat} *SR)	-0.32<br>(0.25)	**0.71**<br>**(0.27)**	0.46<br>(0.27)	-0.21<br>(0.24)				-0.62<br>(0.51)	8	3893.2	3909.2	1.92	0.05
**7**	Φ (IR_{Pat},Tsm,Twt)	-0.45<br>(0.23)	**0.88**<br>**(0.26)**		-0.16<br>(0.23)					6	3897.3	3909.4	2.07	0.05
**8**	Φ (Twt)		**0.83**<br>**(0.26)**							4	3901.7	3909.7	2.40	0.04
**9**	Φ (IR_{Pat}, IR_{Pat}^2,Tsm,Twt,SR)	-0.36<br>(0.24)	**0.72**<br>**(0.27)**	0.45<br>(0.27)	-0.12<br>(0.25)	-0.23<br>(0.36)				8	3894.2	3910.3	3.00	0.03
**10**	Φ (IR_{Pat},Tsm,Twt,SR, IR_{Pat} *Twt)	-0.35<br>(0.24)	**0.71**<br>**(0.27)**	0.45<br>(0.27)	-0.23<br>(0.25)			-0.31<br>(0.50)		8	3894.3	3910.3	3.03	0.03
**11**	Φ (IR_{Pat}, IR_{Pat}^2,Twt,SR)		**0.66**<br>**(0.27)**	**0.55**<br>**(0.26)**	-0.13<br>(0.25)	-0.22<br>(0.36)				7	3896.4	3910.4	3.12	0.03
**12**	Φ (IR_{Pat},Tsm,Twt,SR, IR_{Pat} *Tsm)	-0.35<br>(0.24)	**0.71**<br>**(0.27)**	0.44<br>(0.27)	-0.19<br>(0.24)		0.02<br>(0.52)			8	3894.6	3910.7	3.41	0.02
**13**	Φ (IR_{Pat}, IR_{Pat}^2,Tsm,Twt,SR, IR_{Pat} *SR)	-0.33<br>(0.25)	**0.72**<br>**(0.27)**	0.46<br>(0.27)	-0.16<br>(0.25)	-0.18<br>(0.37)			-0.58<br>(0.51)	9	3892.9	3911.0	3.70	0.02
**14**	Φ (IR_{Pat}, IR_{Pat}^2,Tsm,Twt)	-0.46<br>(0.23)	**0.89**<br>**(0.26)**		-0.10<br>(0.25)	-0.22<br>(0.36)				7	3897.0	3911.0	3.72	0.02
**15**	Φ (IR_{Pat},Tsm,Twt, IR_{Pat} *Twt)	-0.45<br>(0.23)	**0.88**<br>**(0.26)**		-0.21<br>(0.25)			-0.29<br>(0.51)		7	3897.0	3911.1	3.75	0.02
**16**	Φ (IR_{Pat},Tsm,Twt,SR, IR_{Pat} *Twt, IR_{Pat} *SR)	-0.32<br>(0.25)	**0.72**<br>**(0.27)**	0.46<br>(0.27)	-0.23<br>(0.25)			-0.14<br>(0.53)	-0.58<br>(0.53)	9	3893.1	3911.2	3.86	0.02
**17**	Φ (IR_{Pat},Twt)		**0.84**<br>**(0.26)**		-0.17<br>(0.23)					5	3901.2	3911.2	3.90	0.02
**18**	Φ (IR_{Pat},Tsm,Twt,SR, IR_{Pat} *Tsm, IR_{Pat} *SR)	-0.33<br>(0.25)	**0.72**<br>**(0.27)**	0.46<br>(0.28)	-0.21<br>(0.24)		-0.08<br>(0.52)		-0.64<br>(0.52)	9	3893.1	3911.2	3.91	0.02
**19**	Φ (IR_{Pat},Tsm,Twt, IR_{Pat} *Tsm)	-0.45<br>(0.23)	**0.88**<br>**(0.26)**		-0.16<br>(0.23)		0.07<br>(0.50)			7	3897.3	3911.4	4.06	0.02
**20**	Φ (SR)			**0.73**<br>**(0.25)**						4	3903.7	3911.7	4.38	0.01

**21**	Φ (IR_{Pat}, IR_{Pat}^2,Tsm,Twt,SR, IR_{Pat} *Twt)	-0.35<br>(0.24)	**0.72**<br>**(0.27)**	0.45<br>(0.27)	-0.17<br>(0.27)	-0.19<br>(0.37)		-0.24<br>(0.50)		9	3894.0	3912.1	4.79	0.01
**22**	Φ (IR_{Pat}, IR_{Pat}^2,Tsm,Twt,SR, IR_{Pat} *Tsm)	-0.35<br>(0.25)	**0.72**<br>**(0.27)**	0.44<br>(0.27)	-0.12<br>(0.25)	-0.24<br>(0.36)	0.04<br>(0.51)			9	3894.2	3912.3	5.01	0.01
**23**	Φ (IR_{Pat},Tsm,Twt,SR, IR_{Pat} *Tsm, IR_{Pat} *Twt)	-0.34<br>(0.24)	**0.71**<br>**(0.27)**	0.44<br>(0.27)	-0.24<br>(0.25)		0.09<br>(0.53)	-0.33<br>(0.52)		9	3894.2	3912.3	5.02	0.01
**24**	Φ (Tsm,SR)	-0.27<br>(0.24)		**0.69**<br>**(0.25)**						5	3902.4	3912.4	5.15	0.01
**25**	Φ (IR_{Pat}, IR_{Pat}^2,Tsm,Twt, IR_{Pat} *Twt)	-0.46<br>(0.23)	**0.89**<br>**(0.26)**		-0.15<br>(0.27)	-0.18<br>(0.37)		-0.22<br>(0.51)		8	3896.8	3912.8	5.54	0.01
**26**	Φ (IR_{Pat},Twt, IR_{Pat} *Twt)		**0.84**<br>**(0.26)**		-0.22<br>(0.25)			-0.31<br>(0.52)		6	3900.8	3912.8	5.54	0.01
**27**	Φ (IR_{Pat}, IR_{Pat}^2,Twt)		**0.84**<br>**(0.26)**		-0.12<br>(0.25)	-0.18<br>(0.36)				6	3900.9	3913.0	5.67	0.01
**28**	Φ (IR_{Pat},Tsm,Twt, IR_{Pat} *Tsm, IR_{Pat} *Twt)	-0.44<br>(0.23)	**0.88**<br>**(0.26)**		-0.22<br>(0.25)		0.15<br>(0.51)	-0.33<br>(0.52)		8	3896.9	3913.0	5.69	0.01
**29**	Φ (IR_{Pat}, IR_{Pat}^2,Tsm,Twt,SR, IR_{Pat} *Twt, IR_{Pat} *SR)	-0.33<br>(0.25)	**0.72**<br>**(0.27)**	0.46<br>(0.27)	-0.18<br>(0.27)	-0.16<br>(0.38)		-0.09<br>(0.52)	-0.55<br>(0.53)	10	3892.9	3913.0	5.69	0.01
**30**	Φ (IR_{Pat}, IR_{Pat}^2,Tsm,Twt, IR_{Pat} *Tsm)	-0.45<br>(0.23)	**0.89**<br>**(0.26)**		-0.10<br>(0.25)	-0.22<br>(0.36)	0.09<br>(0.49)			8	3896.9	3913.0	5.70	0.01
**31**	Φ (IR_{Pat}, IR_{Pat}^2,Tsm,Twt,SR, IR_{Pat} *Tsm, IR_{Pat} *SR)	-0.33<br>(0.25)	**0.72**<br>**(0.27)**	0.46<br>(0.28)	-0.16<br>(0.26)	-0.17<br>(0.37)	-0.06<br>(0.51)		-0.59<br>(0.52)	10	3892.9	3913.0	5.71	0.01
**32**	Φ (IR_{Pat},SR)			**0.74**<br>**(0.25)**	-0.18<br>(0.24)					5	3903.1	3913.2	5.85	0.01
**33**	Φ (IR_{Pat},Tsm,Twt,SR, IR_{Pat} *Tsm, IR_{Pat} *Twt, IR_{Pat} *SR)	-0.32<br>(0.25)	**0.72**<br>**(0.27)**	0.46<br>(0.28)	-0.23<br>(0.25)		-0.05<br>(0.54)	-0.13<br>(0.55)	-0.59<br>(0.55)	10	3893.1	3913.2	5.87	0.01
**34**	Φ (IR_{Pat},SR, IR_{Pat} *SR)			**0.76**<br>**(0.26)**	-0.20<br>(0.24)				-0.68<br>(0.52)	6	3901.4	3913.4	6.14	0.01
**35**	Φ (IR_{Pat},Tsm,SR)	-0.26<br>(0.24)		**0.7**<br>**(0.25)**	-0.17<br>(0.24)					6	3901.9	3914.0	6.67	0.00
**36**	Φ (IR_{Pat}, IR_{Pat}^2,Tsm,Twt,SR, IR_{Pat} *Tsm, IR_{Pat} *Twt)	-0.35<br>(0.25)	**0.72**<br>**(0.27)**	0.45<br>(0.27)	-0.17<br>(0.27)	-0.20<br>(0.37)	0.09<br>(0.52)	-0.26<br>(0.51)		10	3894.0	3914.1	6.77	0.00

Table S9 Number of offspring in the datasets depending on number of known parents and grandparents, grouped by their coefficient of inbreeding (*f*)

	Both parents known	At least one grandparent known	All four grandparents known
***f* = 0.0**	536	395	85
***f* = 0.125**	8	8	2
***f* = 0.25**	17	17	1
**Total**	561	420	88

Table S10 Plausible models, and their model selection statistics, of the effect of an individual's own (SH_{Ind}), maternal (SH_{Mat}) and paternal (SH_{Pat}) standardised heterozygosity on their first-year survival probability. No. = model number; Tsm = mean summer temperature (May–October); Twt = mean winter temperature (November–February); SR = total summer rainfall (May–October); SH_{Ind}^2, SH_{Mat}^2 and SH_{Pat}^2 = quadratic effect; * = interaction; k = number of parameters; QAICc = Akaike's information criterion, corrected for sample size and adjusted through quasi-likelihood; ΔQAICc = difference in QAICc from the top model (i.e., model with lowest QAICc); ω = relative QAICc weight (exp[-0.5 * ΔQAICc], divided by the sum of this quantity for all considered models, whether plausible or not); Φ = first-year survival probability. All plausible models (ΔQAICc < 7) are presented. Effect sizes (standard error) where the 95% confidence interval does not overlap zero are in bold. All predictors were standardised to a mean of 0 and a standard deviation of 2.

**No.**	**Model**	**Tsm (SE)**	**Twt (SE)**	**SR (SE)**	**SH (SE)**	**SH_{Ind}^2 (SE)**	**SH_{Ind}* Tsm (SE)**	**SH_{Ind}* Twt (SE)**	**SH_{Ind}* SR (SE)**	**k**	**Q Deviance**	**QAICc**	**Δ QAICc**	***ω***
**<u>SH_{Ind} Models</u>**														
**1**	Φ (Twt)		**0.69 (0.21)**							4	5075.4	5083.4	0.00	0.11
**2**	Φ (Twt,SR)		**0.61 (0.22)**	0.28 (0.22)						5	5073.7	5083.7	0.32	0.09
**3**	Φ (SH_{Ind},Twt)		**0.70 (0.21)**		0.23 (0.20)					5	5074.1	5084.1	0.71	0.08
**4**	Φ (SH_{Ind},Twt,SR)		**0.61 (0.22)**	0.28 (0.22)	0.23 (0.20)					6	5072.4	5084.4	1.02	0.06
**5**	Φ (SH_{Ind},Twt,SR,SH_{Ind}*SR)		**0.62 (0.22)**	0.30 (0.22)	0.26 (0.20)				0.55 (0.42)	7	5070.7	5084.7	1.33	0.06
**6**	Φ (SH_{Ind},SH_{Ind}^2,Twt)		**0.72 (0.21)**		0.20 (0.19)	-0.31 (0.27)				6	5072.9	5084.9	1.53	0.05
**7**	Φ (SH_{Ind},SH_{Ind}^2,Twt,SR)		**0.63 (0.22)**	0.29 (0.22)	0.20 (0.19)	-0.31 (0.27)				7	5071.1	5085.2	1.80	0.04
**8**	Φ (Tsm,Twt)	-0.09 (0.20)	**0.70 (0.21)**							5	5075.2	5085.2	1.80	0.04
**9**	Φ (Tsm,Twt,SR)	-0.03	**0.61**	0.27						6	5073.7	5085.7	2.30	0.03

		(0.20)	**(0.22)**	(0.22)										
**10**	$\Phi\ (SH_{Ind},SH_{Ind}^2,Twt,SR,SH_{Ind}*SR)$		**0.63 (0.22)**	0.30 (0.22)	0.23 (0.20)	-0.29 (0.28)			0.49 (0.41)	8	5069.7	5085.7	2.35	0.03
**11**	$\Phi\ (SH_{Ind},Twt,SH_{Ind}*Twt)$		**0.71 (0.21)**		0.25 (0.21)			0.23 (0.42)		6	5073.8	5085.8	2.40	0.03
**12**	$\Phi\ (SH_{Ind},Tsm,Twt)$	-0.09 (0.20)	**0.70 (0.21)**		0.23 (0.20)					6	5073.9	5085.9	2.49	0.03
**13**	$\Phi\ (SH_{Ind},Twt,SR,SH_{Ind}*Twt)$		**0.62 (0.22)**	0.28 (0.22)	0.25 (0.21)			0.22 (0.41)		7	5072.1	5086.1	2.74	0.03
**14**	$\Phi\ (SH_{Ind},Tsm,Twt,SR)$	-0.04 (0.20)	**0.62 (0.22)**	0.27 (0.22)	0.23 (0.20)					7	5072.3	5086.4	3.00	0.02
**15**	$\Phi\ (SH_{Ind},Tsm,Twt,SR,SH_{Ind}*SR)$	-0.04 (0.20)	**0.62 (0.22)**	0.29 (0.23)	0.26 (0.20)				0.55 (0.42)	8	5070.7	5086.7	3.32	0.02
**16**	$\Phi\ (SH_{Ind},Twt,SR,SH_{Ind}*Twt,SH_{Ind}*SR)$		**0.62 (0.22)**	0.30 (0.22)	0.27 (0.21)			0.07 (0.44)	0.53 (0.44)	8	5070.7	5086.7	3.32	0.02
**17**	$\Phi\ (SH_{Ind},SH_{Ind}^2,Twt,SH_{Ind}*Twt)$		**0.72 (0.21)**		0.22 (0.20)	-0.29 (0.28)		0.17 (0.39)		7	5072.7	5086.7	3.34	0.02
**18**	$\Phi\ (SH_{Ind},SH_{Ind}^2,Tsm,Twt)$	-0.08 (0.20)	**0.72 (0.21)**		0.20 (0.19)	-0.30 (0.27)				7	5072.7	5086.7	3.36	0.02
**19**	$\Phi\ (SH_{Ind},SH_{Ind}^2,Twt,SR,SH_{Ind}*Twt)$		**0.64 (0.22)**	0.28 (0.22)	0.22 (0.20)	-0.30 (0.28)		0.16 (0.39)		8	5071.0	5087.0	3.64	0.02
**20**	$\Phi\ (SH_{Ind},SH_{Ind}^2,Tsm,Twt,SR)$	-0.03 (0.20)	**0.63 (0.22)**	0.28 (0.22)	0.20 (0.19)	-0.31 (0.28)				8	5071.1	5087.2	3.79	0.02
**21**	$\Phi\ (SH_{Ind},Tsm,Twt,SH_{Ind}*Tsm)$	-0.10 (0.20)	**0.71 (0.21)**		0.23 (0.20)		-0.22 (0.39)			7	5073.5	5087.6	4.17	0.01
**22**	$\Phi\ (SH_{Ind},Tsm,Twt,SH_{Ind}*Twt)$	-0.09 (0.20)	**0.71 (0.21)**		0.25 (0.21)		0.23 (0.41)			7	5073.5	5087.6	4.18	0.01
**23**	$\Phi\ (SH_{Ind},SH_{Ind}^2,Tsm,Twt,SR,SH_{Ind}*SR)$	-0.03 (0.20)	**0.63 (0.22)**	0.29 (0.23)	0.23 (0.20)	-0.28 (0.28)			0.49 (0.41)	9	5069.7	5087.7	4.35	0.01
**24**	$\Phi\ (SH_{Ind},SH_{Ind}^2,Twt,SR,SH_{Ind}*Twt,SH_{Ind}*SR)$		**0.63 (0.22)**	0.30 (0.22)	0.23 (0.20)	-0.28 (0.28)		0.02 (0.41)	0.48 (0.43)	9	5069.7	5087.7	4.36	0.01
**25**	$\Phi\ (SH_{Ind},Tsm,Twt,SR,SH_{Ind}*Tsm)$	-0.05 (0.20)	**0.62 (0.22)**	0.27 (0.22)	0.23 (0.20)		-0.22 (0.40)			8	5072.0	5088.1	4.69	0.01
**26**	$\Phi\ (SH_{Ind},Tsm,Twt,SR,SH_{Ind}*Twt)$	-0.04 (0.20)	**0.63 (0.22)**	0.27 (0.22)	0.25 (0.21)			0.23 (0.41)		8	5072.0	5088.1	4.71	0.01
**27**	$\Phi\ (SH_{Ind},SH_{Ind}^2,Tsm,Twt,SH_{Ind}*Tsm)$	-0.09 (0.20)	**0.72 (0.21)**		0.21 (0.19)	-0.29 (0.28)	-0.18 (0.38)			8	5072.5	5088.5	5.13	0.01

**28**	Φ (SH$_{Ind}$,SH$_{Ind}^2$,Tsm,Twt,SH$_{Ind}$*Twt)	-0.09 (0.20)	**0.73 (0.21)**		0.22 (0.20)	-0.29 (0.28)		0.18 (0.39)		8	5072.5	5088.5	5.16	0.01
**29**	Φ (SH$_{Ind}$,Tsm,Twt,SR,SH$_{Ind}$*Tsm,SH$_{Ind}$*SR)	-0.04 (0.20)	**0.62 (0.22)**	0.29 (0.23)	0.26 (0.20)		-0.14 (0.40)		0.52 (0.43)	9	5070.5	5088.6	5.20	0.01
**30**	Φ (SH$_{Ind}$,Tsm,Twt,SR,SH$_{Ind}$*Twt,SH$_{Ind}$*SR)	-0.04 (0.20)	**0.62 (0.22)**	0.29 (0.23)	0.27 (0.21)			0.07 (0.44)	0.52 (0.44)	9	5070.6	5088.7	5.31	0.01
**31**	Φ (SH$_{Ind}$,SH$_{Ind}^2$,T$_s$m,Twt,SR,SH$_{Ind}$*Tsm)	-0.03 (0.20)	**0.64 (0.22)**	0.28 (0.22)	0.21 (0.19)	-0.30 (0.28)	-0.18 (0.38)			9	5070.9	5089.0	5.58	0.01
**32**	Φ (SH$_{Ind}$,SH$_{Ind}^2$,Tsm,Twt,SR,SH$_{Ind}$*Twt)	-0.03 (0.20)	**0.64 (0.22)**	0.28 (0.22)	0.22 (0.20)	-0.30 (0.28)		0.17 (0.39)		9	5070.9	5089.0	5.63	0.01
**33**	Φ (SH$_{Ind}$,Tsm,Twt,SH$_{Ind}$*Tsm,SH$_{Ind}$*T$_w$t)	-0.10 (0.20)	**0.72 (0.21)**		0.26 (0.21)		-0.24 (0.39)	0.25 (0.41)		8	5073.2	5089.2	5.82	0.01
**34**	Φ (SH$_{Ind}$,SH$_{Ind}^2$,T$_s$m,Twt,SR,SH$_{Ind}$*Tsm,SH$_{Ind}$*SR)	-0.03 (0.20)	**0.63 (0.22)**	0.29 (0.23)	0.23 (0.20)	-0.28 (0.28)	-0.12 (0.39)		0.47 (0.41)	10	5069.6	5089.7	6.26	0.00
**35**	Φ (SH$_{Ind}$,SH$_{Ind}^2$,Tsm,T$_w$t,SR,SH$_{Ind}$*Twt,SH$_{Ind}$*R)	-0.03 (0.20)	**0.63 (0.22)**	0.29 (0.23)	0.23 (0.20)	-0.28 (0.28)		0.02 (0.41)	0.48 (0.43)	10	5069.7	5089.7	6.36	0.00
**36**	Φ (SR)			**0.46 (0.21)**						4	5081.7	5089.7	6.36	0.00
**37**	Φ (SH$_{Ind}$,Tsm,Twt,SR,SH$_{Ind}$*Tsm,SH$_{Ind}$*Twt)	-0.05 (0.20)	**0.63 (0.22)**	0.27 (0.22)	0.25 (0.21)		-0.23 (0.40)	0.23 (0.41)		9	5071.7	5089.8	6.38	0.00
**38**	Φ (SH$_{Ind}$,SH$_{Ind}^2$,Tsm,Twt,SH$_{Ind}$*Tsm,SH$_{Ind}$*Twt)	-0.09 (0.20)	**0.73 (0.21)**		0.23 (0.20)	-0.28 (0.28)	-0.20 (0.38)	0.19 (0.39)		9	5072.2	5090.3	6.90	0.00
**SH$_{Mat}$ Models**														
**1**	Φ (Twt,SR)		**0.51 (0.25)**	**0.49 (0.25)**						5	4361.8	4371.8	0.00	0.13
**2**	Φ (SH$_{Mat}$,Twt,SR,SH$_{Mat}$*SR)		**0.52 (0.25)**	0.49 (0.25)	0.21 (0.24)				0.81 (0.48)	7	4358.7	4372.7	0.91	0.08
**3**	Φ (Tsm,Twt,SR)	-0.15 (0.22)	**0.53 (0.25)**	0.45 (0.25)						6	4361.3	4373.3	1.55	0.06
**4**	Φ (SH$_{Mat}$,SH$_{Mat}^2$,Twt,SR,SH$_{Mat}$*SR)		**0.53 (0.25)**	**0.50 (0.26)**	0.23 (0.26)	0.42 (0.40)			0.95 (0.53)	8	4357.5	4373.5	1.73	0.05
**5**	Φ (SH$_{Mat}$,Twt,SR)		**0.52 (0.25)**	0.49 (0.25)	0.09 (0.22)					6	4361.6	4373.7	1.86	0.05
**6**	Φ (Twt)		**0.67 (0.24)**							4	4365.7	4373.7	1.94	0.05
**7**	Φ (SH$_{Mat}$, Tsm,Twt,SR,SH$_{Mat}$*SR)	-0.18	**0.53**	0.44	0.21				0.84	8	4358.0	4374.1	2.29	0.04

		(0.23)	**(0.25)**	(0.26)	(0.24)				(0.48)					
**8**	Φ (R)			**0.65 (0.24)**						4	4366.1	4374.1	2.34	0.04
**9**	Φ (Tsm,Twt)	-0.23 (0.21)	**0.68 (0.24)**							5	4364.5	4374.5	2.74	0.03
**10**	Φ (SH_{Mat},Twt,SR,H*Twt,SH_{Mat}*SR)		**0.52 (0.25)**	0.48 (0.25)	0.22 (0.25)			0.10 (0.50)	0.79 (0.50)	8	4358.6	4374.7	2.89	0.03
**11**	Φ (SH_{Mat},SH_{Mat}^2,Tsm,Twt,SR,SH_{Mat}*SR)	-0.18 (0.23)	**0.54 (0.25)**	0.46 (0.26)	0.23 (0.26)	0.42 (0.40)			0.99 (0.53)	9	4356.8	4374.9	3.08	0.03
**12**	Φ (SH_{Mat},SH_{Mat}^2,Twt,SR)		**0.53 (0.25)**	**0.50 (0.25)**	0.09 (0.24)	0.30 (0.38)				7	4360.9	4375.0	3.19	0.03
**13**	Φ (SH_{Mat},SR,SH_{Mat}*SR)			**0.64 (0.25)**	0.18 (0.24)				0.84 (0.49)	6	4363.0	4375.0	3.25	0.02
**14**	Φ (SH_{Mat},Tsm,Twt,SR)	-0.15 (0.22)	**0.53 (0.25)**	0.45 (0.26)	0.08 (0.22)					7	4361.2	4375.2	3.43	0.02
**15**	Φ (SH_{Mat},Twt,SR,SH_{Mat}*Twt)		**0.54 (0.25)**	0.48 (0.25)	0.14 (0.24)			0.31 (0.48)		7	4361.2	4375.2	3.44	0.02
**16**	Φ (SH_{Mat},SH_{Mat}^2,Twt,SR,SH_{Mat}*Twt,SH_{Mat}*SR)		**0.54 (0.26)**	0.50 (0.26)	0.26 (0.27)	0.44 (0.41)		0.17 (0.56)	0.92 (0.54)	9	4357.4	4375.4	3.65	0.02
**17**	Φ (SH_{Mat},Twt)		**0.67 (0.24)**		0.09 (0.22)					5	4365.5	4375.6	3.77	0.02
**18**	Φ (Tsm,SR)	-0.11 (0.22)		**0.63 (0.24)**						5	4365.9	4375.9	4.10	0.02
**19**	Φ (SH_{Mat},SH_{Mat}^2,SR,SH_{Mat}*SR)			**0.66 (0.25)**	0.20 (0.26)	0.40 (0.40)			0.99 (0.54)	7	4361.9	4375.9	4.14	0.02
**20**	Φ (SH_{Mat},Tsm,Twt,SR,SH_{Mat}*Twt,SH_{Mat}*SR)	-0.18 (0.23)	**0.54 (0.25)**	0.44 (0.26)	0.22 (0.25)			0.10 (0.49)	0.82 (0.50)	9	4358.0	4376.1	4.27	0.01
**21**	Φ (SH_{Mat},Tsm,Twt,SR,SH_{Mat}*Tsm,SH_{Mat}*SR)	-0.18 (0.23)	**0.53 (0.25)**	0.44 (0.26)	0.20 (0.24)		-0.09 (0.44)		0.82 (0.50)	9	4358.0	4376.1	4.27	0.01
**22**	Φ (SH_{Mat},SR)			**0.65 (0.24)**	0.06 (0.22)					5	4366.0	4376.1	4.27	0.01
**23**	Φ (SH_{Mat},Tsm,Twt)	-0.23 (0.21)	**0.68 (0.24)**		0.08 (0.22)					6	4364.4	4376.4	4.61	0.01
**24**	Φ (SH_{Mat},SH_{Mat}^2,Twt,SR,SH_{Mat}*Twt)		**0.55 (0.26)**	0.49 (0.25)	0.17 (0.26)	0.35 (0.40)		0.41 (0.55)		8	4360.3	4376.4	4.61	0.01
**25**	Φ (SH_{Mat},SH_{Mat}^2, Tsm,Twt,SR)	-0.15 (0.22)	**-0.15 (0.22)**	0.46 (0.26)	0.08 (0.24)	0.30 (0.38)				8	4360.5	4376.6	4.76	0.01

**26**	Φ (SH$_{Mat}$,Tsm,SR,SH$_{Mat}$*SR)	-0.14 (0.23)		**0.62 (0.25)**	0.18 (0.24)				0.86 (0.49)	7	4362.6	4376.7	4.89	0.01
**27**	Φ (SH$_{Mat}$,Tsm,Twt,SR,SH$_{Mat}$*Twt)	-0.16 (0.22)	0.55 (0.25)	**0.44 (0.26)**	0.14 (0.24)			0.33 (0.48)		8	4360.7	4376.8	4.97	0.01
**28**	Φ (SH$_{Mat}$,Tsm,Twt,SR,SH$_{Mat}$* Tsm)	-0.15 (0.22)	0.54 (0.25)	**0.46 (0.26)**	0.07 (0.22)		-0.29 (0.43)			8	4360.7	4376.8	5.00	0.01
**29**	Φ (SH$_{Mat}$,SH$_{Mat}^2$,Tsm,Twt,SR,SH$_{Mat}$*Twt,SH$_{Mat}$*SR)	-0.19 (0.23)	**0.55 (0.26)**	0.45 (0.26)	0.25 (0.27)	0.44 (0.41)		0.17 (0.56)	0.95 (0.55)	10	4356.7	4376.8	5.00	0.01
**30**	Φ (SH$_{Mat}$,SH$_{Mat}^2$,Tsm,Twt,SR,SH$_{Mat}$*Tsm,SH$_{Mat}$*SR)	-0.19 (0.23)	**0.54 (0.25)**	0.46 (0.26)	0.23 (0.26)	0.42 (0.40)	-0.03 (0.47)		0.98 (0.55)	10	4356.8	4376.9	5.09	0.01
**31**	Φ (SH$_{Mat}$,SH$_{Mat}^2$,Twt)		**0.68 (0.25)**		0.09 (0.23)	0.28 (0.38)				6	4365.0	4377.0	5.21	0.01
**32**	Φ (SH$_{Mat}$,Twt,SH$_{Mat}$*Twt)		**0.69 (0.25)**		0.16 (0.24)			0.37 (0.49)		6	4365.0	4377.0	5.21	0.01
**33**	Φ (SH$_{Mat}$,SH$_{Mat}^2$,SR)			**0.66 (0.24)**	0.05 (0.24)	0.27 (0.37)				6	4365.5	4377.5	5.72	0.01
**34**	Φ (SH$_{Mat}$,SH$_{Mat}^2$,Tsm,SR,SH$_{Mat}$*SR)	-0.14 (0.23)		**0.63 (0.25)**	0.20 (0.26)	0.41 (0.40)			1.02 (0.55)	8	4361.5	4377.6	5.76	0.01
**35**	Φ (SH$_{Mat}$,Tsm,Twt,SH$_{Mat}$*Twt)	-0.24 (0.21)	**0.70 (0.24)**		0.16 (0.24)			0.39 (0.49)		7	4363.7	4377.8	5.99	0.01
**36**	Φ (SH$_{Mat}$,Tsm,SR)	-0.11 (0.22)		**0.63 (0.24)**	0.06 (0.22)					6	4365.8	4377.8	6.04	0.01
**37**	Φ (SH$_{Mat}$,SH$_{Mat}^2$,Tsm,Twt)	-0.23 (0.21)	**0.70 (0.24)**		0.09 (0.23)	0.28 (0.38)				7	4363.8	4377.8	6.05	0.01
**38**	Φ (SH$_{Mat}$,SH$_{Mat}^2$,Tsm,Twt,SR,SH$_{Mat}$*Twt)	-0.16 (0.23)	**0.57 (0.26)**	0.45 (0.26)	0.16 (0.26)	0.35 (0.41)		0.43 (0.55)		9	4359.9	4377.9	6.13	0.01
**39**	Φ (SH$_{Mat}$, Tsm,Twt,SR,SH$_{Mat}$*Tsm,SH$_{Mat}$*Twt,SH$_{Mat}$*SR)	-0.18 (0.23)	**0.54 (0.25)**	0.44 (0.26)	0.21 (0.25)		-0.10 (0.45)	0.11 (0.50)	0.78 (0.52)	10	4357.9	4378.0	6.23	0.01
**40**	Φ (SH$_{Mat}$,Tsm,Twt,SH$_{Mat}$*Tsm)	-0.23 (0.21)	**0.69 (0.24)**		0.07 (0.22)		-0.23 (0.42)			7	4364.1	4378.1	6.32	0.01
**41**	Φ (SH$_{Mat}$,SH$_{Mat}^2$,Tsm,Twt,SR,SH$_{Mat}$*Tsm)	-0.15 (0.22)	**0.55 (0.25)**	0.47 (0.26)	0.07 (0.24)	0.28 (0.38)	-0.27 (0.45)			9	4360.1	4378.2	6.42	0.01
**42**	Φ (SH$_{Mat}$,SH$_{Mat}^2$,Twt,SH$_{Mat}$*Twt)		**0.71 (0.25)**		0.19 (0.27)	0.35 (0.42)		0.48 (0.57)		7	4364.2	4378.2	6.45	0.01
**43**	Φ (SH$_{Mat}$,Tsm,Twt,SR,SH$_{Mat}$*Tsm,SH$_{Mat}$*Twt)	-0.16 (0.23)	**0.55 (0.25)**	0.45 (0.26)	0.13 (0.24)		-0.29 (0.43)	0.33 (0.48)		9	4360.2	4378.3	6.53	0.00

**44**	Φ (SH_{Mat},Tsm,SR,SH_{Mat}*Tsm,SH_{Mat}*SR)	-0.14 (0.23)		0.62 (0.25)	0.17 (0.24)		-0.08 (0.45)		0.84 (0.50)	8	4362.6	4378.7	6.87	0.00
**SH_{Pat} Models**														
**1**	Φ (SH_{Pat},Twt,SR,SH_{Pat}*SR)		**0.67 (0.27)**	**0.58 (0.27)**	0.34 (0.24)				**1.01 (0.50)**	7	3891.7	3905.7	0.00	0.12
**2**	Φ (SH_{Pat},Tsm,Twt,SR,SH_{Pat}*SR)	-0.30 (0.25)	**0.72 (0.28)**	0.49 (0.28)	0.32 (0.24)				0.96 (0.50)	8	3890.2	3906.2	0.51	0.09
**3**	Φ (Tsm,Twt,SR)	-0.35 (0.24)	**0.70 (0.27)**	0.43 (0.27)						6	3895.3	3907.3	1.56	0.05
**4**	Φ (Twt,SR)		**0.65 (0.27)**	**0.53 (0.26)**						5	3897.4	3907.4	1.66	0.05
**5**	Φ (SH_{Pat},Twt,SR,SH_{Pat}*Twt,SH_{Pat}*SR)		**0.68 (0.27)**	**0.58 (0.27)**	0.38 (0.25)			0.30 (0.52)	0.93 (0.52)	8	3891.3	3907.4	1.67	0.05
**6**	Φ (SH_{Pat},SH_{Pat}^2,Twt,SR,SH_{Pat}*SR)		**0.67 (0.27)**	**0.58 (0.27)**	0.37 (0.26)	0.14 (0.37)			**1.07 (0.54)**	8	3891.5	3907.6	1.88	0.05
**7**	Φ (Tsm,Twt)	-0.45 (0.23)	**0.87 (0.26)**							5	3897.8	3907.8	2.11	0.04
**8**	Φ (SH_{Pat},Twt,SR)		**0.66 (0.27)**	**0.55 (0.26)**	0.29 (0.23)					6	3895.8	3907.9	2.14	0.04
**9**	Φ (SH_{Pat},Tsm,Twt,SR)	-0.34 (0.24)	**0.72 (0.27)**	0.45 (0.27)	0.27 (0.23)					7	3893.9	3908.0	2.22	0.04
**10**	Φ (SH_{Pat},Tsm,Twt,SR,SH_{Pat}*Twt, SH_{Pat} *SR)	-0.30 (0.25)	**0.72 (0.28)**	0.49 (0.28)	0.36 (0.25)			0.28 (0.51)	0.88 (0.52)	9	3889.9	3908.0	2.24	0.04
**11**	Φ (SH_{Pat},SH_{Pat}^2,Tsm,Twt,SR,SH_{Pat}*SR)	-0.30 (0.25)	**0.71 (0.28)**	0.49 (0.28)	0.35 (0.26)	0.13 (0.37)			1.02 (0.54)	9	3890.1	3908.1	2.40	0.03
**12**	Φ (SH_{Pat},Tsm,Twt,SR,SH_{Pat}*Tsm,SH_{Pat}*SR)	-0.31 (0.25)	**0.72 (0.28)**	0.49 (0.28)	0.32 (0.24)		0.18 (0.51)		1.00 (0.51)	9	3890.1	3908.1	2.40	0.03
**13**	Φ (SH_{Pat},Twt,SR,SH_{Pat}*Twt)		**0.68 (0.28)**	**0.55 (0.26)**	0.38 (0.25)			0.56 (0.50)		7	3894.6	3908.6	2.89	0.03
**14**	Φ (SH_{Pat},Tsm,Twt)	-0.44 (0.23)	**0.89 (0.26)**		0.24 (0.23)					6	3896.7	3908.8	3.03	0.03
**15**	Φ (SH_{Pat},Tsm,Twt,SR,SH_{Pat}*Twt)	-0.33 (0.24)	**0.73 (0.28)**	0.46 (0.27)	0.36 (0.25)			0.54 (0.49)		8	3892.7	3908.8	3.06	0.02
**16**	Φ (SH_{Pat},SH_{Pat}^2,Twt,SR, SH_{Pat}*Twt,SH_{Pat}*SR)		**0.68 (0.28)**	**0.57 (0.27)**	0.44 (0.28)	0.20 (0.39)		0.39 (0.57)	1.00 (0.55)	9	3891.1	3909.1	3.40	0.02
**17**	Φ (SH_{Pat},Tsm,Twt,SH_{Pat}*Twt)	-0.44 (0.23)	**0.91 (0.27)**		0.33 (0.25)			0.54 (0.50)		7	3895.6	3909.6	3.88	0.02

**18**	Φ (Twt)		**0.83 (0.26)**							4	3901.7	3909.7	3.97	0.02
**19**	Φ (SH_{Pat},SH_{Pat}^2,Tsm,Twt,SR,SH_{Pat}*Twt, SH_{Pat}*SR)	-0.30 (0.25)	**0.72 (0.28)**	0.49 (0.28)	0.41 (0.28)	0.19 (0.39)		0.36 (0.57)	0.95 (0.56)	10	3889.6	3909.7	4.00	0.02
**20**	Φ (SH_{Pat},SH_{Pat}^2,Twt,SR)		**0.67 (0.27)**	**0.55 (0.26)**	0.29 (0.24)	-0.03 (0.34)				7	3895.8	3909.9	4.15	0.01
**21**	Φ (SH_{Pat},Tsm,Twt,SR,SH_{Pat}*Tsm,SH_{Pat} *Twt, SH_{Pat} *SR)	-0.30 (0.25)	**0.72 (0.28)**	0.49 (0.28)	0.35 (0.25)		0.11 (0.53)	0.24 (0.54)	0.91 (0.55)	10	3889.8	3909.9	4.21	0.01
**22**	Φ (SH_{Pat},SH_{Pat}^2,Tsm,Twt,SR)	-0.34 (0.24)	**0.72 (0.27)**	0.45 (0.27)	0.27 (0.24)	-0.04 (0.34)				8	3893.9	3910.0	4.22	0.01
**23**	Φ (SH_{Pat},Tsm,Twt,SR,SH_{Pat}*Tsm)	-0.34 (0.24)	**0.72 (0.27)**	0.45 (0.27)	0.27 (0.23)		-0.03 (0.50)			8	3893.9	3910.0	4.23	0.01
**24**	Φ (SH_{Pat},SH_{Pat}^2,Tsm,Twt,SR,SH_{Pat}*Tsm, SH_{Pat}*SR)	-0.30 (0.25)	**0.72 (0.28)**	0.49 (0.28)	0.35 (0.26)	0.15 (0.37)	0.21 (0.52)		1.08 (0.56)	10	3889.9	3910.0	4.26	0.01
**25**	Φ (SH_{Pat},SR,SH_{Pat}*SR)			**0.78 (0.26)**	0.32 (0.24)				**1.04 (0.51)**	6	3898.2	3910.2	4.47	0.01
**26**	Φ (SH_{Pat},Twt)		**0.85 (0.26)**		0.26 (0.23)					5	3900.4	3910.4	4.71	0.01
**27**	Φ (SH_{Pat},SH_{Pat}^2,Twt,SR,SH_{Pat}*Twt)		**0.69 (0.28)**	**0.55 (0.26)**	0.40 (0.27)	0.09 (0.36)		0.61 (0.55)		8	3894.5	3910.6	4.84	0.01
**28**	Φ (SH_{Pat},Tsm,Twt,SR, SH_{Pat}*Tsm,SH_{Pat}*Twt)	-0.33 (0.25)	**0.73 (0.28)**	0.45 (0.27)	0.37 (0.25)		-0.14 (0.51)	0.56 (0.50)		9	3892.7	3910.7	5.01	0.01
**29**	Φ (SH_{Pat},Tsm,Twt,SH_{Pat}*Tsm)	-0.44 (0.23)	**0.89 (0.26)**		0.24 (0.23)		-0.07 (0.49)			7	3896.7	3910.8	5.02	0.01
**30**	Φ (SH_{Pat},SH_{Pat}^2,Tsm,Twt,SR,SH_{Pat}*Twt)	-0.33 (0.24)	**0.73 (0.28)**	0.45 (0.27)	0.38 (0.27)	0.08 (0.36)		0.59 (0.55)		9	3892.7	3910.8	5.02	0.01
**31**	Φ (SH_{Pat},SH_{Pat}^2,Tsm,Twt)	-0.44 (0.23)	**0.89 (0.26)**		0.24 (0.24)	-0.03 (0.34)				7	3896.7	3910.8	5.03	0.01
**32**	Φ (SH_{Pat},Twt, SH_{Pat}*Twt)		**0.87 (0.27)**		0.36 (0.25)			0.58 (0.51)		6	3899.1	3911.2	5.43	0.01
**33**	Φ (SH_{Pat},Tsm,SR,SH_{Pat}*SR)	-0.21 (0.25)		**0.74 (0.26)**	0.30 (0.24)				**1.00 (0.51)**	7	3897.4	3911.5	5.74	0.01
**34**	Φ (SH_{Pat},Tsm,Twt,SH_{Pat}*Tsm,SH_{Pat}*Twt)	-0.43 (0.23)	**0.90 (0.27)**		0.35 (0.25)		-0.20 (0.50)	0.58 (0.51)		8	3895.4	3911.5	5.74	0.01
**35**	Φ (SH_{Pat},SH_{Pat}^2,Tsm,Twt,SH_{Pat}*Twt)	-0.43 (0.23)	**0.91 (0.27)**		0.36 (0.27)	0.10 (0.36)		0.60 (0.56)		8	3895.5	3911.6	5.82	0.01
**36**	Φ (SR)			**0.73 (0.25)**						4	3903.7	3911.7	5.94	0.01

**37**	Φ (SH_{Pat},SH_{Pat}^2,Tsm,Twt,SR,SH_{Pat}*Tsm, SH_{Pat}*Twt, SH_{Pat}*SR)	-0.30 (0.25)	**0.72 (0.28)**	0.49 (0.28)	0.40 (0.28)	0.20 (0.39)	0.14 (0.54)	0.33 (0.59)	0.99 (0.58)	11	3889.6	3911.7	5.95	0.01
**38**	Φ (SH_{Pat},SH_{Pat}^2,Tsm,Twt,SR,SH_{Pat}*Tsm)	-0.34 (0.24)	**0.72 (0.27)**	0.45 (0.27)	0.27 (0.24)	-0.04 (0.34)	-0.03 (0.5)			9	3893.9	3912.0	6.24	0.01
**39**	Φ (SH_{Pat},SH_{Pat}^2,SR,SH_{Pat}*SR)			**0.78 (0.26)**	0.36 (0.26)	0.17 (0.39)			**1.12 (0.56)**	7	3898.0	3912.0	6.27	0.00
**40**	Φ (SH_{Pat},SR)			**0.75 (0.25)**	0.27 (0.24)					5	3902.4	3912.4	6.67	0.00
**41**	Φ (Tsm,SR)	-0.27 (0.24)		**0.69 (0.25)**						5	3902.4	3912.4	6.71	0.00
**42**	Φ (SH_{Pat},SH_{Pat}^2,Twt)		**0.85 (0.26)**		0.26 (0.24)	0.00 (0.34)				6	3900.4	3912.5	6.72	0.00
**43**	Φ (SH_{Pat},SH_{Pat}^2,Tsm,Twt,SR,SH_{Pat}*Tsm, SH_{Pat}*Twt)	-0.33 (0.25)	**0.73 (0.28)**	0.45 (0.27)	0.39 (0.27)	0.08 (0.37)	-0.13 (0.52)	0.61 (0.56)		10	3892.6	3912.7	6.97	0.00

Table S11 Plausible models, and their model selection statistics, of the effect of paternal (SH_{Pat}) standardised heterozygosity on their first-year survival probability, controlling for repeated measures within social groups. No. = model number; Tsm = mean summer temperature (May–October); Twt = mean winter temperature (November–February); SR = total summer rainfall (May–October); SH_{Pat}^2 = quadratic effect; * = interaction; k = number of parameters; QAICc = Akaike's information criterion, corrected for sample size and adjusted through quasi-likelihood; ΔQAICc = difference in QAICc from the top model (i.e., model with lowest QAICc); ω = relative QAICc weight (exp[-0.5 * ΔQAICc], divided by the sum of this quantity for all considered models, whether plausible or not); Φ = first-year survival probability. All plausible models (ΔQAICc < 7) are presented. Effect sizes (standard error) where the 95% confidence interval does not overlap zero are in bold. All predictors were standardised to a mean of 0 and a standard deviation of 2.

**No.**	**Model**	**Tsm (SE)**	**Twt (SE)**	**SR (SE)**	**SH (SE)**	**SH_{Ind}^2 (SE)**	**SH_{Ind}* Tsm (SE)**	**SH_{Ind}* Twt (SE)**	**SH_{Ind}* SR (SE)**	**k**	**Q Deviance**	**QAICc**	**Δ QAICc**	***ω***
**1**	Φ (Twt,SR, SH_{Pat}, SH_{Pat}*SR)		**0.67 (0.28)**	**0.55 (0.27)**	0.29 (0.24)				**1.01 (0.49)**	12	3996.0	4020.1	0.00	0.09
**2**	Φ (Tsm,Twt,SR, SH_{Pat}, SH_{Pat}*SR)	-0.35 (0.25)	**0.73 (0.28)**	0.44 (0.28)	0.27 (0.24)				0.95 (0.50)	13	3994.1	4020.3	0.16	0.08
**3**	Φ (Tsm,Twt)	**-0.50 (0.24)**	**0.89 (0.26)**							10	4000.6	4020.7	0.58	0.07
**4**	Φ (Tsm,Twt,SR)	-0.40 (0.25)	**0.73 (0.28)**	0.38 (0.27)						11	3998.6	4020.7	0.65	0.06
**5**	Φ (Twt,SR)		**0.66 (0.27)**	0.50 (0.26)						10	4001.2	4021.3	1.22	0.05
**6**	Φ (Twt,SR, SH_{Pat}, SR_{Pat}^2, SH_{Pat}*SR)		**0.66 (0.28)**	**0.55 (0.27)**	0.36 (0.27)	0.32 (0.38)			**1.17 (0.55)**	13	3995.2	4021.4	1.25	0.05
**7**	Φ (Tsm,Twt,SR, SH_{Pat}, SR_{Pat}^2, SH_{Pat}*SR)	-0.34 (0.25)	**0.72 (0.28)**	0.45 (0.28)	0.34 (0.27)	0.31 (0.38)			**1.10 (0.55)**	14	3993.4	4021.6	1.50	0.04
**8**	Φ (Twt,SR, SH_{Pat}, SH_{Pat}*Twt, SH_{Pat}*SR)		**0.67 (0.28)**	**0.54 (0.27)**	0.33 (0.25)			0.29 (0.51)	0.93 (0.52)	13	3995.6	4021.8	1.71	0.04
**9**	Φ (Tsm,Twt,SR, SH_{Pat})	-0.39 (0.25)	**0.74 (0.28)**	0.39 (0.27)	0.21 (0.24)					12	3997.8	4022.0	1.87	0.03

**10**	Φ (Tsm,Twt, SH_{Pat})	**-0.49** **(0.24)**	**0.91** **(0.26)**		0.19 (0.23)					11	3999.9	4022.0	1.92	0.03
**11**	Φ (Tsm,Twt,SR, SH_{Pat}, SH_{Pat}*Twt, SH_{Pat}*SR)	-0.34 (0.26)	**0.73** **(0.28)**	0.44 (0.28)	0.30 (0.25)			0.25 (0.51)	0.87 (0.53)	14	3993.8	4022.0	1.94	0.03
**12**	Φ (Tsm,Twt,SR, SH_{Pat}, SH_{Pat}*Tsm, SH_{Pat}*SR)	-0.35 (0.25)	**0.73** **(0.28)**	0.44 (0.28)	0.27 (0.24)		0.05 (0.52)		0.96 (0.51)	14	3994.1	4022.3	2.18	0.03
**13**	Φ (Twt,SR, SH_{Pat})		**0.67** **(0.27)**	**0.51** **(0.26)**	0.23 (0.24)					11	4000.3	4022.4	2.28	0.03
**14**	Φ (Tsm,Twt,SR, SH_{Pat}, SH_{Pat}*Twt)	-0.38 (0.25)	**0.75** **(0.28)**	0.40 (0.28)	0.29 (0.25)			0.54 (0.49)		13	3996.6	4022.8	2.66	0.02
**15**	Φ (Twt,SR, SH_{Pat}, SR_{Pat}^2, SH_{Pat}*Twt, SH_{Pat}*SR)		**0.67** **(0.28)**	**0.54** **(0.27)**	0.43 (0.29)	0.39 (0.40)		0.44 (0.58)	1.06 (0.57)	14	3994.6	4022.8	2.67	0.02
**16**	Φ (Tsm,Twt, SH_{Pat}, SH_{Pat}*Twt)	**-0.48** **(0.24)**	**0.92** **(0.27)**		0.28 (0.25)			0.54 (0.49)		12	3998.7	4022.8	2.70	0.02
**17**	Φ (Twt,SR, SH_{Pat}, SH_{Pat}*Twt)		**0.69** **(0.28)**	0.51 (0.26)	0.32 (0.25)			0.58 (0.49)		12	3998.9	4023.0	2.91	0.02
**18**	Φ (Tsm,Twt,SR, SH_{Pat}, SR_{Pat}^2, SH_{Pat}*Twt, SH_{Pat}*SR)	-0.33 (0.26)	**0.73** **(0.29)**	0.44 (0.28)	0.40 (0.29)	0.37 (0.4)		0.41 (0.58)	0.99 (0.57)	15	3992.9	4023.1	3.01	0.02
**19**	Φ (Twt)		**0.84** **(0.26)**							9	4005.1	4023.2	3.05	0.02
**20**	Φ (Tsm,Twt,SR, SH_{Pat}, SR_{Pat}^2, SH_{Pat}*Tsm, SH_{Pat}*SR)	-0.34 (0.25)	**0.73** **(0.28)**	0.45 (0.28)	0.34 (0.27)	0.31 (0.38)	0.11 (0.54)		**1.13** **(0.57)**	15	3993.4	4023.6	3.48	0.02
**21**	Φ (Tsm,Twt, SH_{Pat}, SH_{Pat}*Tsm)	**-0.48** **(0.24)**	**0.9** **(0.26)**		0.19 (0.23)		-0.21 (0.5)			12	3999.7	4023.9	3.77	0.01
**22**	Φ (Tsm,Twt,SR, SH_{Pat}, SR_{Pat}^2)	-0.39 (0.25)	**0.74** **(0.28)**	0.40 (0.27)	0.23 (0.25)	0.11 (0.34)				13	3997.7	4023.9	3.78	0.01
**23**	Φ (Tsm,Twt,SR, SH_{Pat}, SH_{Pat}*Tsm)	-0.38 (0.25)	**0.74** **(0.28)**	0.39 (0.27)	0.21 (0.24)		-0.17 (0.51)			13	3997.7	4023.9	3.79	0.01
**24**	Φ (Tsm,Twt, SH_{Pat}, SR_{Pat}^2)	**-0.49** **(0.24)**	**0.91** **(0.26)**		0.21 (0.25)	0.11 (0.34)				12	3999.8	4023.9	3.84	0.01
**25**	Φ (Tsm,Twt,SR, SH_{Pat}, SH_{Pat}*Tsm, SH_{Pat}*Twt, SH_{Pat}*SR)	-0.34 (0.26)	**0.73** **(0.28)**	0.43 (0.28)	0.30 (0.25)		-0.04 (0.55)	0.27 (0.54)	0.86 (0.55)	15	3993.8	4024.1	3.96	0.01
**26**	Φ (Tsm,Twt,SR, SH_{Pat}, SR_{Pat}^2, SH_{Pat}*Twt)	-0.38 (0.25)	**0.75** **(0.29)**	0.40 (0.28)	0.36 (0.28)	0.27 (0.38)		0.71 (0.57)		14	3996.0	4024.2	4.12	0.01
**27**	Φ (Twt,SR, SH_{Pat}, SR_{Pat}^2)		**0.67** **(0.27)**	**0.51** **(0.26)**	0.25 (0.25)	0.13 (0.34)				12	4000.1	4024.3	4.16	0.01
**28**	Φ (Tsm,Twt, SH_{Pat}, SR_{Pat}^2, SH_{Pat}*Twt)	**-0.48** **(0.24)**	**0.92** **(0.27)**		0.35 (0.28)	0.27 (0.37)		0.72 (0.58)		13	3998.1	4024.3	4.16	0.01

**29**	Φ (SR, SH_{Pat}, SH_{Pat}*SR)			**0.76 (0.26)**	0.26 (0.24)				**1.06 (0.5)**	11	4002.2	4024.3	4.22	0.01
**30**	Φ (Twt, SH_{Pat})		**0.86 (0.26)**		0.21 (0.23)					10	4004.2	4024.3	4.25	0.01
**31**	Φ (Tsm,Twt, SH_{Pat}, SH_{Pat}*Tsm, SH_{Pat}*Twt)	0.30 (0.25)	**0.90 (0.27)**		0.30 (0.25)		-0.35 (0.52)	0.62 (0.50)		13	3998.2	4024.4	4.25	0.01
**32**	Φ (Twt,SR, SH_{Pat}, SR_{Pat}^2, SH_{Pat}*Twt)		**0.28 (0.38)**	0.51 (0.26)	0.39 (0.27)	0.28 (0.38)		0.75 (0.58)		13	3998.3	4024.4	4.32	0.01
**33**	Φ (Tsm,Twt,SR, SH_{Pat}, SH_{Pat}*Tsm, SH_{Pat}*Twt)	-0.37 (0.25)	**0.75 (0.28)**	0.38 (0.28)	0.31 (0.25)		-0.30 (0.52)	0.60 (0.5)		14	3996.3	4024.5	4.36	0.01
**34**	Φ (Twt, SH_{Pat}, SH_{Pat}*Twt)		**0.87 (0.27)**		0.31 (0.25)			0.60 (0.49)		11	4002.8	4024.9	4.78	0.01
**35**	Φ (Tsm,Twt,SR, SH_{Pat}, SR_{Pat}^2, SH_{Pat}*Tsm, SH_{Pat}*Twt, SH_{Pat}*SR)	-0.33 (0.26)	**0.73 (0.29)**	0.44 (0.28)	0.40 (0.29)	0.37 (0.40)	-0.01 (0.57)	0.41 (0.61)	0.99 (0.6)	16	3992.9	4025.1	5.04	0.01
**36**	Φ (SR, SH_{Pat}, SR_{Pat}^2, SH_{Pat}*SR)			**0.77 (0.26)**	0.36 (0.28)	0.38 (0.41)			**1.25 (0.57)**	12	4001.2	4025.4	5.28	0.01
**37**	Φ (Tsm,SR, SH_{Pat}, SH_{Pat}*SR)	-0.24 (0.25)		**0.71 (0.26)**	0.25 (0.24)				**1.02 (0.5)**	12	4001.3	4025.4	5.33	0.01
**38**	Φ (SR)			**0.72 (0.25)**						9	4007.5	4025.5	5.44	0.01
**39**	Φ (Tsm,Twt, SH_{Pat}, SR_{Pat}^2, SH_{Pat}*Tsm)	**-0.48 (0.24)**	**0.90 (0.26)**		0.21 (0.25)	0.10 (0.34)	-0.19 (0.51)			13	3999.6	4025.8	5.71	0.01
**40**	Φ (Tsm, Twt,SR, SH_{Pat}, SR_{Pat}^2, SH_{Pat}*Tsm)	-0.38 (0.25)	**0.74 (0.28)**	0.39 (0.27)	0.23 (0.25)	0.10 (0.34)	-0.15 (0.52)			14	3997.6	4025.8	5.72	0.01
**41**	Φ (Tsm, Twt, SH_{Pat}, SR_{Pat}^2, SH_{Pat}*Tsm, SH_{Pat}*Twt)	-0.46 (0.24)	**0.91 (0.27)**		0.37 (0.28)	0.27 (0.38)	-0.35 (0.53)	0.79 (0.59)		14	3997.7	4025.8	5.75	0.00
**42**	Φ (Tsm, Twt,SR, SH_{Pat}, SR_{Pat}^2, SH_{Pat}*Tsm, SH_{Pat}*Twt)	-0.37 (0.25)	**0.75 (0.29)**	0.38 (0.28)	0.38 (0.28)	0.27 (0.38)	-0.29 (0.54)	0.76 (0.58)		15	3995.7	4026.0	5.86	0.00
**43**	Φ (Tsm,SR)	-0.3 (0.25)		**0.67 (0.25)**						10	4006.0	4026.1	6.00	0.00
**44**	Φ (Twt, SH_{Pat}, SR_{Pat}^2)		**0.85 (0.26)**		0.24 (0.25)	0.14 (0.34)				11	4004.1	4026.2	6.09	0.00
**45**	Φ (Twt, SH_{Pat}, SR_{Pat}^2, SH_{Pat}*Twt)		**0.88 (0.27)**		0.39 (0.28)	0.30 (0.37)		0.79 (0.58)		12	4002.1	4026.2	6.10	0.00
**46**	Φ (Tsm,SR, SH_{Pat}, SR_{Pat}^2, SH_{Pat}*SR)	-0.24 (0.25)		**0.73 (0.26)**	0.34 (0.28)	0.38 (0.41)			**1.21 (0.57)**	13	4000.3	4026.5	6.41	0.00
**47**	Φ (SR, SH_{Pat})			**0.73 (0.25)**	0.20 (0.24)					10	4006.8	4026.9	6.76	0.00

Table S12 Model averaged estimates of paternal standardised heterozygosity (SH_{Pat}) effects on their first-year survival probability (Φ), controlling for repeated measures within social groups, using natural average and zero methods (Burnham and Anderson 2002). Effect sizes where the 95% confidence interval (CI) does not overlap zero are in bold. No. = sequential numbering of each model averaged estimate; Relative importance = sum of Akaike weights of models that contain the effect of interest; SR = total summer rainfall (May–October); Tsm = mean summer temperature (May–October); Twt = mean winter temperature (November–February); SH_{Pat}^2 = quadratic effect; * = interaction effect. All predictors were standardised to a mean of 0 and a standard deviation of 2.

Fixed effect	Natural average method		Zero method		
	Estimate	95% CI	Estimate	95% CI	Relative importance
SR	0.48	-0.08, 1.04	0.37	-0.25, 1.00	0.77
Tsm	-0.39	-0.91, 0.14	-0.24	-0.79, 0.31	0.61
Twt	**0.75**	**0.17, 1.32**	**0.72**	**0.11, 1.32**	**0.95**
SH_{Pat}	0.29	-0.20, 0.79	0.23	-0.27, 0.74	0.79
SH_{Pat}^2	0.28	-0.48, 1.04	0.07	-0.26, 0.41	0.26
SH_{Pat}*SR	1.01	-0.03, 2.05	0.47	-0.71, 1.65	0.46
SH_{Pat}*Tsm	-0.11	-1.18, 0.97	-0.01	-0.18, 0.15	0.13
SH_{Pat}*Twt	0.48	-0.61, 1.57	0.13	-0.41, 0.67	0.27

Table S13 The estimated effect sizes (with 95% credible intervals, from MCMC) of A) individual (SH_{Ind}), maternal (SH_{Mat}) and paternal (SH_{Pat}) multilocus heterozygosity, and B) individual (SLH_{Ind}), maternal (SLH_{Mat}) and paternal (SLH_{Pat}) single-locus heterozygosity on the probability of offspring surviving to age one and their interaction (*) with SR [total summer rainfall (May–October)]. Estimates whose 95% confidence interval does not overlap zero are shown in bold.

**A) Multilocus**	**SH_{Ind}**	**SH_{Mat}**	**SH_{Pat}**
SH	0.22 (-0.11, 0.66)	0.11 (-0.26, 0.65)	0.29 (-0.16,0.80)
SH*SR	0.53 (-0.30, 1.33)	0.90 (-0.16,1.72)	**0.84 (0.02, 1.92)**

**B) Single-locus^**	**SLH_{Ind}**	**SLH_{Mat}**	**SLH_{Pat}**
Mel-1	0.13 (-0.49, 0.89)	0.46 (-0.49, 1.35)	-0.10 (-0.92, 1.03)
Mel-4	-0.06 (-0.66, 0.51)	**1.28 (0.39, 2.48)**	-0.70 (-1.93, 0.21)
Mel-10	0.48 (-0.09, 1.27)	0.62 (-0.66, 1.97)	1.01 (-0.24, 2.29)
Mel-12	-0.52 (-1.14, 0.18)	0.01 (-1.26, 1.10)	0.43 (-0.58, 1.82)
Mel-14	-0.47 (-1.05, 0.15)	-0.27 (-1.40, 0.97)	0.46 (-0.51, 1.48)
Mel-15	-0.11 (-0.78, 0.44)	-0.63 (-1.84, 0.17)	-0.67 (-1.75, 0.44)
Mel-101	0.54 (-0.13, 1.10)	**-1.70 (-2.94, -0.95)**	0.06 (-1.05, 1.12)
Mel-102	**0.69 (0.16, 1.31)**	**2.13 (1.17, 3.48)**	0.49 (-0.51, 1.63)
Mel-103	-0.18 (-0.88, 0.37)	-0.07 (-1.09, 0.94)	0.76 (-0.11, 1.82)
Mel-104	-0.25 (-0.94, 0.27)	-0.39 (-1.59, 0.70)	**-1.42 (-2.87, -0.19)**
Mel-105	-0.25 (-0.96, 0.34)	0.43 (-0.82, 1.61)	0.67 (-0.50, 1.65)
Mel-106	0.01 (-0.61, 0.63)	-1.12 (-2.21, 0.00)	0.07 (-0.96, 1.22)
Mel-107	0.16 (-0.40, 0.84)	-0.28 (-1.60, 0.54)	-0.40 (-1.49, 0.52)
Mel-108	0.36 (-0.24, 1.09)	0.85 (-0.29, 2.31)	-0.32 (-1.45, 0.65)
Mel-109	0.37 (-0.16, 1.04)	-0.48 (-1.83, 0.34)	**-1.58 (-2.70, -0.48)**
Mel-110	**0.69 (0.15, 1.37)**	0.71 (-0.20, 1.90)	0.20 (-0.87, 1.21)
Mel-111	0.17 (-0.43, 0.78)	0.52 (-0.72, 2.05)	**1.17 (0.02, 2.78)**
Mel-112	0.41 (-0.07, 1.11)	0.00 (-0.97, 0.89)	**-1.53 (-3.03, -0.37)**
Mel-113	0.31 (-0.23, 1.14)	0.63 (-0.52, 1.86)	**2.03 (1.01, 3.74)**
Mel-115	**0.77 (0.17, 1.40)**	**1.72 (0.78, 3.05)**	**1.42 (0.42, 2.70)**
Mel-116	-0.09 (-0.74, 0.40)	0.69 (-0.28, 1.94)	-0.70 (-1.88, 0.48)
Mel-117	-0.10 (-0.69, 0.53)	-0.43 (-1.64, 1.04)	0.77 (-0.25, 1.85)
Mel-126	-0.41 (-0.89, 0.25)	0.53 (-0.44, 1.57)	0.91 (-0.20, 2.13)
Mel-127	**0.73 (0.25, 1.62)**	0.15 (-0.99, 1.32)	0.16 (-0.76, 1.28)
Mel-129	-0.38 (-1.04, 0.18)	0.48 (-0.45, 1.49)	-0.58 (-1.74, 0.51)
Mel-131	0.06 (-0.46, 0.66)	0.31 (-0.61, 1.41)	-0.62 (-1.66, 0.49)
Mel-137	**0.52 (0.05, 1.41)**	0.38 (-0.66, 1.58)	**1.61 (0.35, 2.58)**
Mel-140	0.06 (-0.60, 0.61)	-0.28 (-1.40, 0.94)	-0.03 (-1.03, 1.04)

Mel-153	-0.38 (-0.96, 0.25)	0.77 (-0.19, 2.17)	0.42 (-0.59, 1.42)
Mel-161	-0.40 (-1.02, 0.15)	-0.64 (-1.98, 0.32)	0.39 (-0.39, 1.46)
Mel-186	-0.21 (-0.83, 0.33)	-0.59 (-1.54, 0.44)	**-1.20 (-2.18, -0.14)**
Mel-191	-0.32 (-0.86, 0.34)	0.40 (-0.77, 1.55)	0.34 (-0.76, 1.65)
Mel-1*SR	-0.34 (-1.57, 0.90)	0.58 (-1.15, 2.33)	0.49 (-1.16, 2.51)
Mel-4*SR	0.53 (-0.62, 1.75)	0.30 (-1.45, 2.66)	-0.48 (-2.46, 1.65)
Mel-10*SR	-0.24 (-1.56, 1.02)	-0.39 (-2.11, 2.27)	-0.18 (-2.54, 1.49)
Mel-12*SR	-0.74 (-2.13, 0.38)	1.66 (-0.91, 3.77)	1.01 (-0.89, 3.15)
Mel-14*SR	0.71 (-0.39, 1.93)	-0.53 (-3.22, 1.41)	0.48 (-1.35, 2.32)
Mel-15*SR	1.18 (-0.21, 2.18)	0.91 (-0.55, 3.19)	-1.18 (-3.21, 0.80)
Mel-101*SR	-0.43 (-1.51, 0.97)	-0.64 (-2.53, 1.25)	-0.54 (-2.43, 1.36)
Mel-102*SR	-0.05 (-1.10, 1.21)	0.44 (-1.58, 2.64)	0.63 (-1.30, 2.53)
Mel-103*SR	-0.25 (-1.64, 0.83)	0.54 (-1.33, 2.89)	-1.02 (-3.00, 0.78)
Mel-104*SR	0.75 (-0.83, 1.79)	0.31 (-1.57, 2.32)	1.66 (-0.52, 3.88)
Mel-105*SR	0.34 (-0.99, 1.50)	0.76 (-1.41, 2.78)	-0.03 (-1.60, 2.00)
Mel-106*SR	0.12 (-1.15, 1.29)	1.15 (-1.24, 2.71)	-0.32 (-2.31, 1.80)
Mel-107*SR	-0.42 (-1.42, 0.99)	-0.77 (-3.21, 1.28)	-0.48 (-2.32, 1.44)
Mel-108*SR	-0.26 (-1.68, 0.79)	0.30 (-2.17, 2.53)	-0.31 (-2.27, 1.32)
Mel-109*SR	-0.09 (-1.39, 0.99)	1.56 (-0.61, 3.91)	0.46 (-1.59, 2.52)
Mel-110*SR	1.01 (-0.27, 2.12)	0.74 (-1.64, 2.33)	0.23 (-1.52, 1.93)
Mel-111*SR	0.28 (-0.95, 1.47)	0.98 (-1.03, 3.48)	1.54 (-0.89, 3.23)
Mel-112*SR	-0.03 (-1.09, 1.12)	-1.11 (-3.01, 0.79)	-0.67 (-2.32, 1.63)
Mel-113*SR	0.87 (-0.39, 2.30)	0.84 (-1.20, 3.10)	**3.28 (0.78, 5.45)**
Mel-115*SR	0.40 (-0.56, 1.71)	0.48 (-1.52, 2.49)	0.66 (-1.06, 2.53)
Mel-116*SR	-0.24 (-1.31, 0.99)	-1.42 (-3.61, 0.91)	1.75 (-0.51, 3.59)
Mel-117*SR	0.34 (-0.77, 1.50)	-0.50 (-2.65, 1.67)	0.30 (-1.52, 2.34)
Mel-126*SR	-0.02 (-0.98, 1.21)	0.19 (-1.78, 2.29)	0.75 (-1.45, 2.52)
Mel-127*SR	0.88 (-0.33, 2.13)	0.45 (-1.74, 2.77)	-0.83 (-2.40, 1.59)
Mel-129*SR	-0.69 (-1.72, 0.61)	-0.21 (-1.92, 1.94)	-1.74 (-3.88, 0.11)
Mel-131*SR	0.38 (-0.67, 1.66)	-0.73 (-3.15, 1.04)	0.13 (-1.77, 1.73)
Mel-137*SR	**1.45 (0.33, 2.77)**	1.12 (-1.31, 2.84)	-0.15 (-2.63, 1.76)
Mel-140*SR	0.22 (-0.99, 1.26)	**2.37 (0.26, 4.32)**	0.89 (-1.21, 2.63)
Mel-153*SR	-0.38 (-1.69, 0.59)	0.58 (-1.25, 2.87)	-1.47 (-3.29, 0.31)
Mel-161*SR	0.10 (-1.17, 1.16)	-0.31 (-2.32, 2.03)	1.39 (-0.59, 3.22)
Mel-186*SR	**-1.57 (-2.53, -0.24)**	0.71 (-1.46, 2.37)	-0.69 (-2.42, 1.39)
Mel-191*SR	0.15 (-1.08, 1.27)	0.83 (-1.30, 2.84)	-0.11 (-2.03, 1.97)

^ Locus *Mel*-114 was excluded from these analyses, because only six cubs, three mothers, and two fathers were heterozygous at this marker. Loci *Mel*-135 and *Mel*-138 were also excluded due to collinearity: These had variance inflation factors of 6.97 and 7.12, respectively (Zuur et al. 2010).

Table S14 The interaction effect of paternal heterozygosity (SH_{Pat}) with rainfall on first-year survival probability (Φ) with and without a measure of an individual's father's rare alleles as a covariate. Alleles that had a frequency of < 5% were defined as rare alleles. For each individual we calculated the number of copies of rare alleles that their father had, divided by the number of alleles for which their father was typed (= twice the number of loci typed). k = number of parameters; QAICc = Akaike's information criterion, corrected for sample size and adjusted through quasi-likelihood; Δ = difference in QAICc from the top model (i.e., model with lowest QAICc); ω = relative QAICc weight (exp[-0.5 * ΔQAICc], divided by the sum of this quantity for all considered models). Twt = mean winter temperature (November–February); SR = total summer rainfall (May–October). * = interaction. β = parameter estimate. Effect sizes where the 95% confidence interval (CI) does not overlap zero are in bold.

Model	k	Deviance	QAICc	Δ	*ω*	Model likelihood	β (95% confidence intervals)
Φ (Twt, SR, SH_{Pat}, SH_{Pat} *SR)	7	3891.7	3905.7	0.00	0.59	1.00	SH_{Pat}*SR: **1.01 (0.05, 1.97)**
Φ (Twt, SR, SH_{Pat}, SH_{Pat} *SR, rare alleles)	8	3890.4	3906.5	0.72	0.41	0.70	SH_{Pat}*SR: **1.06 (0.08, 2.05)**<br>rare alleles: -0.29 (-0.77, 0.19)

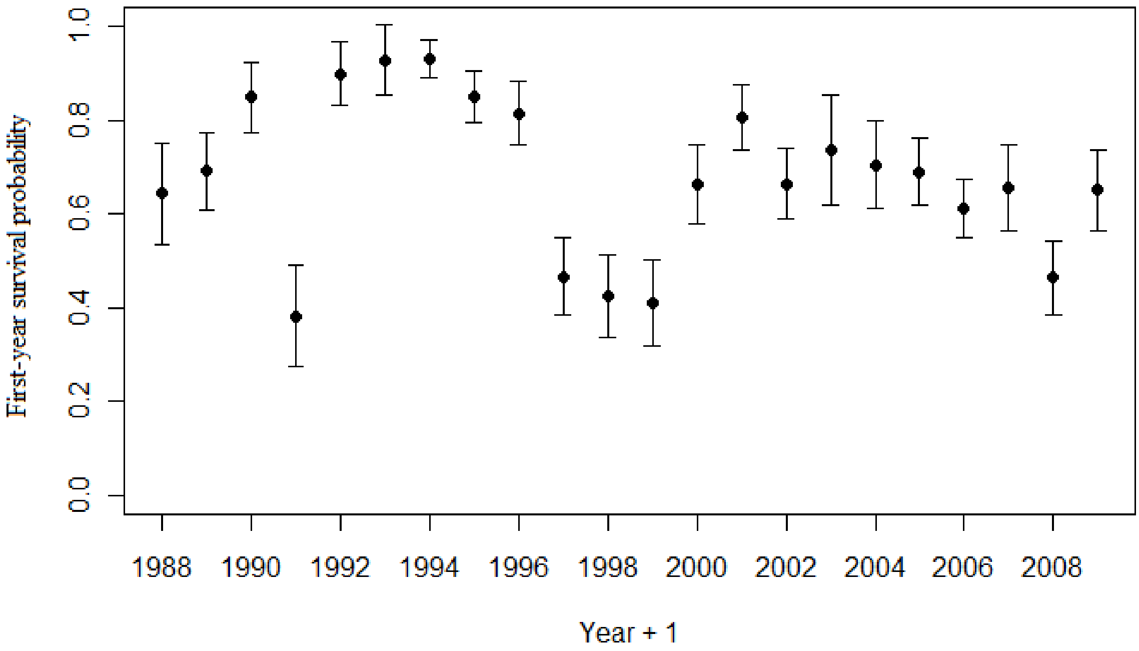

Figure 1 Estimated first-year survival probabilities (± SE) for cub cohorts from 1988 to 2009 (N = 975). Survival estimates were derived by model averaging.

References

Annavi G, Dawson DA, Horsburgh GJ, Greig C, Dugdale HL, Newman C, Macdonald DW, Burke T (2011) Characterisation of twenty-one European badger (*Meles meles*) microsatellite loci facilitates the discrimination of second-order relatives. *Conservation Genetic Resources*, **3**, 515–518.

Burnham K P, Anderson D R (2002) Model selection and multimodel inference. Springer Verlag, New York, NY.

Bijlsma R, Van de Vliet M, Pertoldi C, Van Apeldoorn RC, Van de Zande L (2000) Microsatellite primers from the Eurasian badger, *Meles meles*. *Molecular Ecology*, **9**, 2216–2217.

Carpenter PJ, Dawson DA, Greig C, Parham A, Cheeseman CL, Burke T (2003) Isolation of 39 polymorphic microsatellite loci and the development of a fluorescently labelled marker set for the Eurasian badger (*Meles meles*) (Carnivora: Mustelidae). *Molecular Ecology Notes*, **3**, 610–615.

Domingo-Roura X, Macdonald DW, Roy MS, Marmi J, Terradas J, Woodroffe R, Burke T, Wayne RK (2003) Confirmation of low genetic diversity and multiple breeding females in a social group of Eurasian badgers from microsatellite and field data. *Molecular Ecology*, **12**, 533–539.

Hadfield JD, Richardson DS, Burke T (2006) Towards unbiased parentage assignment: combining genetic, behavioural and spatial data in a Bayesian framework. *Molecular Ecology*, **15**, 3715–3730.

Huck M, Frantz AC, Dawson DA, Burke TJ, Roper T (2008) Low genetic variability, female-biased dispersal and high movement rates in an urban population of Eurasian badgers *Meles meles*. *Journal of Animal Ecology*, **77**, 905–915.

Kalinowski ST, Taper ML, Marshall TC (2007) Revising how the computer program CERVUS accommodates genotyping error increases success in paternity assignment. Molecular Ecology, 16, 1099–1106.

Morrissey MB, Wilson AJ, Pemberton JM, Ferguson MM (2007) A framework for power and sensitivity analyses for quantitative genetic studies of natural populations, and case studies in Soay sheep *(Ovis aries)*. *Journal of Evolutionary Biology*, **20**, 2309–2321.

Van Oosterhout C, Hutchinson WF, Wills DPM, Shipley P (2004) MICRO-CHECKER: software for identifying and correcting genotyping errors in microsatellite data. *Molecular Ecology Notes*, **4**, 535–538.

Wang J, Santure AW (2009) Parentage and sibship inference from multilocus genotype data under polygamy. *Genetics*, **181**, 1579–1594.

Zuur AF, Ieno EN, Elphick CS (2010) A protocol for data exploration to avoid common statistical problems. *Methods in Ecology and Evolution*, **1**, 3–14.
